# Improved oncolytic and immunostimulatory activity of the spontaneous *jin-3* reovirus mutant in preclinical bladder cancer models

**DOI:** 10.1016/j.omton.2026.201128

**Published:** 2026-01-13

**Authors:** Arjanneke F. van de Merbel, Maaike H. van der Mark, Lobke C.M. Hensen, Diana J.M. van den Wollenberg, Rob C. Hoeben, Willemijn C.G. Zonneveld, Rob C.M. Pelger, Maxime T.M. Kummeling, Geertje van der Horst, Gabri van der Pluijm

**Affiliations:** 1 Department of Urology, Leiden University Medical Center, Albinusdreef 2, Leiden 2333ZA, the Netherlands; 2 Department of Cell and Chemical Biology, Leiden University Medical Center, Albinusdreef 2, Leiden 2333ZA, the Netherlands

**Keywords:** bladder cancer, oncolytic viruses, immunotherapy, PDX models, three-dimensional cultures, *jin-3*, reovirus, *ex vivo* explants, tumour-immune cell co-culture, immunogenic cell death

## Abstract

Immunotherapy has emerged as a promising strategy for treatment of urothelial carcinoma of the bladder (UCB) but not all patients show clinically desirable responses upon treatment with immune-checkpoint inhibition. Oncolytic viruses may unleash the full potential of immunotherapy. Besides tumor-lysing properties, oncolytic viruses can induce durable and systemic anti-viral and anti-tumor immune responses. Here, we evaluated and compared oncolytic and immunostimulatory properties of wild-type reovirus (R124) and the junction-adhesion molecule-independent mutant reovirus (*jin-3*) in preclinical models of human UCB. Both reovirus variants effectively infected and replicated in human UCB cells and induced cell lysis in flatbottom or 3D cultures and in *ex vivo* cultured human tumor tissue slices. However, *jin-3* reovirus demonstrated greater efficacy in a dose-dependent manner, effectively inducing expression of immunogenic cell death markers, interferon (IFN)-stimulated genes, and inflammatory cytokines. To study interactions between tumor and immune cells, we established a co-culture system. In this context, co-culturing reovirus-infected bladder tumoroids with peripheral blood mononuclear cells was found to significantly enhance cancer death dose-dependently, alongside induction of key immune mediators such as CXCL10 and IFN-γ. Together, reoviruses display strong oncolytic properties in preclinical UCB models. *jin-3* reoviruses elicit robust immunostimulatory responses, highlighting their potential as candidate agents for clinical translation in UCB.

## Graphical abstract

**Figure:**
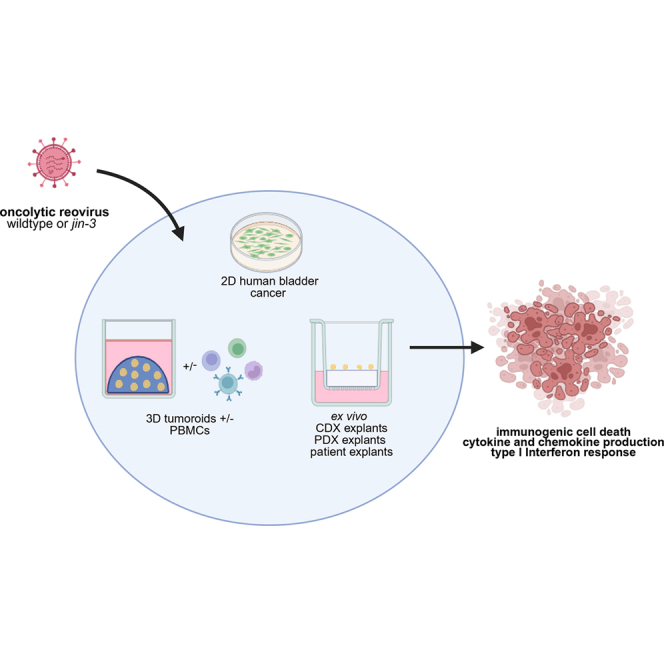

## Teaser

This study describes a mutant reovirus *jin-3* that enhances immune activation while effectively killing human bladder cancer cells in preclinical models. Oncolytic reoviruses, particularly *jin-3*, may boost immunotherapy effectiveness, offering a promising new strategy for bladder cancer patients who poorly respond to current immunotherapeutic approaches.

## Introduction

Urothelial carcinoma of the bladder (UCB) is the seventh most prevalent cancer globally, with a 5-year prevalence surpassing 5 million cases.^1^ Approximately 75% of newly diagnosed cases encompass non-muscle invasive bladder cancer (NMIBC), which are usually removed by transurethral resection.^2^ Patients are then categorized as low, intermediate, or high risk for recurrence and progression according to the guidelines of the European Urological Association.^3^ Current therapy for patients with intermediate- and high-risk NMIBC includes immunotherapy with Bacillus Calmette-Guérin (BCG) or chemotherapy. To date, BCG instillations have proven to be the most successful adjuvant therapy.^3^^,^^4^ However, 30%–50% of the patients fail to respond to BCG therapy, and 15% of patients show progression to the muscle invasive disease, MIBC.^2^^,^^5^^,^^6^ These MIBC patients have a relatively poor prognosis (5-year survival of 40%–60%). UCB recurs frequently after initial treatment and requires long-term clinical monitoring, causing substantial morbidity and diminished quality of life.^4^^,^^7^^,^^8^ Immunotherapy (including immune checkpoint inhibition [ICI], cell-based therapy, and cancer vaccines) is most effective in tumors with relatively high somatic mutation rates as is the case for UCB.^9^^,^^10^^,^^11^^,^^12^^,^^13^^,^^14^^,^^15^

ICIs, including pembrolizumab, nivolumab, atezolizumab, durvalumab, and avelumab, have improved the treatment landscape for bladder cancer, particularly for metastatic urothelial carcinoma.^16^

These ICIs have shown impressive and durable responses in bladder cancer patients.^17^^,^^18^^,^^19^^,^^20^^,^^21^^,^^22^^,^^23^^,^^24^^,^^25^^,^^26^^,^^27^^,^^28^ In high-risk NMIBC patients who are unresponsive to BCG, 41% of patients display a clinically desirable response to pembrolizumab (i.e., absence of high-risk NMIBC or progressive disease after 3 months).^29^ About 30% of patients with metastatic MIBC will respond to ICI treatment (objective response rate and overall survival),^30^ although these benefits appear to be limited to patients with pre-existing anti-tumor responses.^25^^,^^31^^,^^32^^,^^33^

Currently, ICIs are standard-of-care options in multiple clinical scenarios: as first-line therapy for cisplatin-ineligible patients, second-line therapy after platinum-based chemotherapy failure, and in novel combination regimens.

Despite these encouraging results in a subgroup of UCB patients, treatment modalities that can further improve immunotherapy are urgently required for unresponsive bladder cancer patients.

Oncolytic viruses represent a possible new approach since these viruses can infect, replicate, and lyse malignant tumor cells, while minimizing harm to normal cells.^34^ Furthermore, oncolytic viruses can activate the adaptive and innate immune system^35^^,^^36^^,^^37^^,^^38^ via the induction of immunogenic cell death (ICD), a type of cell death that triggers immunity against tumor antigens of dying cells, resulting in the exposure of damage-associated molecular patterns (DAMPs), e.g., HMGB1 release.^39^^,^^40^ DAMPs can activate antigen-presenting cells, which subsequently can stimulate the expansion and activation of effector T cells, including cytotoxic CD8+, helper CD4+ T cells, and natural killer (NK) cells.^41^^,^^42^

Reoviruses are double-stranded RNA viruses and have not been associated with severe disease in humans.^43^ Mutant reovirus *jin-3* harbors a mutation in the spike protein Sigma-1 and can infect cells via the high-affinity receptor junction adhesion molecule A (JAM-A) but also independently of JAM-A on the tumor cell surface by high-affinity binding to negatively charged sialic acids. We previously demonstrated that *jin-3* reoviruses display extended tumor tropism compared to the wild-type reovirus.^44^^,^^45^ Interestingly, this JAM-A protein is involved in various biological processes including tight junctions involved in epithelial barrier function.^46^ Expression levels of JAM-A in various cancers have been analyzed using multiple databases, including TIMER2.0, GEPIA2, and the UALCAN portal and was shown to be upregulated in multiple cancers.^47^ JAM-A was significantly upregulated in urothelial carcinoma compared to normal bladder. However, bladder cancer patients with low JAM-A expression showed decreased overall survival.^47^ Potentially, this reduced overall survival could be due to the acquisition of a more mesenchymal invasive phenotype (EMT) by the cancer cells due to the shedding of epithelial tight junctions.^47^ Mutational profile analysis (cBioportal based on TCGA datasets) revealed that JAM-A was altered in more than 15% of bladder cancer patients experiencing either amplification or mutation of JAM-A.^47^

In this study, the direct oncolytic and indirect immunomodulatory effects of reovirus mutant *jin-3* were determined and compared to those of the wild-type T3D reovirus R124 in state-of-the-art preclinical bladder cancer models, including monolayer cultures and three-dimensional cell (co-) cultures and *ex vivo* cultured bladder tumor tissue slices derived from patients with disease ranging from low-grade NMIBC to MIBC.

## Results

### Direct cytolytic effects of *jin-3* and R124 reoviruses

**Figure 1:**
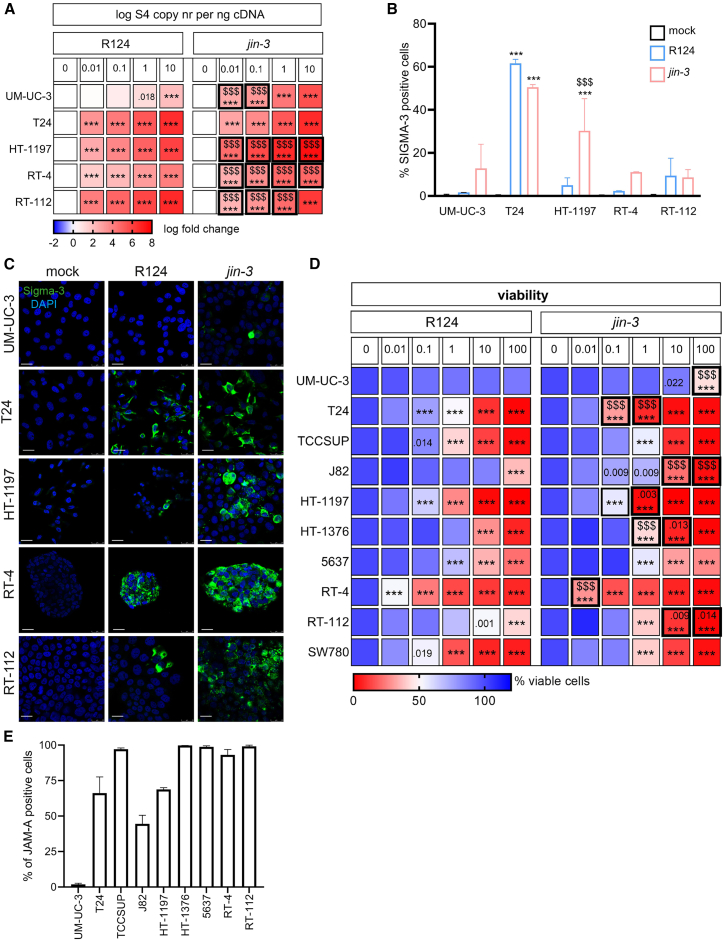
Comparison of the oncolytic effects of mutant *jin-3* versus R124 reovirus in bladder cancer cell lines *in vitro* (A) Mean viral S4Q copy number (log fold change S4Q mRNA expression vs. mock treated cells) in bladder cancer cell lines exposed to a range of MOI (0.01–0.1–1–10) of either R124 or *jin-3* reoviruses after 24 h. In red, the highest infection with virus is shown. Two-way ANOVA followed by Tukey’s post hoc test. *n* = 3 (2 replicates). UM-UC-3 cells R124 reovirus MOI1 vs. mock *p* = 0.0184; ∗∗∗*p* < .001, $$$ *p* < .001, asterisks indicate mock versus reovirus infection, and dollar signs indicate R124 versus *jin-3.* (B) Viral load (% of viable, single cells expressing Sigma-3 protein) in bladder cancer cell lines exposed to MOI10 of either R124 or *jin-3* after 72 h mean (SD) (*N* = 3). Two-way ANOVA followed by Tukey’s post hoc test. ∗∗∗*p* < .001, $$$ *p* < .001, asterisks indicate mock versus reovirus infection, and dollar signs indicate R124 versus *jin-3.* (C) Confocal images of viral protein (Sigma-3, green) and DAPI (blue)-stained bladder cancer cells treated with MOI10 of R124 or *jin-3* for 72h. Scale bars, 25 μm. (D) Mean percentage of viable cells after exposure to a range of MOI of either R124 or *jin-3* for 6 days. *n* = 3 (6 replicates). *p* values are depicted (vs. mock; when 2 depicted upper *p* value is R124 vs. *jin-3*); ∗∗∗*p* < .001, $$$ *p* < .001, asterisks indicate mock versus reovirus infection, and dollar signs indicate R124 versus *jin-3.* (E) Percentage of single, viable, JAM-A protein expressing cells. Mean (SD), *n* = 3. MOI = multiplicity of infection.

A panel of human bladder cancer cell lines of different molecular subtypes was exposed to a dose range of either R124 or *jin-3*, and viral expression levels were detected by RT-PCR after 24 h. In the UM-UC-3, HT-1197, and RT-4 cell lines, more viral transcripts were detected in the cells exposed to *jin-3*, whereas in the T24 and RT-112 cell lines either no difference or less viral transcripts were found (Figures 1A and S1A–S1C; Table S1). In addition, viral protein levels of Sigma-3 were determined in various tumor cells by flow cytometry (Figure 1B) or immunofluorescence (Figure 1C) after 72 h of treatment. A similar Sigma-3 pattern was observed, i.e., increased level of expression in *jin-3*-treated UM-UC3, HT-1197, and RT-4 and a similar expression level of Sigma-3 in T24 and RT-112 cell lines.

Both *jin-3* and R124 effectively killed the majority of bladder cancer cells in a dose- and time-dependent manner (Figures 1D and S1D). In cell lines of different type (MIBC or NMIBC; see Table S1) and molecular cancer subtypes, *jin-3* and R124 displayed comparable anti-tumor effects in 5637 (mixed phenotype), RT-4 (luminal phenotype), and SW780 (basal phenotype). In the HT-1197, HT-1376 (both mixed phenotype), and RT-112 cells (luminal phenotype), *jin-3* was found to be more effective.^48^ In the basal molecular subtype bladder cancer cell panel, T24 cells were significantly more effectively killed by *jin-3* compared with wild-type reovirus R124, while both viruses were equally effective in TCCSUP cells. Strikingly, while R124 viruses did not affect the viability of the basal type UM-UC-3 cells, *jin-3* reduced their viability. Moreover, in J82 cells, R124 viruses resulted in decreased viability only after exposure to high MOI, whereas exposure to *jin-3* resulted in reduced viability at lower MOI.

R124 reoviruses enter cancer cells via its high-affinity receptor junction adhesion molecule A (JAM-A),^44^^,^^49^^,^^50^ while the spontaneous mutant *jin-3* virus has a broader tropism due to its ability to also infect cancer cells independently of JAM-A, i.e., by binding negatively charged cell surface sialic acids.^44^

As expected, the expression levels of the canonical viral entry receptor JAM-A in tested cancer cell lines were in line with the ability of the reovirus variants to infect and/or replicate in the tumor cells (Figures 1E and S1G).

Upon challenge with R124 and *jin-3,* a dose-dependent increase in viral copy number was detected in 3D cultures of RT-112 cells (Figure S1H), coinciding with reduced viability of the treated tumoroids after 3 days (Figure S1I).

**Figure 2:**
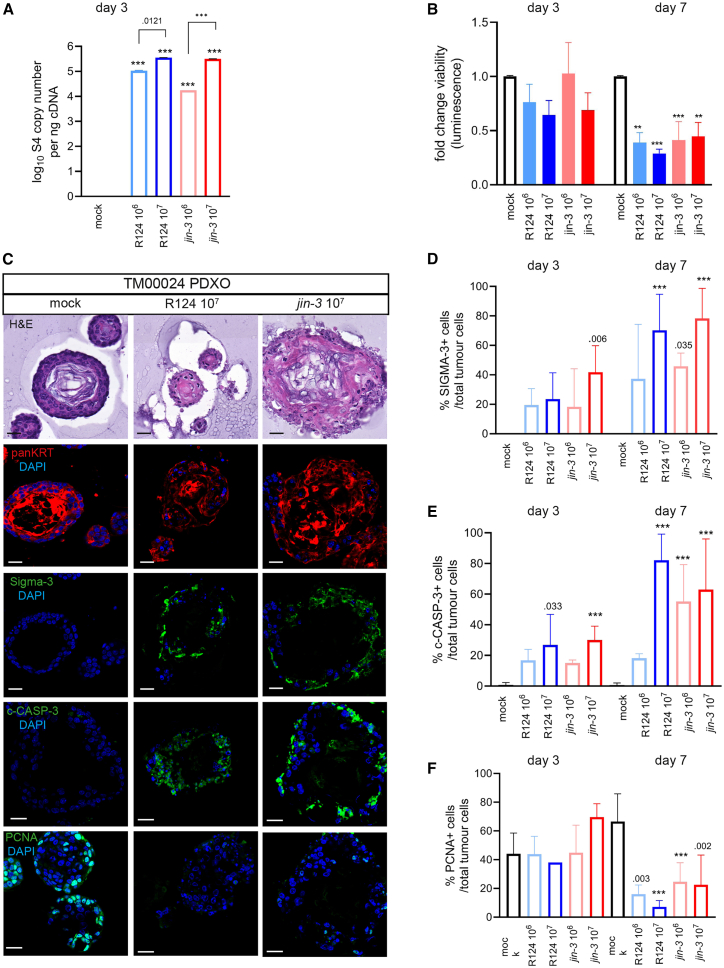
Comparison of the oncolytic effects of reovirus mutant *jin-3* versus wild-type reovirus R124 in 3D cultures *in vitro* TM00024 bladder PDXOs were exposed to the indicated plaque-forming units (PFUs) of reoviruses for 3 or 7 days. (A) Mean (SD) viral copy number of TM00024 PDXOs treated for 3 days with the indicated PFUs of the reoviruses. Two-way ANOVA followed by Tukey’s post hoc comparison (*n* = 3) ∗∗∗*p* < .001. (B–F) Viability (mean [SD] was measured using cell titer glo 3D after days 3 and 7 of reovirus exposure. *N* = 3 (3 replicates). Two-way ANOVA followed by Tukey’s post hoc comparison. ∗∗∗*p* < .001. (C) Confocal images of PDXOs (day 7) stained for respectively H&E, panKRT (red); Sigma-3, c-CASP3, or PCNA (green); and DAPI (blue). Scale bars, 20 μm. Cells expressing the respective proteins Sigma-3 (D), c-CASP-3 (E), and nuclear PCNA (F) were counted with ImageJ and divided by the amount of panKRT+_DAPI+ cells. At least five fields were scored per technical replicate. Mean (SD) of *N* = 3 (3 replicates). ∗∗∗*p* < .001, asterisks indicate reovirus infection versus mock same day. Two-way ANOVA followed by Tukey’s post hoc comparison.

In organoids cultured from the patient-derived muscle-invasive bladder cancer xenograft model PDX TM00024, R124 and *jin-3* showed dose-dependent infection and replication (Figure 2A).^51^ Furthermore, a significant reduction in cancer cell viability was observed after reovirus exposure for 7 days (Figure 2B). Viral protein (Sigma-3) was detected in the outer cell layers of the PDXOs, indicating active viral infection and replication after 3 days (Figures 2D and S2A).^52^^,^^53^ Moreover, an increased number of c-CASP-3-positive cells was measured (Figures 2E and S2A). Further increase of the Sigma-3- and c-CASP-3-positive cells was observed after 7 days (Figures 2C–2E and S2B for dose range), coinciding with a decrease in cell proliferation (nuclear PCNA; Figures 2C, 2F, and S2B) and loss of structural integrity of the PDXOs (Figures 2C and S2B). No significant differences were observed between the viruses.

**Figure 3:**
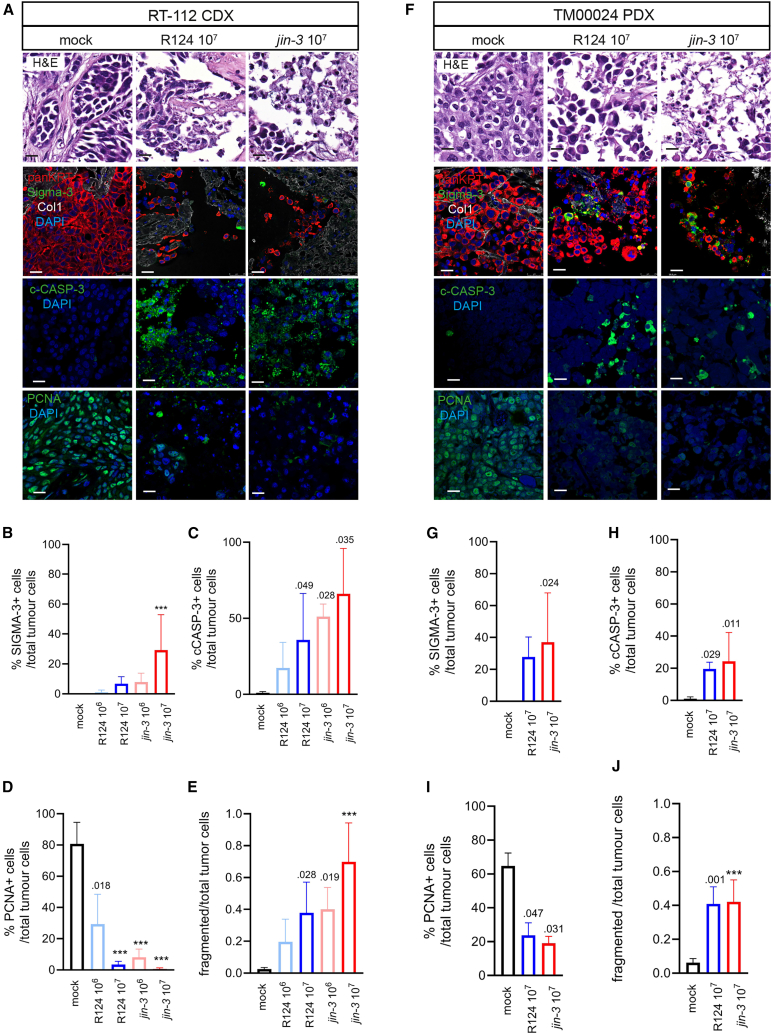
Comparison of *jin-3* and R124 reovirus infection in *ex vivo* cultured tumor tissue Explanted tissue slices from either RT-112 CDX (A–E) or TM00024 PDX (F–J) were exposed to the indicated PFU of R124 or *jin-3* reovirus for 3 days. Tissues were stained for H&E, panKRT (red); Sigma-3, c-CASP3 or PCNA (green), type I collagen (white), and DAPI (blue). Representative confocal images are shown. Scale bars, 20 μm. Cells expressing the respective proteins were counted with ImageJ and divided by the number of panKRT+_DAPI+ cells. At least four fields were scored per technical replicate. Mean (SD) of *N* = 2 (4 replicates). The ratio fragmented tumor cells was measured by the number of fragmented cells/total tumor cells (E and J). ∗∗∗*p* < .001, reovirus infection versus mock. Mean (SD), *n* = 2 (4 replicates). Two-way ANOVA followed by Tukey’s post hoc comparison.

Subsequently, viral infection and replication was determined in tumor tissue slices derived from cell-line-derived xenograft (CDX) and patient-derived xenograft (PDX) models after reovirus exposure. Upon exposure of tumor tissue slices of RT-112 bladder cancer cells^54^ to R124 and *jin-3 ex vivo* for 3 days, the viruses were capable of infecting the cancer cells and replicated in viral factories in the cell (Figures 3A and 3B). Furthermore, apoptosis was stimulated upon reovirus exposure as assessed by a relative increase of c-CASP-3-positive cells coinciding with a decline in tumor cell proliferation (Figures 3C–3E and S3A for dose range). In another CDX model J82 (Figure S4), similar results were obtained. Moreover, in *ex vivo* cultured tumor slices obtained from the PDX model TM00024, which displayed cell membranous JAM-A protein expression (Figure S3C), reovirus exposure also induced increased apoptosis and impaired tumor cell proliferation (Figures 3F–3J and S3B). No significant changes were observed between the two viruses.

**Figure 4:**
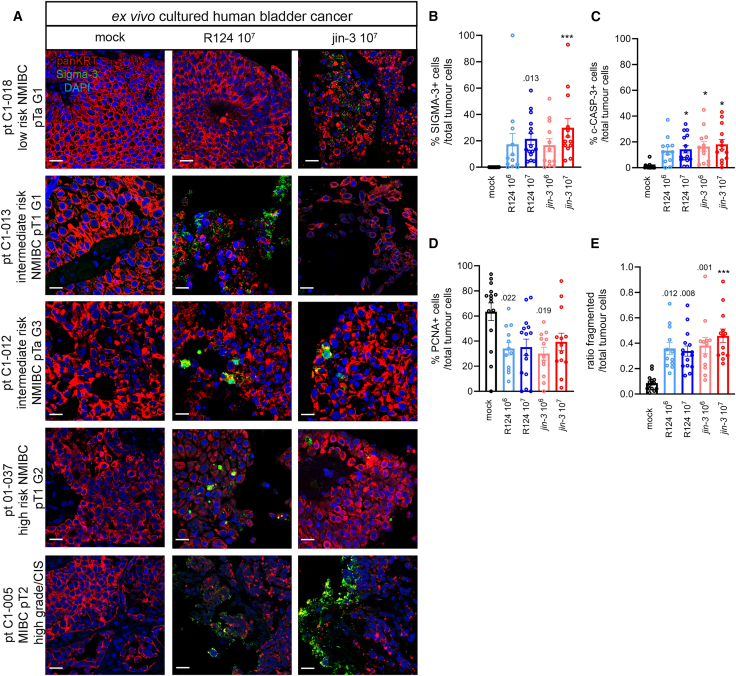
Comparison of *jin-3* and R124 reovirus infection in *ex vivo* cultured tumor tissue slices from human bladder cancer patients Explanted tissue slices from patients (*n* = 15) diagnosed with bladder cancer were exposed to either mock, R124, or *jin-3* reovirus for 3 days and stained for viral protein, c-CASP-3, PCNA (proliferation), and integrity of the cell (nuclear DAPI and panKRT tumor marker). (A–E) Representative confocal images of the mock and 10^7^ PFU condition of both viruses from five patients ranging from low-risk NMIBC to MIBC (viral protein [green, Sigma-3], tumor markers [red, panKRT], and nuclei [DAPI, blue]. Scale bars, 20 μm). Cells expressing the respective proteins [(B) Sigma-3, (C) c-CASP3, or (D) nuclear PCNA] were counted with ImageJ and divided by the number of panKRT+_DAPI+ cells. At least four fields were scored per technical replicate. Mean (SD) (3–4 replicates) with each dot representing one bladder cancer patient. The ratio of fragmented tumor cells was measured by the number of fragmented cells/total tumor cells (E). ∗∗∗*p* < 0.001, reovirus infection versus mock, one-way ANOVA followed by Tukey’ post hoc comparison.

Consistent with earlier findings,^47^ JAM-A expression was upregulated in bladder cancer compared to normal bladder (TCGA-BLCA database), although low levels of JAM-A are observed in 103/412 samples (Figures S1E and S1F). Next, tumor tissue slices were generated from freshly obtained tissues from patients diagnosed with various stages of bladder cancer (*n* = 15; Table 1). Tissue slices were exposed to mock, R124, or *jin-3* reoviruses for 3 days (Figures 4 and S5). Immunolocalization of JAM-A was determined in the patient tissues, and membranous localization was observed in all the patients, with one patient displaying lower levels of staining (pt C1-005; Figure S5B). Explanted tumor tissue was stained for c-CASP-3, PCNA (proliferation) and integrity of the cells (H&E staining and panKRT/DAPI staining). In the reovirus-treated patient-derived *ex vivo* cultured tumor explants, interpatient heterogeneity in Sigma-3 protein levels was observed, with Sigma-3 expression found in regions in multiple tissue slices per patient (Figures 4A and 4B). Exposure to reoviruses resulted in increased number of c-CASP-3-positive cells (Figures 4C and S5C), whereas the number of proliferating tumor cells was reduced (Figures 4D and S5C) as well as the integrity of the cells as indicated by the H&E staining and the increased ratio of fragmented cells (Figures 4E and S5A–S5C).

**Table 1:** Patient information: clinical histopathological characteristics of the patients included. Disease stage and grade at time of sampling of the tumor material

Patient identifier	Disease	Risk group	^55^Disease stage	Disease grade	Treatment history	Age	Gender	CIS
C1-005	MIBC	–	pT2	high-grade CIS	BCG bladder instillations	70	male	yes
01–032	MIBC	–	pT2	G3	unknown	69	male	no
C1-015	NMIBC	very high	pT1	focal G3	none	61	male	no
01–037	NMIBC	high	pT1	G2	none	82	female	no
C1-020	NMIBC	high	pTa/pT1	G3	none	76	male	no
C1-013	NMIBC	intermediate	pT1	low grade	none	87	male	no
C1-012	NMIBC	intermediate	pTa	G3	none	77	male	no
01–034	NMIBC	intermediate	pTa	G2	none	80	male	no
C1-008	NMIBC	intermediate	pT1	G1	none	62	male	no
C1-022	NMIBC	intermediate	pTa	G1	none	79	male	no
C1-047	NMIBC	intermediate	pTa	G1	none	64	male	no
C1-086	NMIBC	low	pTa	G1	none	67	male	no
C1-088	NMIBC	low	pTa	G1	none	73	male	no
C1-018	NMIBC	low	pTa	G1	none	67	male	no
C1-014	NMIBC	low	pTa	low grade	none	70	female	No

pTa, p(pathological) non-invasive papillary carcinoma; pT1, tumor invades subepithelial connective tissue; pT2, tumor invades muscle. Grading was scored using the WHO grading 1973 (G1: well differentiated; G2: moderately differentiated; G3: poorly differentiated) or WHO 2004 (low/high grade) MIBC, muscle-invasive bladder carcinoma; NMIBC, non-muscle invasive bladder carcinoma; CIS, carcinoma *in situ*; BCG, Bacillus Calmette-Guerin.

### Reovirus-induced immune activation

**Figure 5:**
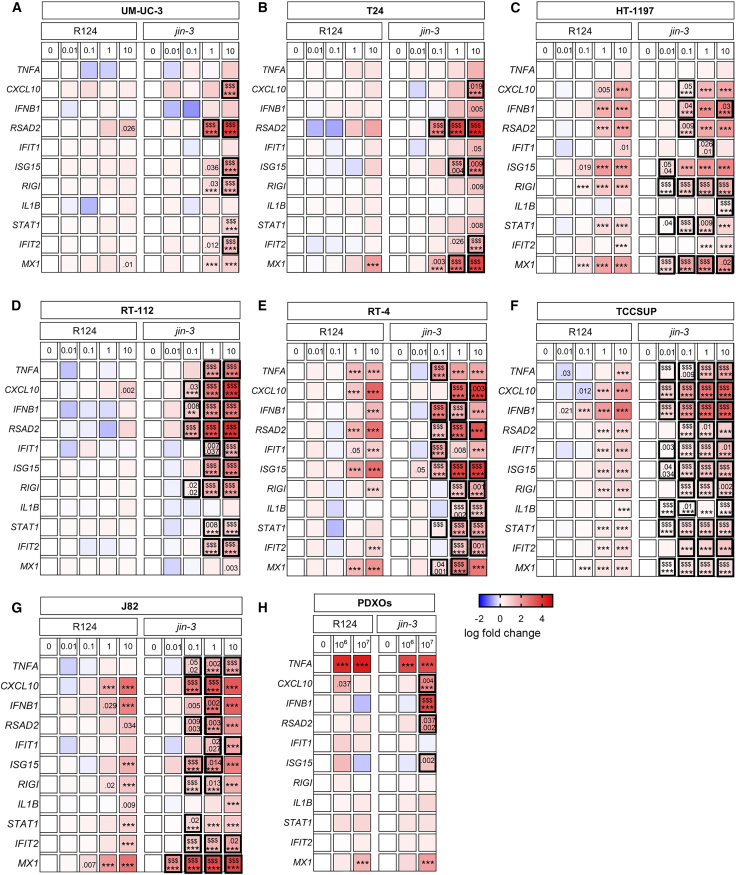
Comparison of immune modulation induced by *jin-3* and R124 reovirus treatment of bladder cancer cell lines Heat maps of various inflammatory cytokines and interferon-stimulated genes (log fold change mRNA expression [2^−ΔΔCt^] vs. mock treated cells) in bladder cancer cell lines UM-UC-3 (A), T24 (B), HT-1197 (C), RT-112 (D), RT-4 (E), TCCSUP (F), J82 (G), and PDXOs (H) exposed to a range of either R124 or *jin-3* reoviruses after respectively 24 h (cell lines) and 3 days (PDXOs). *p* values are depicted (vs. mock; when 2 depicted upper *p* value is R124 vs. *jin-3*) ∗∗∗*p* < .001, $$$ *p* < .001, asterisks indicate mock versus reovirus infection, and dollar signs indicate R124 versus *jin-3.*

Inflammatory cytokines, type I interferons (IFNs), and IFN-stimulated genes (ISGs) are of key significance in oncolytic virotherapy that largely relies on the stimulation of anti-tumor and anti-viral immune cell activation.^55^^,^^56^^,^^57^ Exposure to R124 and *jin-3* reovirus resulted in a significant and dose-dependent upregulation of IFN-β gene expression (*IFNB1*) after 24 h in multiple bladder cancer cell lines (Figures 5A–5G). *jin-3* treatment resulted in a significantly stronger upregulation in *IFNB1* (6/7) compared to R124 (4/7) in all the tested cell lines (Figures 5C–5G), except for UM-UC-3 and T24 (Figures 5A and 5B). Moreover, exposure to *jin-3* led to significantly stronger induction of ISGs (i.e., *ISG15*, *IFIT1*, *IFIT2*, *RSAD2*, and *MX1*) in most of the tested cell lines compared to R124 (Figure 5). Exposure to *jin-3* led to a superior and significant expression of inflammatory cytokines *CXCL10* (in 7/7 cell lines), *TNF* (4/7), *and IL1B* (2/7) compared with R124 (Figure 5). Moreover, the cytosolic RNA sensor *RIG-I* was significantly upregulated after *jin-3* exposure compared to R124 in 6/7 cell lines (Figure 5). In the TM00024 PDXOs, exposure to *jin-3* reovirus resulted in significant upregulation of *TNF, CXCL10*, *IFNB1*, and *RSAD2* gene expression, whereas R124 reovirus only induced *TNF* gene expression (Figure 5H).

**Figure 6:**
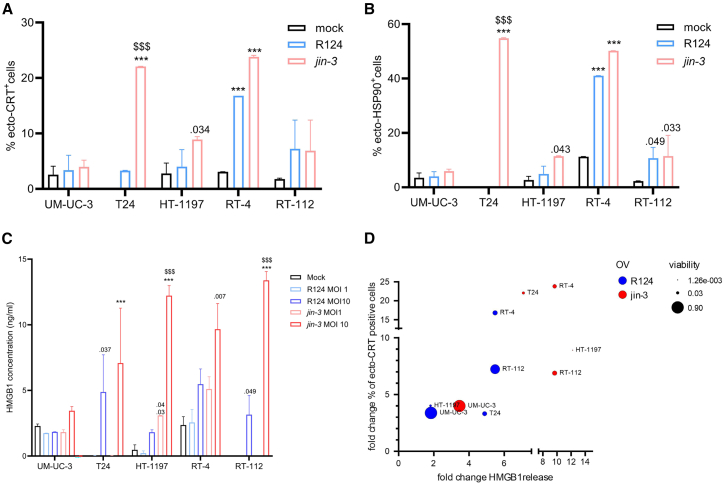
Comparison of immunogenic cell death induced by *jin-3* and R124 reovirus treatment of bladder cancer cell lines (A–C) Cell lines were treated with oncolytic viruses for 48 h at an MOI of 10, and the DAMPs ecto-calreticulin (A) and ecto-HSP90 (B) were measured using flow cytometry. Secreted HMGB1 protein was measured with an ELISA after 48 h of treatment with a dose range of oncolytic viruses (C). ∗∗∗*p* < 0.001, $$$ *p* < 0.001, asterisks indicate reovirus infection versus mock same day, and dollar signs indicate R124 vs. *jin-3.* Mean (SD), *N* = 3 (2 replicates). Two-way ANOVA followed by Tukey’s posthoc comparison. (D) Correlation graph of the HMGB1 release (*x* axis), percentage of viable cells (size of the dots), and fold change of ecto-CRT (*y* axis)-positive cells at MOI 10 of the indicated virus.

Cell surface expression of the DAMPs ecto-calreticulin and ecto-HSP90 was significantly increased upon exposure to reoviruses (Figures 6A and 6B). Strikingly, *jin-3* caused a more pronounced increase in HMGB1 release, a well-established marker of immunogenic cell death^42^ (Figure 6C). Correlation between these markers at an MOI of 10 is depicted in Figure 6D.

**Figure 7:**
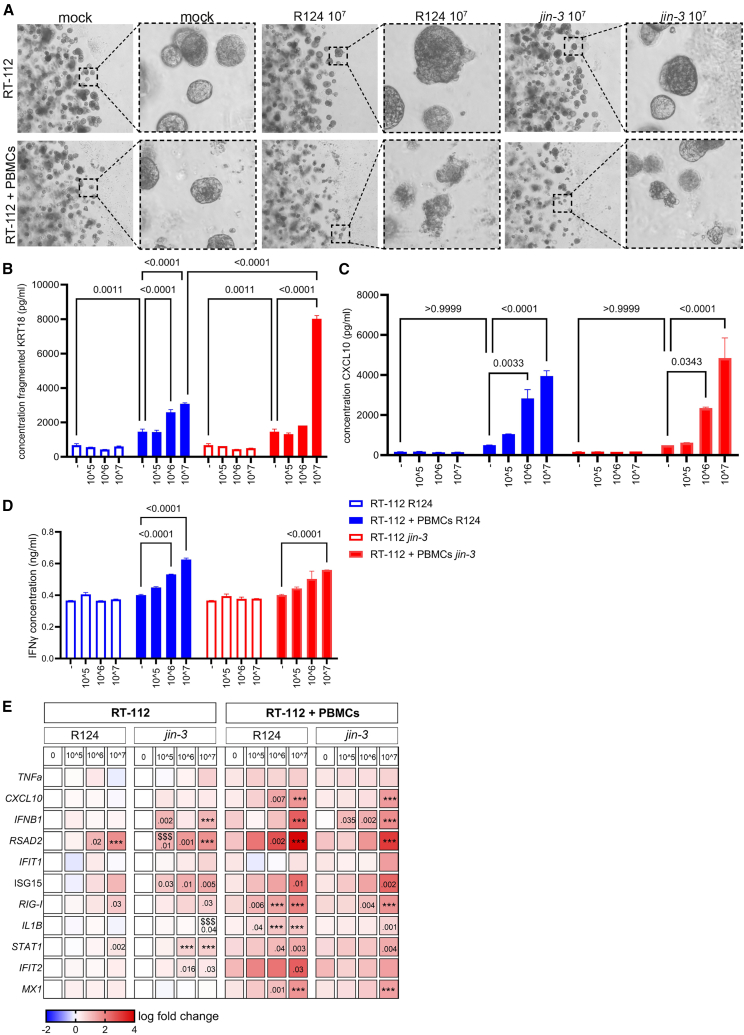
Activation of PBMCs in RT-112 co-cultures (A) Brightfield images of RT-112 bladder cancer cells that were cultured in 60% Matrigel and allowed to form 3D structures for approximately 3 days. Subsequently, PBMCs were added at an effector: target ratio of 20:1 in absence or presence of either R124 or *jin-3* reovirus at the indicated PFU for an additional 3 days. (B) Fragmented KRT18 was determined as an outcome measure for tumor cell killing 3 days after OV exposure. (C–E) CXCL10 levels and (D) IFN-γ levels were measured 3 days after OV exposure. (*n* = 3, 2 replicates). Mean (SD). Two-way ANOVA with Tukey’s post hoc comparison. (E) Heatmap of various inflammatory cytokines and interferon-stimulated genes (log fold change mRNA expression [2^−ΔΔCt^] vs. mock treated cells) in 3D cultured RT-112 cells or RT-112 cells co-cultured with PBMCs exposed to either R124 or *jin-3* reoviruses. Mean (SD). *p* values are depicted (vs. mock; when two depicted upper *p* value is R124 vs. *jin-3*) ∗∗∗*p* < .001, $$$ *p* < .001, asterisks indicate mock versus reovirus infection and dollar signs R124 versus *jin-3.*, *n* = 2 (2 replicates). Two-way ANOVA followed by Tukey’s post hoc comparison.

Subsequently, co-culturing of 3D tumoroids of human RT-112 cells with partially HLA-matched peripheral blood mononuclear cells (PBMCs) led to increased tumoroid killing (Figure S7A). This was accompanied with significantly increased release of fragmented cytokeratin-18 (KRT18; Figure S7B), CXCL10 (Figure S7C), and IFN-γ (Figure S7D). Addition of reoviruses to the 3D cultures resulted in expression of viral transcripts in the reovirus-treated RT-112 cells, with a lower copy number when the 3D cultures were co-cultured with PBMCs (Figure S7E). Reovirus treatment in the co-cultures resulted in augmented tumoroid killing compared to the mock-treated conditions, with *jin-3* displaying significantly more pronounced effects (Figures 7A and 7B). Moreover, chemo- and cytokine production (CXCL10 and IFN-γ) and mRNA expression of type I IFN response genes (e.g., *IFNB1*, *RSAD2*, *MX1*, *IFIT2*, and *ISG15* mRNA levels) was significantly enhanced upon reovirus treatment in these cultures (Figures 7C–7E and S7F–S7P).

## Discussion

In this study, we evaluated the direct oncolytic and immunomodulatory effects of reoviruses R124 and *jin-3* in preclinical human bladder cancer models.

We observed that reoviruses induce potent oncolytic effects in flatbottom 2D and 3D cultures of human bladder cancer. Despite the differences in oncolytic responses, which are likely to be due to well-documented clinical inter-tumoral heterogeneity in bladder cancer,^58^^,^^59^ we observed viral infection upon reovirus administration in most human bladder cancer cells. Moreover, we detected viral infection and replication in near-patient *ex vivo* cultured bladder cancer tissue slices derived from CDX and PDX models and primary bladder cancer patient material.

Besides oncolytic properties, oncolytic viruses can induce both anti-viral immunity and anti-tumor immune responses, which is required for long-lasting and systemic tumor control.^60^ Effective oncolytic virotherapy relies on the production of pro-inflammatory cytokines and type I IFNs and the induction of interferon-stimulated genes (ISGs). Pro-inflammatory chemokines and cytokines are released and attract lymphocytes toward the tumor.^60^^,^^61^^,^^62^ These anti-viral immunological events could turn immunological “cold” tumors into “hot” tumors. Our study demonstrated that *jin-3* reovirus led to elevated expression of ISG and inflammatory cytokine production by infected bladder cancer cells compared to R124 wild-type reovirus. This is further substantiated by the augmented release of immunogenic cell death markers HMGB1 and DAMPs (i.e., ectopic cell surface expression of calreticulin and HSP90 after exposure to *jin-3* reovirus). Co-culturing of bladder tumoroids with PBMCs resulted in a significant, dose-dependent increase in tumor cell death and elevated production of CXCL10 and IFN-γ after reovirus exposure. This is consistent with findings in a colorectal cancer co-culture system in which autologous PBMCs acquire tumor-targeting capabilities, which correlated with clinical responses to checkpoint inhibitors.^63^

Overall, our preclinical results support the notion that infection of the human bladder cancer cells with reoviruses, especially *jin-3*, induces oncolysis and may contribute to a switch from an immunologically “cold’’ toward an immunological “hot’’ inflammatory phenotype. Additionally, *jin-3* has an extended tropism compared to the wild-type reovirus because these viruses can infect cells independently of JAM-A expression on the tumor cell surface. These results are in line with previous reports in prostate cancer cells showing that intra-tumoral injection of wild-type reovirus induced pro-inflammatory cytokines production.^45^^,^^64^

Intravesical administration of reovirus in an orthotopic model of superficial bladder cancer revealed minor side effects of therapy with significantly higher tumor-free survival compared to BCG treatment.^65^ Moreover, in a syngeneic mouse model, intravesical therapy of reovirus in combination with an anti-programmed cell death protein 1 (PD-1) antibody improved survival and induced a more immune-active (hot) tumor microenvironment.^66^

Previous clinical studies have also demonstrated the potential of oncolytic viruses in cancer therapy, highlighting their ability to target and destroy cancer cells selectively. For example, T-VEC is a Food and Drug Administration (FDA)-approved herpes-simplex-virus-based therapy for advanced melanoma.^67^ Other oncolytic viruses including vesicular stomatitis virus, adenovirus, and vaccinia virus have been studied in multiple cancer types, including bladder cancer.^68^^,^^69^^,^^70^^,^^71^^,^^72^^,^^73^^,^^74^^,^^75^^,^^76^

Some oncolytic viruses, for example, ICAM-1 targeted Coxsackievirus A21 (CVA21) and vaccinia virus, have shown acceptable safety profiles and proof of viral targeting and replication in the bladder tumor cells in clinical trials.^68^^,^^71^^,^^77^ In addition, in early stage bladder cancer, oncolytic adenoviruses have been efficacious in BCG-refractory patients.^78^^,^^79^^,^^80^^,^^81^^,^^82^^,^^83^^,^^84^^,^^85^

Importantly, clinical trials have revealed that pelareorep, also known as Reolysin, an isolate of wild-type T3 Dearing reovirus, can be safely administered to cancer patients, including patients with advanced cancer such as multiple melanoma, prostate, and esophagus cancer, with relatively few adverse effects.^43^^,^^49^^,^^86^^,^^87^^,^^88^^,^^89^ However, the clinical benefit of wild-type reovirus administration is limited, and phase II studies have revealed no survival benefit.^90^ No clinical trials with reovirus have been reported for bladder cancer yet. Clinical benefit may be enhanced by using our reovirus mutant *jin-3* with an expanded tropism, beyond the canonical reovirus entry receptor JAM-A.^44^

Due to the significant variability between and within tumors—including genetic mutations and differences in the tumor microenvironment—it is essential to develop diverse and improved oncolytic virotherapy strategies using multiple types of oncolytic viruses.

Matching the most effective oncolytic virus(es) to an individual patient is key for virotherapy to become more effective. Stratification of patients who are more likely to respond to a specific oncolytic virus is required but—to date—not yet accomplished. If proven, this personalized approach will enhance treatment outcomes by matching the best candidate virus to an individual tumor based on its unique biological characteristics.

Accumulating evidence shows that oncolytic virotherapy, when used as a monotherapy, is unlikely to fully overcome the immunosuppressive tumor microenvironment in a clinical setting. Several clinical studies are with oncolytic reovirus in combination with immune checkpoint inhibition in various cancers including pancreatic adenocarcinoma, metastatic non-small lung cancer, multiple myeloma, and metastatic colorectal cancer.^91^^,^^92^^,^^93^^,^^94^^,^^95^^,^^96^^,^^97^ For example, in the GOBLET multi-cohort phase 1/2 study in pancreatic and anal cancer, intravenous pelareorep, in combination with the PD-L1 inhibitor atezolizumab, displayed promising efficacy (respectively 62% and 37.5% objective response rate for metastatic pancreatic and anal cancer).^98^ Furthermore, combination of pelareorep with the PD-1 inhibitor pembrolizumab modestly promoted anti-tumor activity in advanced pancreatic cancer.^99^

To better understand the interactions between oncolytic viruses and the immune system, tumor-immune cell co-culture systems—as the ones described here—can be exploited. By incorporating both tumor cells and relevant immune cell populations (e.g., T cell populations and dendritic cells), these systems can be used to assess how different OVs modulate immune cell activation, suppression, or recruitment, to monitor cytokine and chemokine production in response to viral infection and to evaluate the potential for synergy or antagonism when combining oncolytic viruses with current immunotherapy (immune checkpoint inhibition or cell-based immunotherapy). Correct timing, optimal oncolytic virus (hence the repertoire of OVs), optimal dosing, and treatment combinations are key.

Collectively, our findings underscore the oncolytic potential of reoviruses in diverse preclinical models of human bladder cancer. Notably, the mutant reovirus *jin-3* exhibits a more robust immunomodulatory response in preclinical bladder cancer models compared to the wild-type reovirus R124. This promising result positions *jin-3* as a compelling candidate for future clinical translation.

## Material and methods

### Reovirus production

Wild-type T3D reovirus strain R124 was plaque-purified from the wild-type reovirus T3D (ATCC) on HER911 cells.^44^^,^^100^ Reovirus mutant *jin-3* was isolated from JAM-A-deficient U118MG cells after passaging of the wild-type T3D strain R124.^44^ Both R124 and *jin-3* reoviruses were propagated, purified, and titrated on human HER911 cells as previously described.^44^

### Preclinical models and culture conditions

*Cell lines as monolayers*: a panel of human bladder cancer cell lines was cultured in monolayers with the respective culture medium (Table S1).

*Three-dimensional bladder cancer tumoroids or patient-derived xenograft (PDX) organoids (PDXOs)* were cultured from cells isolated from subcutaneously growing PDX TM00024 tumors^51^ (Jackson Laboratory; ethical permit AVD16605) or from the RT-112 and J82 cell lines as described previously.^52^

*Three-dimensional tumor-immune cell co-cultures*: 10,000 RT-112 cells were seeded in 40 μL domes in 60% Matrigel. After 3 days, partially HLA-matched PBMCs (Sanquin NVT0631.01) were added at a 20:1 effector target ratio to the 3D cultures. After 3 days, conditioned medium was collected, and RNA was isolated as described below.

*Ex vivo cultured tissue explants* were sliced and cultured as previously described.^54^ Bladder cancer tissue was derived from either CDX, PDX, or directly from patient tumor material (Biobank Urology protocol B20.060, animal ethical protocol AVD16605; Table 1). Multiple tissue slices were used per condition and cultured for 4 days.

### Viability assays

*Monolayer experiments:* 1,500 cells per well were seeded in a 96-well plates. After 24 h, these cells were exposed to a range of either R124 or *jin-3* (MOI 0–100 plaque-forming units [PFUs]/cell). Cells were incubated for 72 or 144 h, after which a CellTiter 96 AQueous One Solution Cell Proliferation Assay (Promega) was performed according to manufacturer’s protocol. After 2 h of incubation time, optical density was measured at 490 nm using SpectraMax iD3 plate reader to assess cell viability.^45^

The 3D tumoroids or PDXOs were exposed to a range of R124 and *jin-3* reoviruses. After 3–7 days, viability was determined using the Cell Titer Glo assay, according to the manufacturer’s protocol (Promega).^45^

### Flow cytometry

Four hundred thousand cells/well were seeded in a 6-well plate. Each cell line was exposed to either R124 or *jin-3* (mock solution only, MOI 10) and incubated for 48 h. Subsequently, levels of viral protein expression (Sigma-3), apoptosis (cleaved caspase-3 [c-CASP-3]), and protein expression of several DAMPs (cell surface expression of calreticulin and HSP90) for each cell line/virus combination were acquired using LSR Fortessa X-20 Cell Analyzer and analyzed using FlowJo software (Table S1).

### Histology and immunofluorescence

H&E and immunofluorescent stainings were executed as previously described.^54^ H&E and immunofluorescent stainings were visualized using the Pannoramic MIDI slide scanner (3DHISTECH). All fluorescent stainings were visualized by confocal microscopy (63× magnification, resolution 1,024 × 1,024) (Leica SP8). At least four fields of the fluorescent staining were scored per TS by two independent reviewers. The average of these 4 fields was shown for each of the technical replicates. Cells expressing the respective proteins were counted with ImageJ and divided by the number of pancytokeratin-positive and DAPI-positive (panKRT+_DAPI+) cells (i.e., intact tumor cells with intact nuclei). The ratio of fragmented tumor cells was measured by the number of fragmented cells divided by the number of total tumor cells.

### Real-time quantitative polymerase chain reaction

Cells were infected with the viruses and after the indicated time RNA was isolated according to the manufacturer’s protocol (Nucleospin RNA kit Macherey-Nagel). cDNA was generated by using random primers (Promega), and real-time qPCR was performed with GoTaq Mastermix (Promega) according to the manufacturer’s protocol in technical duplicates and biological triplicates (Promega). Gene expression was normalized to GAPDH. Primer sequences can be found in Table S1.

### ELISAs

Cells were exposed to R124 or *jin-3* with an MOI of 10. At the indicated time post exposure, HMGB1 (high mobility group box 1) release was measured by performing the Lumit HMGB1 Human/Mouse Immunoassay (Promega).

Three-dimensional (co-) cultures were exposed to R124 or *jin-3*. At the indicated time points post exposure, conditioned medium was collected, and either fragmented KRT18 (Abcam), IFN-γ (Abcam), or CXCL10 (Invitrogen) levels were measured as indicated by the manufacturers protocol.

#### Statistical analysis

Statistical analyses were performed by using GraphPad Prism 8.0. Two-way ANOVA was performed for viability and ELISA experiments followed by Tukey post hoc test for multiple comparison. For *ex vivo* cultures, sample size was calculated using proportional odds models as described.^101^ Multiple slices were scored per condition per patient (∗*p* < 0.05; ∗∗*p* < 0.01; ∗∗∗*p* < 0.001).

## Data and code availability

Data supporting the findings of this study are available within the paper and its supplemental information.

## Acknowledgments

This study was supported by the OAK Foundation (www.supportcasper.nl;STRATIVIR and SOAK18.01), the 10.13039/501100004622KWF Dutch Cancer Society (KWF15932), and a kind gift from the Franje Foundation.

## Author contributions

Conceptualization, R.H., G.P., and G.H.; data curation, A.F., M.M., and G.H.; formal analysis, A.F., M.M., L.H., D.W., and G.H.; funding acquisition, R.H., G.P., and G.H.; investigation, A.F., M.M., L.H., D.W., and G.H.; methodology, A.F., M.M., D.W., and G.H.; project administration, G.P. and G.H.; resources, R.H., D.W., R.F., and W.Z.; supervision, R.H., G.P., and G.H.; validation, A.F., M.M., G.P., and G.H.; visualization, A.F., M.M., and G.H.; writing—original draft, A.F. and G.H.; writing—review and editing, A.F., R.H., M.K., G.P., and G.H.

## Declaration of interests

The authors declare no competing interests.

## Supplemental information

Document S1. Figures S1–S7 and Table S1

Table S1. Key resources, critical equipment and software

Document S2. Article plus supplemental information
