# Supplementary material for: Improved oncolytic and immunostimulatory activity of the spontaneous jin-3 reovirus mutant in preclinical bladder cancer models
Source: Mol Ther Oncol. 2026 Jan 13;34(1):201128. doi: 10.1016/j.omton.2026.201128 (PMC12860623; doi:10.1016/j.omton.2026.201128)
Supplement: Document S1. Figures S1–S7 and Table S1 [file mmc1.pdf]

OMTON, Volume *34*

Supplemental information

Improved oncolytic and immunostimulatory activity of the spontaneous *jin-3* reovirus mutant in preclinical bladder cancer models

Arjanneke F. van de Merbel, Maaike H. van der Mark, Lobke C.M. Hensen, Diana J.M. van den Wollenberg, Rob C. Hoeben, Willemijn C.G. Zonneveld, Rob C.M. Pelger, Maxime T. M. Kummeling, Geertje van der Horst, and Gabri van der Pluijm

Figure S1

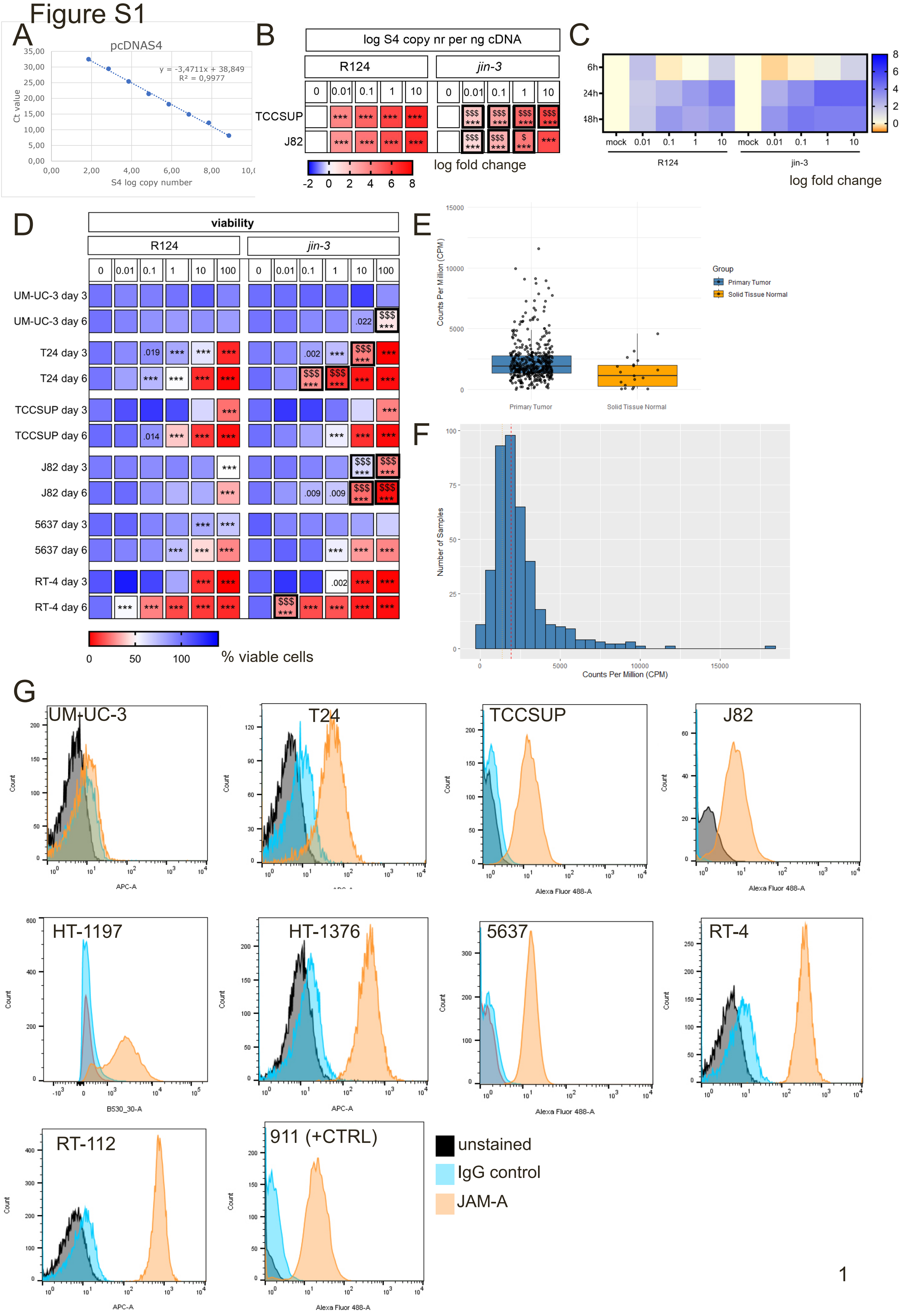

A
pcDNAS4
y = -3,4711x + 38,849
R² = 0,9977
Ct value
S4 log copy number
B
log S4 copy nr per ng cDNA
R124
jin-3
TCCSUP
J82
log fold change
C
6h
24h
48h
mock
R124
jin-3
log fold change
D
viability
R124
jin-3
UM-UC-3 day 3
UM-UC-3 day 6
T24 day 3
T24 day 6
TCCSUP day 3
TCCSUP day 6
J82 day 3
J82 day 6
5637 day 3
5637 day 6
RT-4 day 3
RT-4 day 6
% viable cells
E
Counts Per Million (CPM)
Group
Primary Tumor
Solid Tissue Normal
F
Number of Samples
Counts Per Million (CPM)
G
UM-UC-3
T24
TCCSUP
J82
HT-1197
HT-1376
5637
RT-4
RT-112
911 (+CTRL)
Count
APC-A
Alexa Fluor 488-A
B530_30-A
unstained
IgG control
JAM-A

Figure S1

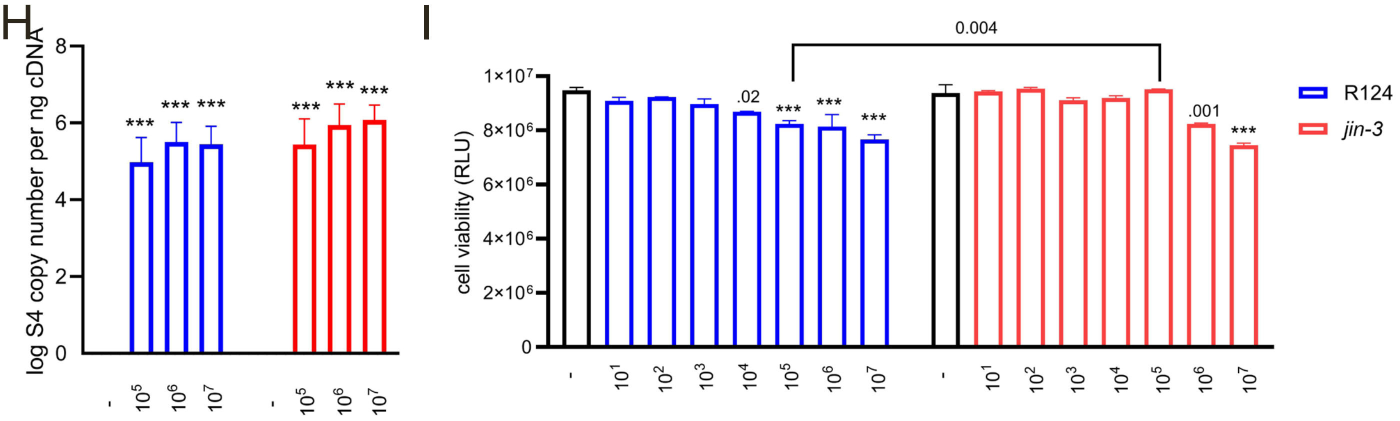

Figure S1: Comparison of the oncolytic effects of reovirus mutant *jin-3* versus wildtype reovirus R124 in bladder cancer cell lines *in vitro* 2D: viral copy numbers.
(A) Standard curve of pcDNA S4 for determination of viral copy number. (B) Heatmap of the viral load (log fold change S4Q mRNA expression vs mock treated cells) in bladder cancer cell lines TCCSUP and J82 exposed to a range of MOI (0.01-0.1-1-10) of either R124 or *jin-3* reoviruses after 24h. (C) Heatmap of the viral load (log fold change S4Q mRNA expression vs mock treated cells) in bladder cancer cell line RT-4 exposed to a range of MOI (0.01-0.1-1-10) of either R124 or *jin-3* reoviruses after respectively 6h, 24h or 48h. Mean (SD) and P values are depicted (vs mock; when 2 depicted the upper P value is comparison R124 vs *jin-3)* *** p<.001, $$$ p<.001, asterisks indicate mock versus reovirus infection, dollar signs R124 versus *jin-3*., N=2 (2 replicates). Two-way ANOVA followed by Tukey's posthoc comparison. (D) Heatmap of mean percentage of viable cells after exposure to a range of MOI of either R124 or *jin-3* for 6 days. N=3 (6 replicates). P values are depicted (vs mock; when 2 depicted upper P value is R124 vs *jin-3*) *** p<.001, $$$ p<.001, asterisks indicate mock versus reovirus infection, dollar signs R124 versus *jin-3*. MOI = multiplicity of infection. (E) Expression analysis of JAM-A/F11R in normal bladder vs primary bladder cancer (counts per million in TCGA-BLCA). (F) Distribution of the counts per million (CPM) of F11R (JAM-A) in TCGA-BLCA primary bladder tumors. (G) fluorescence intensity histograms of the indicated established cell lines stained for JAM-A. (H) viral load (log fold change S4Q mRNA expression vs mock treated cells) in 3D tumoroids of the bladder cancer cell line RT-112 exposed to a range of either R124 or *jin-3* reoviruses (10^5-10^7 pfu) after 3 days of exposure. N=2 (2 replicates). Two-way ANOVA followed by Tukey's posthoc comparison. (I) cell viability was measured using cell titer glo 3D for RT-112 bladder tumoroids after 3 days of treatment with a dose range of viruses.*** p<.001, asterisks indicate mock versus reovirus infection.

Figure S2

A

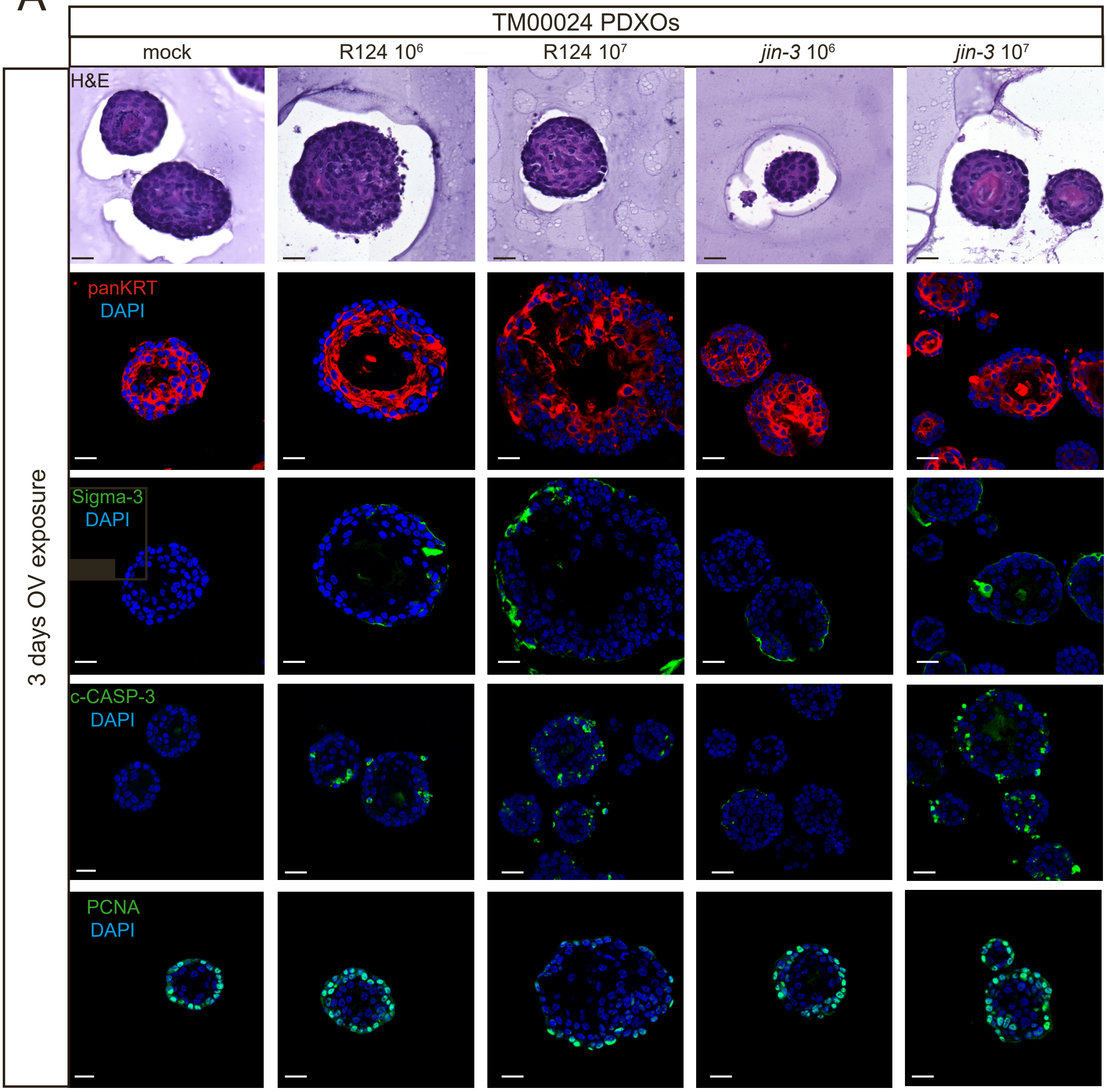

TM00024 PDXOs
mock
R124 10⁶
R124 10⁷
jin-3 10⁶
jin-3 10⁷
3 days OV exposure
H&E
panKRT
DAPI
Sigma-3
DAPI
c-CASP-3
DAPI
PCNA
DAPI

Figure S2

B

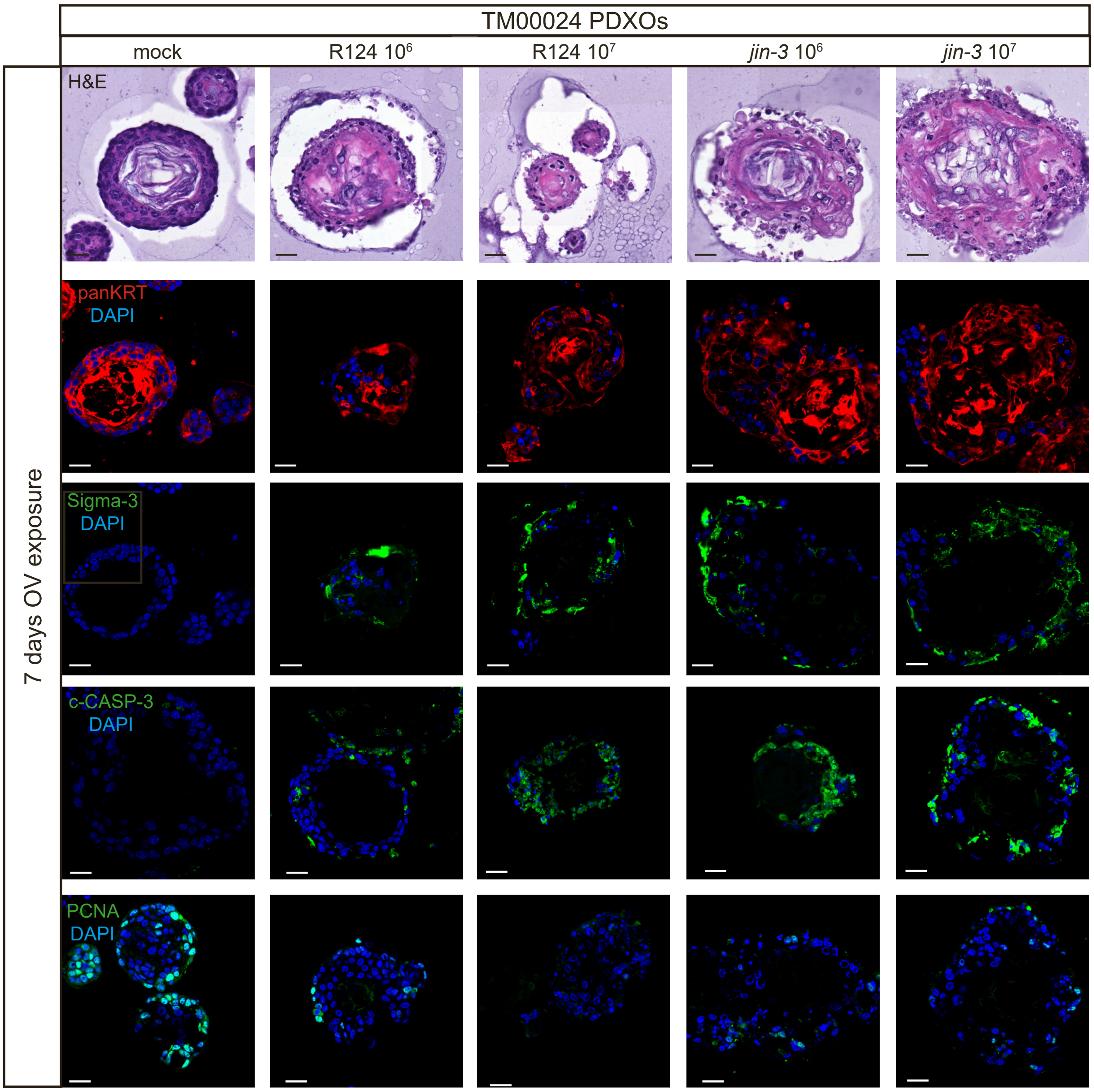

C

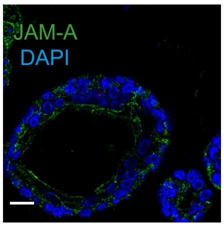

Figure S2 Comparison of the oncolytic effects of reovirus mutant *jin-3* versus wildtype reovirus R124 in PDXOs *in vitro*
TM00024 bladder PDXOs were treated with the indicated pfu of reoviruses for either 3 (A) or 7 days (B). Confocal images of PDXOs are shown stained for respectively H&E, panKRT (red); Sigma-3, c-CASP3 or PCNA (green) and DAPI (blue). Images of day 7 PDXOs treated with either mock,or 10^7 pfu concentrations of both viruses have been reused from figure 2C, that depicted only the highest dosage of virus. Scale bar 20 μm. (C) Confocal images of JAM-A expression (JAM-A in green, and DAPI in blue) in TM00024 PDXOs.

Figure S3

A

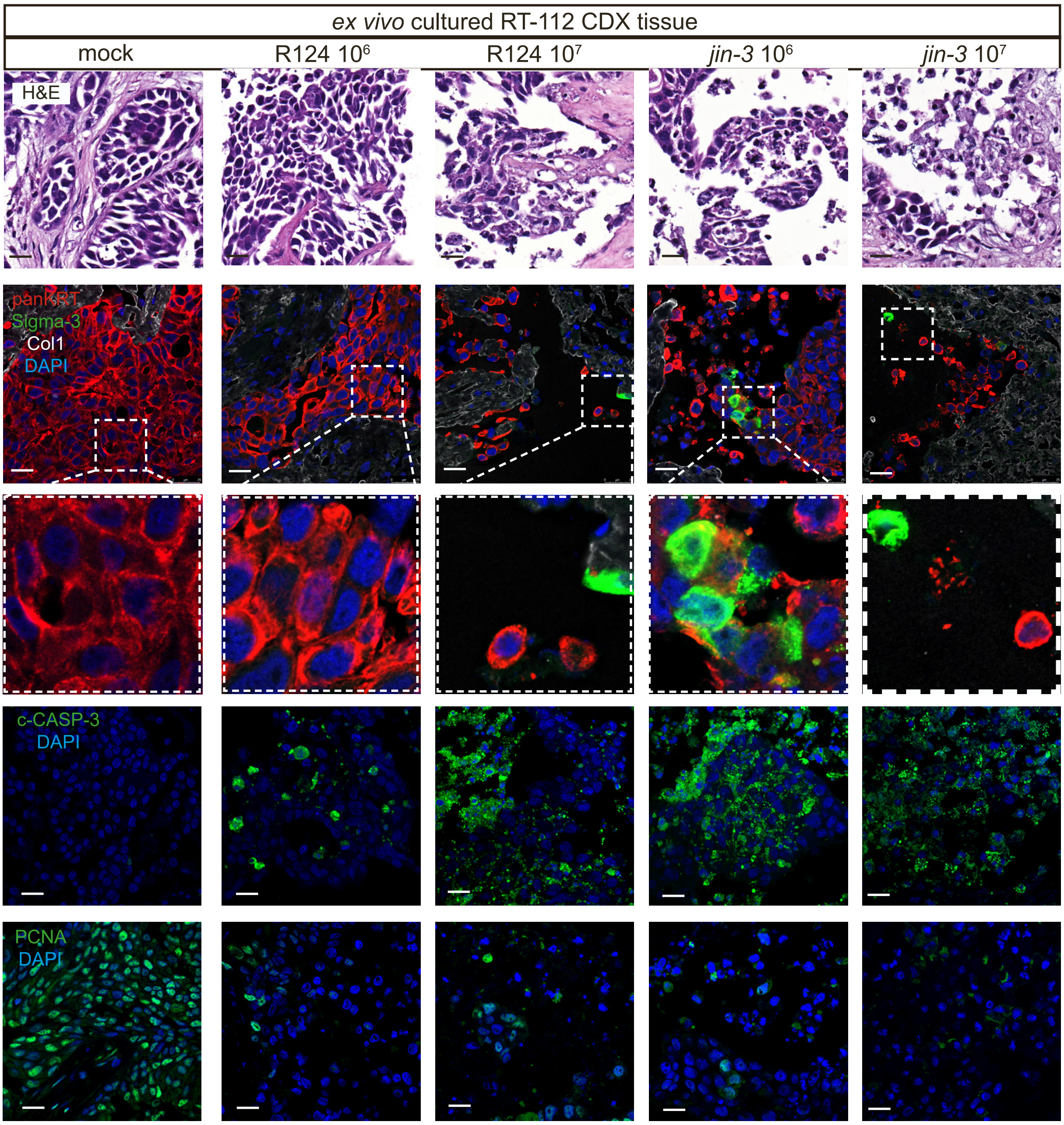

B

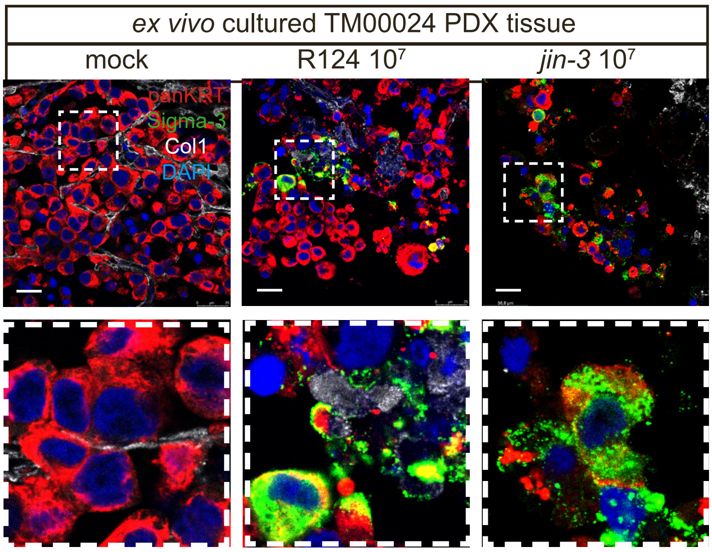

C

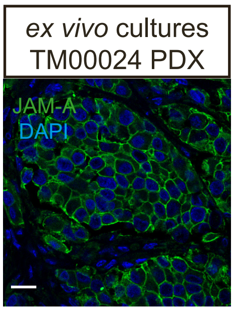

Figure S3 Comparison of the oncolytic effects of reovirus mutant *jin-3* versus wildtype reovirus R124 in *ex vivo* cultured bladder cancer tissue.

Explanted tissue slices from either (A) RT-112 CDX or (B) TM00024 PDX were exposed to the indicated pfu of R124 or *jin-3* reovirus for 3 days. Tissues were stained for HE, panKRT (red); Sigma-3, c-CASP3 or PCNA (green), collagen type I (white) and DAPI (blue) and representative confocal images are shown. Images of *ex vivo* cultured RT-112 CDX treated with either mock,or10^7 pfu concentrations of both viruses have been reused from figure 3A, that depicted only the highest dosage of virus. Images of *ex vivo* cultured TM00024 PDX have been reused from figure 3F. Scale bar 25 µm. (C) Confocal images of JAM-A expression (JAM-A in green, and DAPI in blue) in *ex vivo* cultured TM0024 tissue.

Figure S4

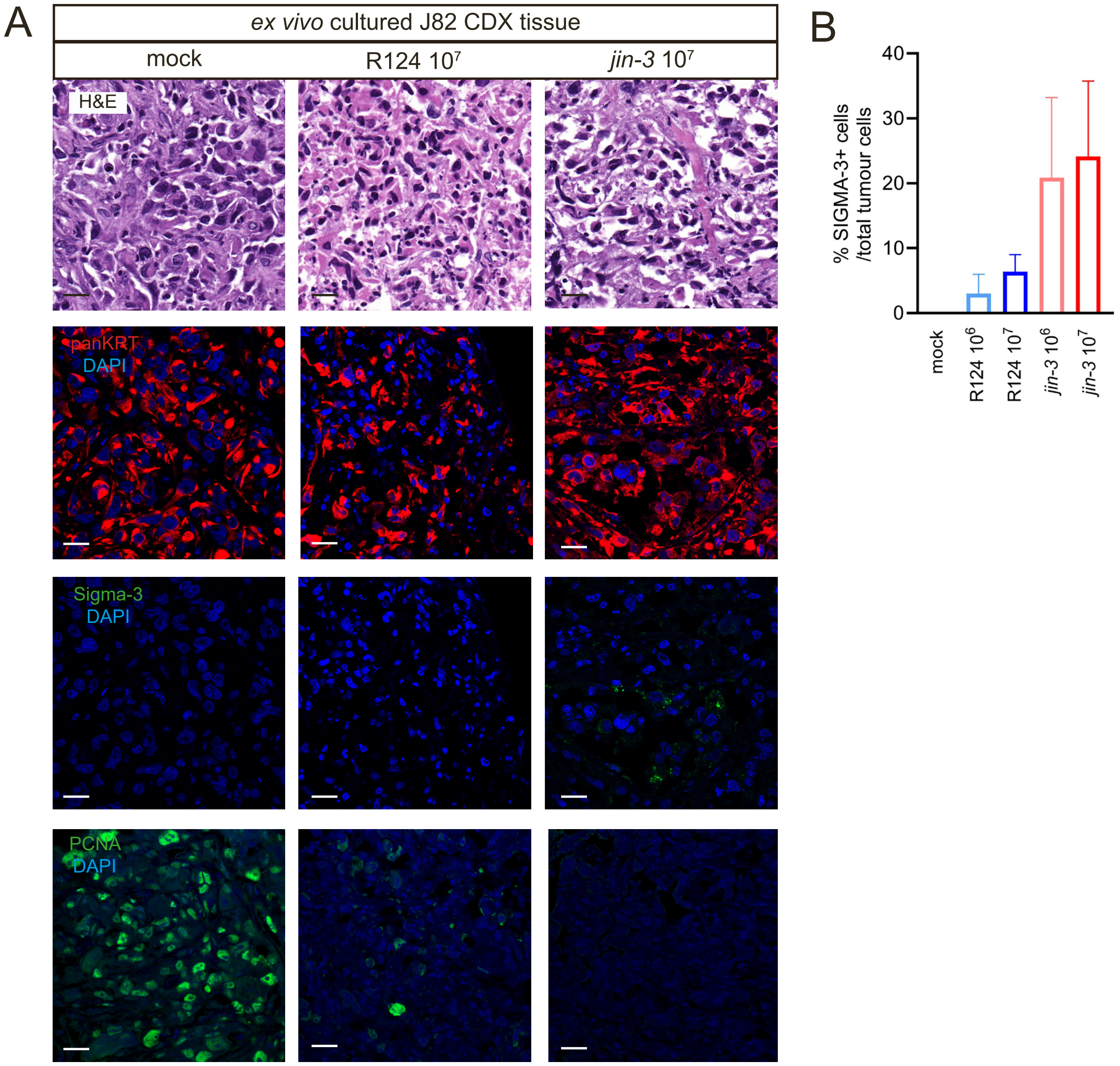

Figure S4 Comparison of *jin-3* and R124 reovirus infection in *ex vivo* cultured tumour tissue slices from cell-line derived xenograft J82

Explanted tissue slices from J82 CDX model were exposed to 10^6 or 10^7 pfu R124 or *jin-3* reovirus for 3 days. For each condition, multiple tissue slices were stained for H&E, panKRT (red), PCNA and Sigma-3 (green) and representative confocal images of the mock and the 10^7 pfu conditions are shown in (A). Scale bar = 20μm. Number of cells with Sigma-3 expression were counted with ImageJ and divided by the number of panKRT+_DAPI+ cells (B). At least 4 fields were scored per technical replicate. Mean (SD) of N=2 (4 replicates).

Figure S5

A

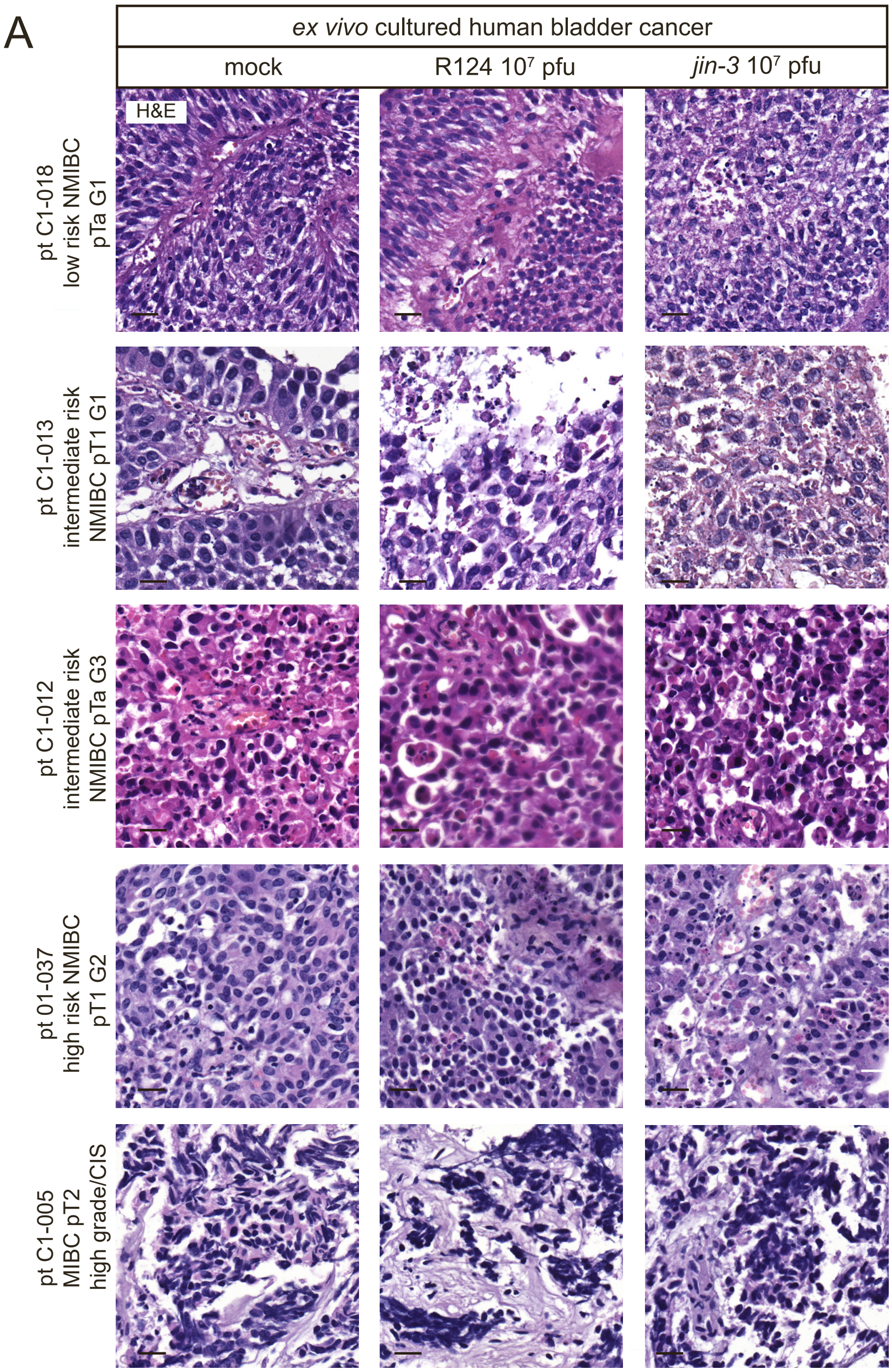
ex vivo cultured human bladder cancer
mock
R124 10^7 pfu
jin-3 10^7 pfu
H&E
pt C1-018
low risk NMIBC
pTa G1
pt C1-013
intermediate risk
NMIBC pT1 G1
pt C1-012
intermediate risk
NMIBC pTa G3
pt 01-037
high risk NMIBC
pT1 G2
pt C1-005
MIBC pT2
high grade/CIS

B

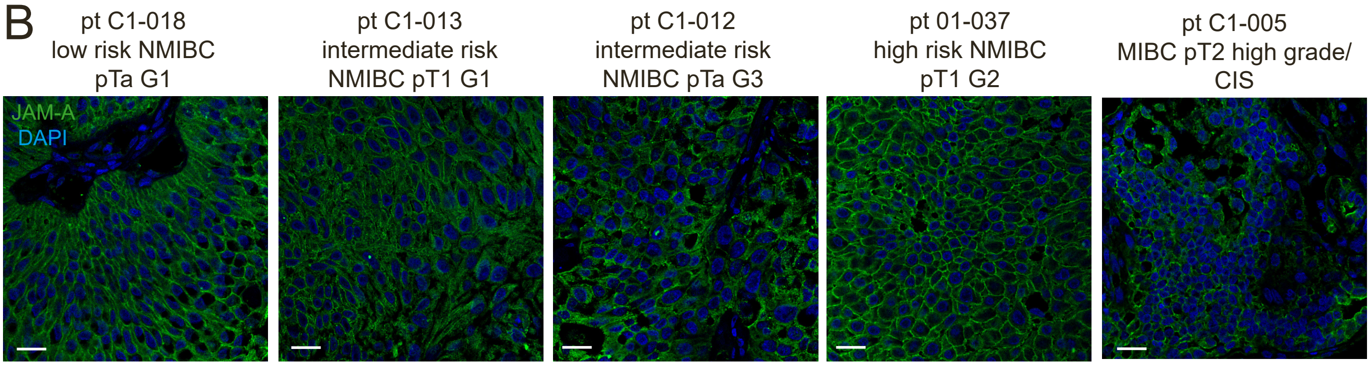
pt C1-018
low risk NMIBC
pTa G1
pt C1-013
intermediate risk
NMIBC pT1 G1
pt C1-012
intermediate risk
NMIBC pTa G3
pt 01-037
high risk NMIBC
pT1 G2
pt C1-005
MIBC pT2 high grade/
CIS
JAM-A
DAPI

Figure S5

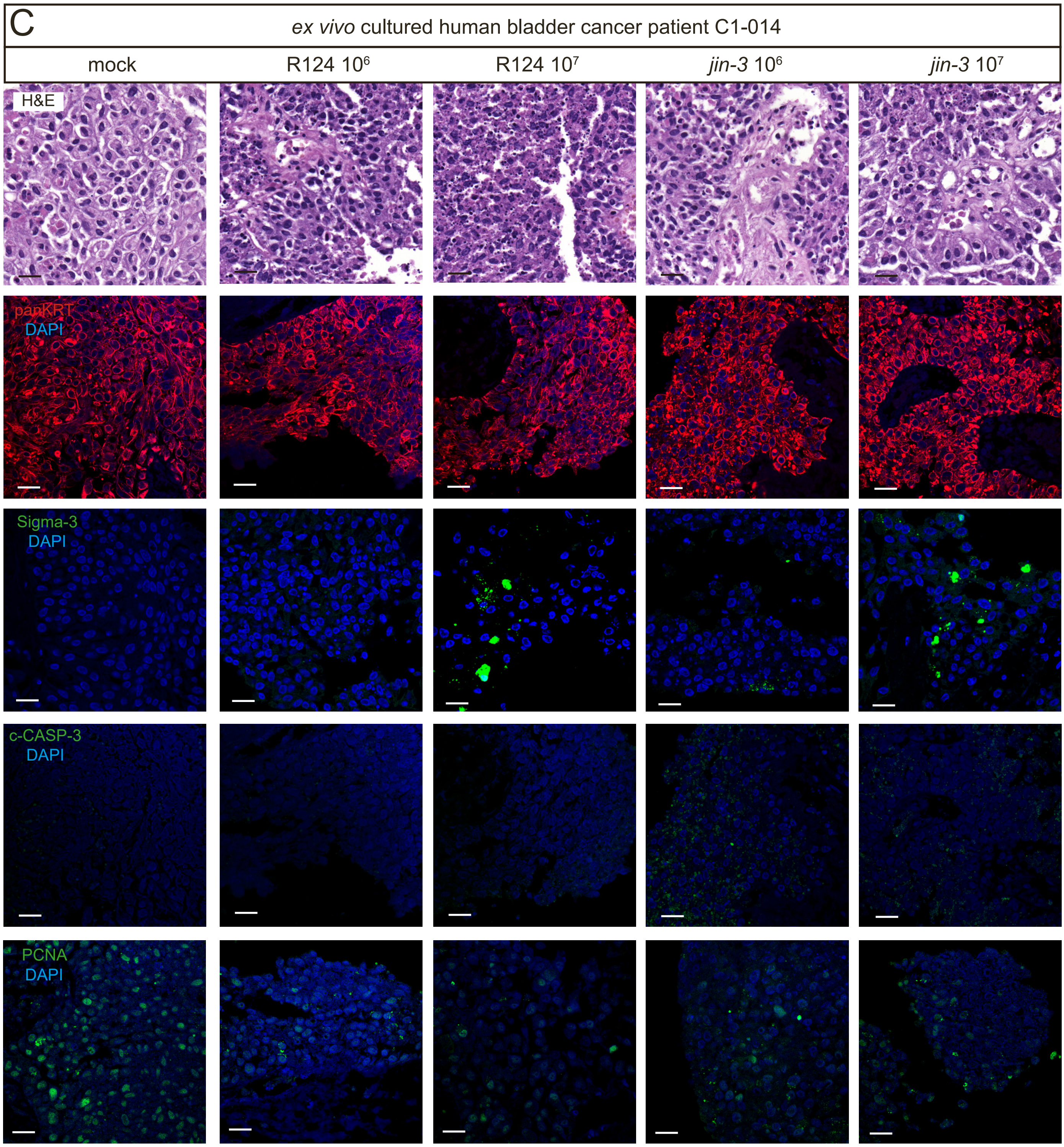

Figure S5: Comparison of *jin-3* and R124 reovirus infection in *ex vivo* cultured tumour tissue slices from human bladder cancer patients

Explanted tissue slices from patients (n=15) diagnosed with bladder cancer were exposed to either mock or reovirus R124 or *jin-3* for 3 days (dependent on the amount of tissue, either a complete dose range (mock, 10^6 and 10^7 pfu) or only the mock and 10^7 dosage). Tissues were stained for H&E. Scale bar = 25 μm. Multiple tissue slices were cultured per condition, representative images from 5 patients are shown. (B) Confocal images of JAM-A expression (JAM-A in green, and DAPI in blue) in mock treated *ex vivo* cultured patient tumour slices (n=5 are shown). (C) Representative confocal images from 1 patient are shown (pt C1-014; low risk NMIBC pTa low grade). Viral infection and replication in *ex vivo* cultured tissue slices with either R124 or *jin-3* reovirus. Green = either Sigma-3 (viral protein), c-CASP-3 (apoptotic cells) or PCNA (proliferating cells), Red = pan-cytokeratin (tumour cells), blue = DAPI (nuclei). Scale bar = 25 μm.

Figure S6

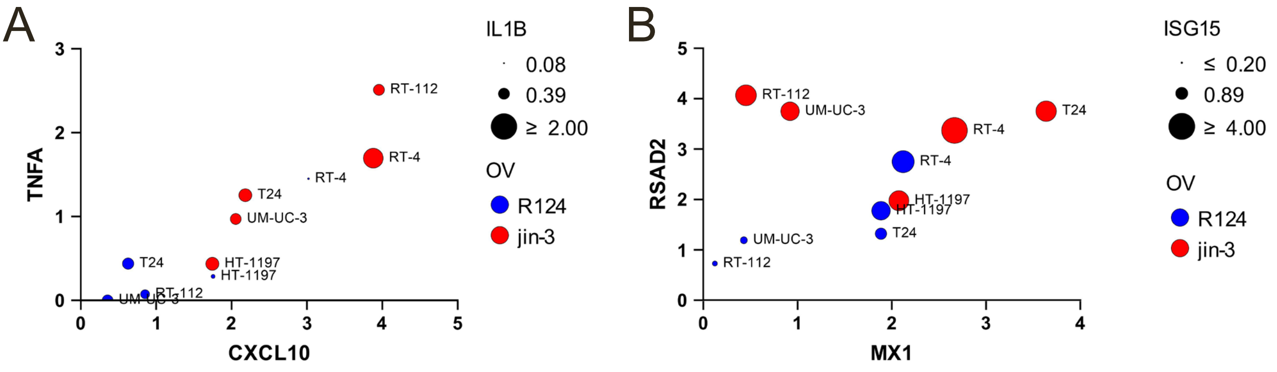

Figure S6 Comparison of immune modulation induced by *jin-3* and R124 reovirus treatment of bladder cancer cell lines (A) correlation file of mRNA expression of inflammatory cytokines *CXCL10* (x-axis), *TNFA* (y-axis) and *IL1B* (size of the dots) at MOI 10 of the indicated virus. (B) correlation file of mRNA expression of interferon stimulated genes *MX1* (x-axis), *RSAD2* (y-axis) and *ISG15* (size of the dots) at MOI 10 of the indicated virus.

Figure S7

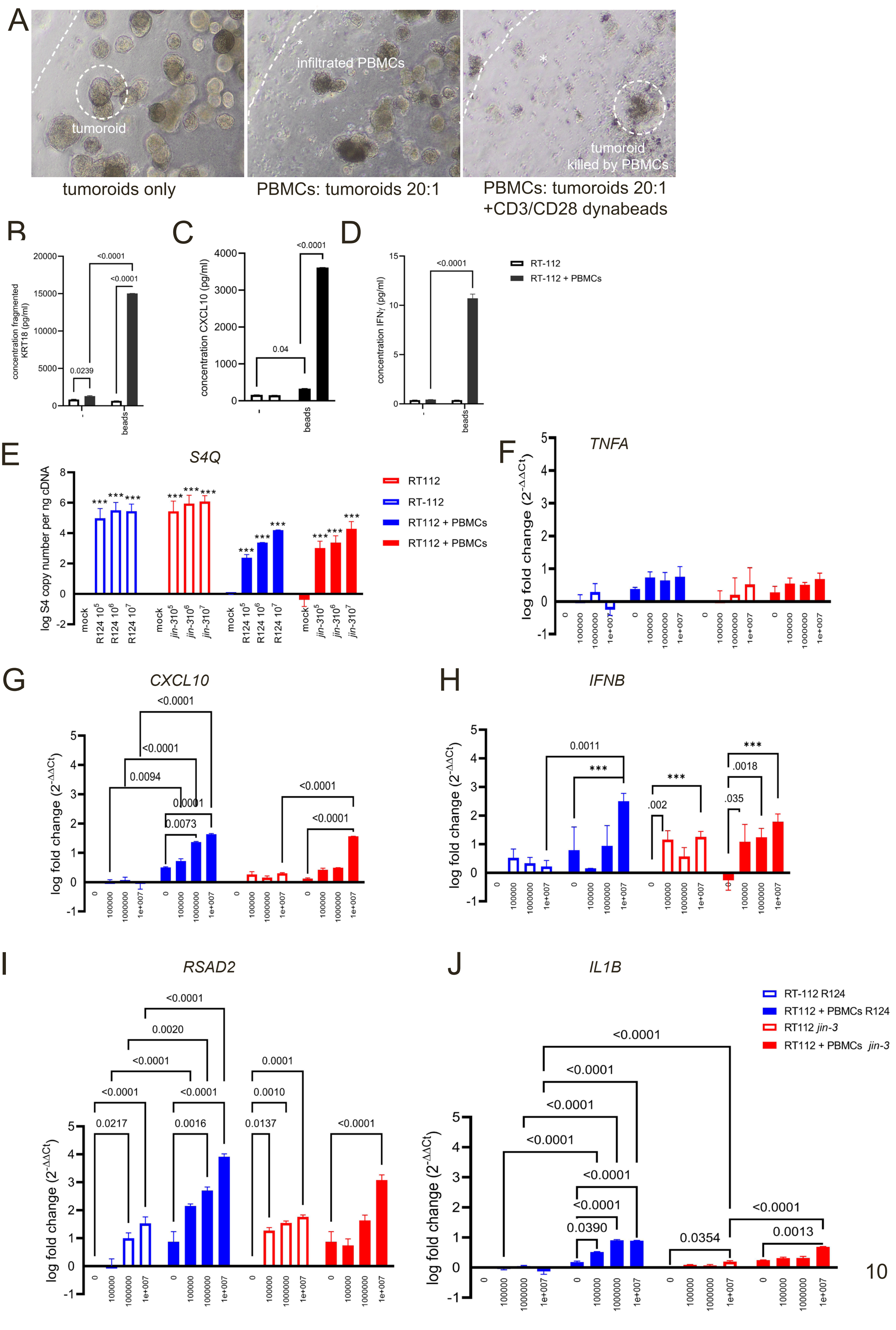
A
infiltrated PBMCs
tumoroid
tumoroid killed by PBMCs
tumoroids only
PBMCs: tumoroids 20:1
PBMCs: tumoroids 20:1 +CD3/CD28 dynabeads
B
concentration fragmented KRT18 (pg/ml)
C
concentration CXCL10 (pg/ml)
D
concentration IFNγ (pg/ml)
RT-112
RT-112 + PBMCs
beads
E
S4Q
log S4 copy number per ng cDNA
RT112
RT-112
RT112 + PBMCs
RT112 + PBMCs
F
TNFA
log fold change (2^-ΔΔCt)
G
CXCL10
H
IFNB
I
RSAD2
J
IL1B
RT-112 R124
RT112 + PBMCs R124
RT112 jin-3
RT112 + PBMCs jin-3

Figure S7

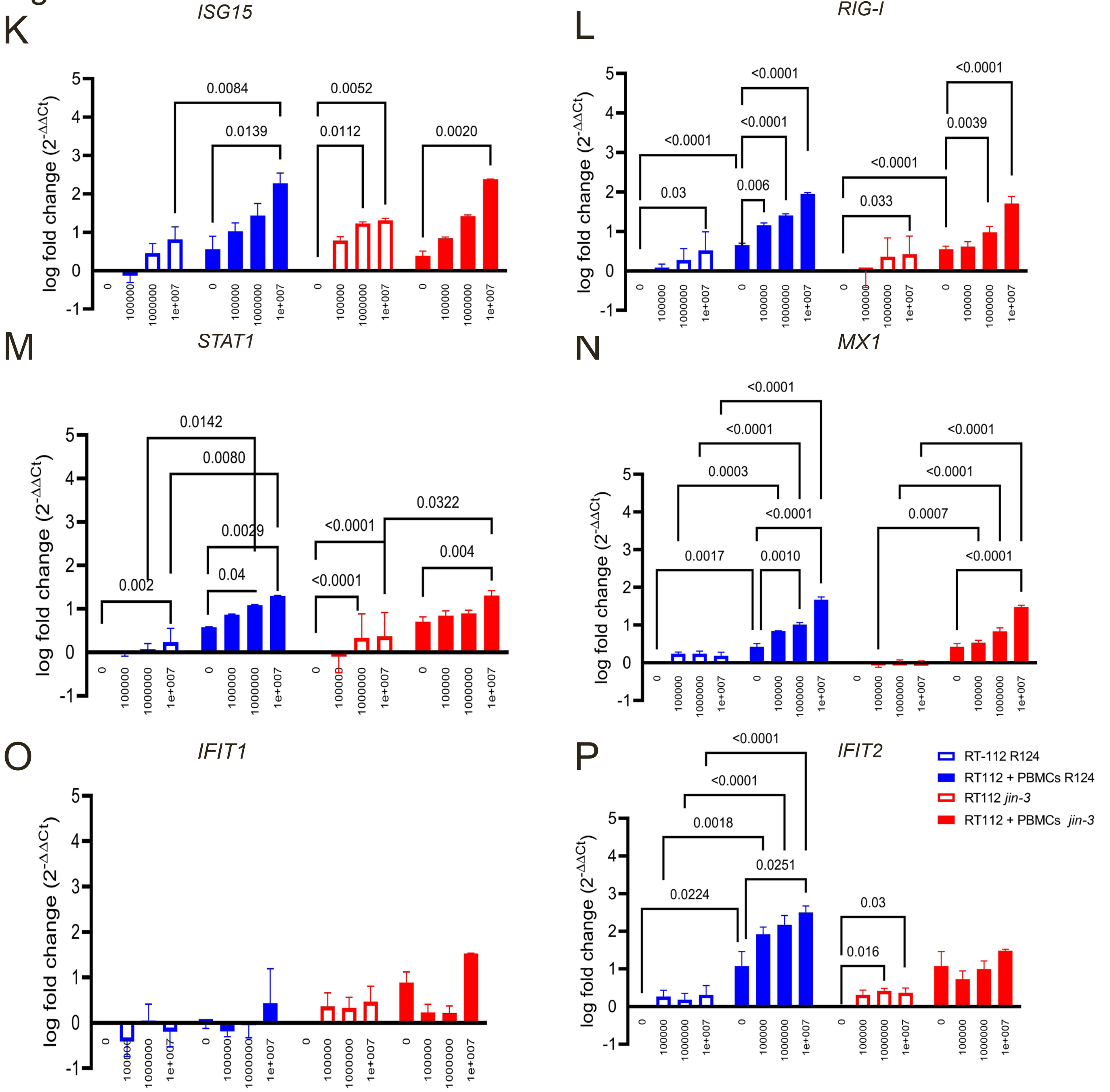

Figure S7 Viability and Immune modulation in RT-112 3D - PBMC co-culture models

(A) RT-112 bladder cancer cells were cultured in 60% matrigel and allowed to form 3D structures for approximately 3 days. Subsequently, PBMCs were added at an effector: target ratio of 20:1 in absence or presence of CD3/CD28 beads to activate the PBMCs. (B) fragmented epithelial marker KRT18 was determined as an outcome measure for tumour cell killing after 3 days of OV exposure. (C) CXCL10 levels and (D) IFNγ levels were measured after 3 days of OV exposure. (N=3, 2 replicates) Mean (SD). Two way ANOVA with Tukey's posthoc comparison (E) viral load (log fold change *S4Q* mRNA expression vs mock treated cells) in 3D bladder cancer cell line RT-112 exposed to a range of either R124 or *jin-3* reoviruses (10^5-10^7 pfu) in presence or absence of PBMCs. Expression levels of various inflammatory cytokines and interferon stimulated genes (log fold change mRNA expression ($2^{-\Delta\Delta Ct}$) vs mock treated cells) in 3D cultured RT-112 cells or RT-112 cells co-cultured with PBMCs exposed to either R124 or *jin-3* reoviruses. (F) *TNFA*, (G) *CXCL10*, (H) *IFNB*, (I) *RSAD2*, (J) *IL1B*, (K) *ISG15*, (L) *RIG-I*, (M) *STAT1*, (N) *MX1*, (O) *IFIT1*, (P) *IFIT2*. mean (SD) N=2. Two-way ANOVA followed by posthoc Tukey's.

Table S1 key resources (provided Excel file)
Key reagents and resources used in the manuscript with their respective suppliers, Research Resource Identifiers (RRID) and additional information. Cells were routinely cultured in a humidified incubator at 37°C and 5% CO2 and were regularly (once every 2 months) tested for mycoplasm by RT-PCR. Supplements were from Life Technologies, Gibco. For used antibodies, respective dilutions were added and the indicated time in the pressure cooker (PC) when antigen retrieval was performed by cooking the slides in unmasking solution (Vector Labs, H-3300); N.A. not applicable.

Supplemental Material and Methods

Virus production
Wildtype T3D reovirus strain R124 was plaque-purified from the wildtype reovirus T3D (ATCC) on HER911 cells.[1, 2] Reovirus mutant *jin-3* was isolated from JAM-A deficient U118MG cells after passaging of the wildtype T3D strain R124.[1] Both R124 and *jin-3* reoviruses were propagated, purified and titrated on human HER911 cells as previously described.[1]

Cell lines and culture conditions

Monolayer culture conditions
All human bladder cancer cell lines were cultured in monolayers with the respective culture medium and supplements (Supplemental table S1). Cells were incubated at 37°C in a humidified incubator with 5% CO2 and passaged every 3-4 days with fresh medium. Cells were regularly (once every 2 months) tested for mycoplasm by qRT-PCR.

3D tumoroid mono-culture conditions
Three-dimensional bladder cancer models were cultured from cells derived from tumours grown in PDX TM00024 model (PDXOs) [3] (Jackson Laboratory) or from RT-112 cells (tumoroids). Cells were seeded in domes at a density of 10,000 per 40 µL in 100% growth factor reduced, phenol red free, matrigel (Corning, 356231). 3D-cultures were incubated in 500 µl medium (respectively organoid culture medium [4] for the TM00024 3D cultures and RPMI supplemented with 10% FCS and P/S and Glutamax for the RT-112 3D cultures) at 37 °C in a humidified incubator with 5% CO2 and medium was refreshed every 3 to 4 days.

3D tumoroid-PBMC co-culture conditions
RT-112 cells were seeded in domes with a cell density of 10,000 cells per 40 µL matrigel (60% growth factor reduced, phenol red free, matrigel (Corning, 356231) with serum free DMEM (GibcoBRL 500ML 10567-014)). 3D-cultures were incubated in 500 µl medium consisting of 1:1 RPMI medium supplemented with 10% fetal bovine serum + P/S and RPMI supplemented with 20% human AB serum + P/S with 25 ng/ml IL-2 (Peprotech). After formation of 3D tumoroids, partially matched PBMCs were added at a 20:1 effector: target ratio. Reoviruses were added at the indicated dosages and the 3D co-cultures were cultured for an additional 3 days.

Ex vivo cultured tissue explants
Bladder cancer tissue derived from either CDX, PDX or directly from patient tumour material was sliced and cultured as previously described.[5] Slices were exposed to R124 or *jin-3* reovirus at the indicated dosages. Three days post exposure, the tissues were fixed with 4% PFA and processed for histology.
Tumour tissues from patients diagnosed with various stages of bladder cancer were obtained during transurethral resection of the bladder upon informed consent (table1, supplemental table S1; Biobank urogenital tumours protocol B20-010 evaluated by the METCC Leiden-The Hague-Delft). Criteria for the inclusion of patient derived tissue were 1) diagnosis of UCB in tissue slices (TS) is confirmed by the pathology report; 2) majority of directly fixed and/or mock-treated TS contain tumour cells; 3) size of obtained material is sufficient for at least 3 replicates per

condition.

Cell viability assay

Monolayer experiments viability

Using the CellTiter 96® AQueous One Solution Cell Proliferation Assay (Promega RRID:SCR_006724), viability was measured for all cell line/virus combinations over a range of multiplicity of infection (MOI). 1500 cells per well were seeded in a 96 wells plate. After 24 hours, these cells were exposed to either R124 or *jin-3* (control (mock) solution, (MOI) 0.01, 0.1, 1, 10 and or 100 plaque forming units (PFU)/cell). Cells were incubated for 72 or 144h, after which an MTS assay was performed according to manufacturer's directions. After 2h of incubation time, optical density was measured at 490nm using SpectraMax iD3 (Molecular Devices, San Jose, California USA) plate reader to assess cell viability.[6]

3D culture viability

Using the 3D CellTiter 96® Glo assay (Promega, G9681), viability of 3D-cultures was measured after R124 or *jin-3* infection. After 3 days of OV exposure, 250 µl of both culture medium and CellTiter glo were added to the wells. After 30 minutes incubation at room temperature on a plate shaker (300-500 rpm), luminescence was measured using the SpectraMax iD3 (Molecular Devices, San Jose, California USA) plate reader (integration time 1000ms/well, shake prior to measurement).

Flow cytometry

To assess levels of viral protein expression (Sigma-3), apoptosis (c-CASP-3), JAM-A and/or protein expression of several DAMPs (cell surface expression of calreticulin and HSP90) for each cell line/virus combination, 400,000 cells/well were seeded in a 6-wells plate. Each cell line was exposed to either R124 or *jin-3* (vehicle solution only or MOI 10 plaque forming units (PFU)/cell) and incubated for 48 hours. Subsequently, a single cell suspension of 100,000 cells/well was inserted in 96-wells V-bottom plates.

For the intracellular staining, cells were fixated in 4% paraformaldehyde (PFA) fixation buffer for 20 minutes at room temperature and washed using 1x ISP-buffer. Subsequently, cells were stained with Rabbit-anti-c-CASP-3 and mouse-anti-Sigma-3, with the unstained control cells receiving ISP-buffer only. Cells were incubated in the dark for 30 minutes on ice and afterwards washed twice using 1x ISP buffer. Cells were incubated with the secondary antibodies goat-anti-rabbit-APC and Donkey anti-Mouse IgG2a-Alexa Fluor 488 (with exception of the unstained conditions that only received ISP buffer) in the dark for 30 minutes on ice after which cells were washed and collected in ISP-buffer for flow cytometry.

For the cell surface staining, all cell line/virus combinations were stained for the following: control/unstained, IgG control, Calreticulin, HSP90 or JAM-A. All conditions except the unstained control were stained using live/dead aqua staining (LIVE/DEAD™ Fixable Aqua ARC Dead Cell Stain). After washing the single cell suspension with PBS, live/dead aqua staining was added and cells were incubated at room temperature for 20 minutes. The cells were then washed using FACS buffer (PBS, 1% FCS, 0.1% NaN3, 2 mM EDTA) and Fc receptor blocking was added, after which the plate was incubated for 10 minutes at 4°C. After washing with FACS buffer, IgG, Calreticulin and HSP90 primary antibodies were added to the appropriate wells with only FACS buffer for the control condition. This was incubated in the dark for 30 minutes on ice. Cells were then washed using FACS buffer and secondarily stained using Goat-anti-Rabbit APC. After 30 minutes incubation in the dark on ice, cells were washed with FACS buffer and fixed using PFA fixation buffer over 20 minutes at room temperature. Cells were then washed and collected in FACS buffer for flow cytometry.

Flow cytometry was performed using the LSR Fortessa™ X-20 Cell Analyzer (BD Biosciences Franklin Lakes, NJ United States) and data was analysed using FlowJoTM v10.8.1 (BD Biosciences Franklin Lakes, NJ, United States). For details about antibodies and dilutions used, see table S1

Histology and Immunofluorescent scoring

H&E and immunofluorescent stainings were executed as previously described (Supplemental table S1).5 H&E and Sigma-3 immunofluorescent stainings were scored using the Pannoramic MIDI slidescanner (3DHISTECH). All fluorescent stainings were visualized by confocal microscopy (63x magnification, resolution 1024x1024) (Leica SP8). Immunofluorescent stainings were scored by two independent reviewers. Multiple tissue slices were cultured per condition. At least 4 fields were scored per tissue slice. The average of these 4 fields was shown for each of the technical replicates. Cells expressing the respective proteins were counted with ImageJ and divided by the amount of panKRT+_DAPI+ cells (i.e. intact tumour cells with intact nuclei). The ratio fragmented tumour cells was measured by the number of fragmented cells divided by the number of total tumour cells.

Immunocytochemistry

Cells were seeded in 8 Chamber Slides at a density of 2000 cells/chamber. Cells were fixated for 10 minutes after 24, 48 and 72h of virus exposure at MOI 0.01, 0.1, 1, 10 and 100 in 4% PFA and stored at 4 degrees. Thereafter, cells were stained with a mouse anti-reoviral protein Sigma-3 primary antibody, followed by secondary staining with donkey-anti Mouse Alexa Fluor 488 (Thermo Fisher Scientific; for details see supplemental table S1). IgG isotype was used as a control staining. Subsequently, all wells were stained with DAPI. Immunofluorescence was visualized with confocal microscopy (SP8, Leica).

Real-time quantitative polymerase chain reaction (RT-qPCR)

Cells were seeded in 6 well plates and exposed to R124 or *jin-3* for 24 and 48 hours. Total RNA was isolated according to the manufacturer's protocol (Nucleospin RNA kit Macherey-Nagel). cDNA was generated by using random primers (Promega) and RT-qPCR was performed with GoTaq Mastermix (Promega) according to the manufacturer's protocol in technical duplicates and biological triplicates (Promega). Gene expression was normalized to GAPDH (∆CT). Fold change was measured using 2-∆∆Ct compared to mock treated sample. Primer sequences can be found in supplemental table S1. Viral S4Q copy number was calculated using the pcDNA S4 vector (supplemental figure S1B). 7

ELISAs

<u>High Mobility Group Box 1 (HMGB1)-release</u>

Cells were exposed to R124 or *jin-3* with a MOI of 10. HMBG1 release was measured by performing an Lumit™ HMGB1 Human/Mouse Immunoassay according to the manufacturer's protocol. In this assay, a pair of anti-human HMGB1 monoclonal antibodies were covalently labelled with complementary Small BiT (SmBiT; 11 amino acids) or Large BiT (LgBiT; 17.6 kDa) subunits (NanoBiTs) derived from NanoLuc® luciferase. These antibodies were incubated with sample. When the labelled antibodies recognized and bound to human HMGB1, the complementary NanoBiTs were brought into proximity, thereby reconstituting NanoBiT® luciferase and generating luminescence in the presence of Lumit™ Substrate. Light generated is directly proportional to the amount of analyte present in the sample. Luminescence was measured using the SpectraMax iD3 (Molecular Devices, San Jose, California USA) plate reader (37°C, integration time 1000ms/well, shake prior to measurement).

<u>Fragmented KRT18 ELISA</u>

3D cultures were exposed to R124 or *jin-3* at the indicated dosages. At the indicated time post-exposure, conditioned medium was collected and fragmented KRT18 levels were measured using the human cytokeratin 18 Fragment SimpleStep ELISA kit (CK18-30, Abcam).

CXCL10 ELISA
3D cultures were exposed to R124 or *jin-3* at the indicated dosages. At the indicated time post-exposure, conditioned medium was collected and CXCL10 levels were measured with Human IP-10 ELISA (Hu IP-10, Invitrogen).

IFN gamma ELISA
3D cultures were exposed to R124 or *jin-3* at the indicated dosages. At the indicated time post-exposure, conditioned medium was collected and IFNgamma levels were measured with the human IFN-gamma SimpleStep ELISA kit (ab174443, Abcam).

Statistical analyses
Statistical analyses were performed by using GraphPad Prism 8.0. Two-way ANOVA was performed with Tukey posthoc testing for multiple comparison. For *ex vivo* cultures, sample size was calculated using proportional odds models as described 8. Multiple slices were scored per condition per patient with multiple fields per slice.

REFERENCES

1 van den Wollenberg, D.J., Dautzenberg, I.J., van den Hengel, S.K., Cramer, S.J., de Groot, R.J., Hoeben, R.C., (2012). Isolation of reovirus T3D mutants capable of infecting human tumor cells independent of junction adhesion molecule-A. PloS one 7, e48064.
2 Dautzenberg, I.J., van den Wollenberg, D.J., van den Hengel, S.K., Limpens, R.W., Barcena, M., Koster, A.J., Hoeben, R.C., (2014). Mammalian orthoreovirus T3D infects U-118 MG cell spheroids independent of junction adhesion molecule-A. Gene Ther 21, 609-617.
3 Pan, C.X., Zhang, H., Tepper, C.G., Lin, T.Y., Davis, R.R., Keck, J., Ghosh, P.M., Gill, P., Airhart, S., Bult, C., et al., (2015). Development and Characterization of Bladder Cancer Patient-Derived Xenografts for Molecularly Guided Targeted Therapy. PloS one 10, e0134346.
4 Mullenders, J., de Jongh, E., Brousali, A., Roosen, M., Blom, J.P.A., Begthel, H., Korving, J., Jonges, T., Kranenburg, O., Meijer, R., et al., (2019). Mouse and human urothelial cancer organoids: A tool for bladder cancer research. Proc Natl Acad Sci U S A 116, 4567-4574.
5 van de Merbel, A.F., van der Horst, G., van der Mark, M.H., van Uhm, J.I.M., van Gennep, E.J., Kloen, P., Beimers, L., Pelger, R.C.M., van der Pluijm, G., (2018). An ex vivo Tissue Culture Model for the Assessment of Individualized Drug Responses in Prostate and Bladder Cancer. Front Oncol 8, 400.
6 van de Merbel, A.F., van der Horst, G., van der Mark, M.H., Bots, S.T.F., van den Wollenberg, D.J.M., de Ridder, C.M.A., Stuurman, D., Aalders, T., Erkens-Schulz, S., van Montfoort, N., et al., (2021). Reovirus mutant jin-3 exhibits lytic and immune-stimulatory effects in preclinical human prostate cancer models. Cancer Gene Ther.
7 Mijatovic-Rustempasic, S., Tam, K.I., Kerin, T.K., Lewis, J.M., Gautam, R., Quaye, O., Gentsch, J.R., Bowen, M.D., (2013). Sensitive and specific quantitative detection of rotavirus A by one-step real-time reverse transcription-PCR assay without antecedent double-stranded-RNA denaturation. J Clin Microbiol 51, 3047-3054.
8 Walters, S.J., (2004). Sample size and power estimation for studies with health related quality of life outcomes: a comparison of four methods using the SF-36. Health Qual Life Outcomes 2, 26.
9 Zuiverloon, T.C.M., de Jong, F.C., Costello, J.C., Theodorescu, D., (2018). Systematic Review: Characteristics and Preclinical Uses of Bladder Cancer Cell Lines. Bladder Cancer 4, 169-183.
