# Supplementary material for: Improved oncolytic and immunostimulatory activity of the spontaneous jin-3 reovirus mutant in preclinical bladder cancer models
Source: Mol Ther Oncol. 2026 Jan 13;34(1):201128. doi: 10.1016/j.omton.2026.201128 (PMC12860623; doi:10.1016/j.omton.2026.201128)
Supplement: Document S2. Article plus supplemental information [file mmc3.pdf]

Molecular Therapy
Oncology
Original Article

Improved oncolytic and immunostimulatory activity of the spontaneous *jin-3* reovirus mutant in preclinical bladder cancer models

Arjanneke F. van de Merbel,[1] Maaike H. van der Mark,[1] Lobke C.M. Hensen,[1] Diana J.M. van den Wollenberg,[2] Rob C. Hoeben,[2] Willemijn C.G. Zonneveld,[1] Rob C.M. Pelger,[1] Maxime T.M. Kummeling,[1] Geertje van der Horst,[1,3] and Gabri van der Pluijm[1,3]

[1]Department of Urology, Leiden University Medical Center, Albinusdreef 2, Leiden 2333ZA, the Netherlands; [2]Department of Cell and Chemical Biology, Leiden University Medical Center, Albinusdreef 2, Leiden 2333ZA, the Netherlands

Immunotherapy has emerged as a promising strategy for treatment of urothelial carcinoma of the bladder (UCB) but not all patients show clinically desirable responses upon treatment with immune-checkpoint inhibition. Oncolytic viruses may unleash the full potential of immunotherapy. Besides tumor-lysing properties, oncolytic viruses can induce durable and systemic anti-viral and anti-tumor immune responses. Here, we evaluated and compared oncolytic and immunostimulatory properties of wild-type reovirus (R124) and the junction-adhesion molecule-independent mutant reovirus (*jin-3*) in preclinical models of human UCB. Both reovirus variants effectively infected and replicated in human UCB cells and induced cell lysis in flatbottom or 3D cultures and in *ex vivo* cultured human tumor tissue slices. However, *jin-3* reovirus demonstrated greater efficacy in a dose-dependent manner, effectively inducing expression of immunogenic cell death markers, interferon (IFN)-stimulated genes, and inflammatory cytokines. To study interactions between tumor and immune cells, we established a co-culture system. In this context, co-culturing reovirus-infected bladder tumoroids with peripheral blood mononuclear cells was found to significantly enhance cancer death dose-dependently, alongside induction of key immune mediators such as CXCL10 and IFN-γ. Together, reoviruses display strong oncolytic properties in preclinical UCB models. *jin-3* reoviruses elicit robust immunostimulatory responses, highlighting their potential as candidate agents for clinical translation in UCB.

INTRODUCTION

Urothelial carcinoma of the bladder (UCB) is the seventh most prevalent cancer globally, with a 5-year prevalence surpassing 5 million cases.[1] Approximately 75% of newly diagnosed cases encompass non-muscle invasive bladder cancer (NMIBC), which are usually removed by transurethral resection.[2] Patients are then categorized as low, intermediate, or high risk for recurrence and progression according to the guidelines of the European Urological Association.[3] Current therapy for patients with intermediate- and high-risk NMIBC includes immunotherapy with Bacillus Calmette-Guérin (BCG) or chemotherapy. To date, BCG instillations have proven to be the most successful adjuvant therapy.[3,4] However, 30%–50% of the patients fail to respond to BCG therapy, and 15% of patients show progression to the muscle invasive disease, MIBC.[2,5,6] These MIBC patients have a relatively poor prognosis (5-year survival of 40%–60%). UCB recurs frequently after initial treatment and requires long-term clinical monitoring, causing substantial morbidity and diminished quality of life.[4,7,8] Immunotherapy (including immune checkpoint inhibition [ICI], cell-based therapy, and cancer vaccines) is most effective in tumors with relatively high somatic mutation rates as is the case for UCB.[9–15]

ICIs, including pembrolizumab, nivolumab, atezolizumab, durvalumab, and avelumab, have improved the treatment landscape for bladder cancer, particularly for metastatic urothelial carcinoma.[16]

These ICIs have shown impressive and durable responses in bladder cancer patients.[17–28] In high-risk NMIBC patients who are unresponsive to BCG, 41% of patients display a clinically desirable response to pembrolizumab (i.e., absence of high-risk NMIBC or progressive disease after 3 months).[29] About 30% of patients with metastatic MIBC will respond to ICI treatment (objective response rate and overall survival),[30] although these benefits appear to be limited to patients with pre-existing anti-tumor responses.[25,31–33]

Currently, ICIs are standard-of-care options in multiple clinical scenarios: as first-line therapy for cisplatin-ineligible patients, second-line therapy after platinum-based chemotherapy failure, and in novel combination regimens.

Received 18 August 2025; accepted 9 January 2026;
https://doi.org/10.1016/j.omton.2026.201128.

[3]These authors contributed equally

Correspondence: Geertje van der Horst, Department of Urology –J3-100, Albinusdreef 2, 2333ZA Leiden, the Netherlands.
E-mail: g.van_der_horst@lumc.nl

© 2026 The Authors. Published by Elsevier Inc. on behalf of The American Society of Gene and Cell Therapy. This is an open access article under the CC BY license (http://creativecommons.org/licenses/by/4.0/).

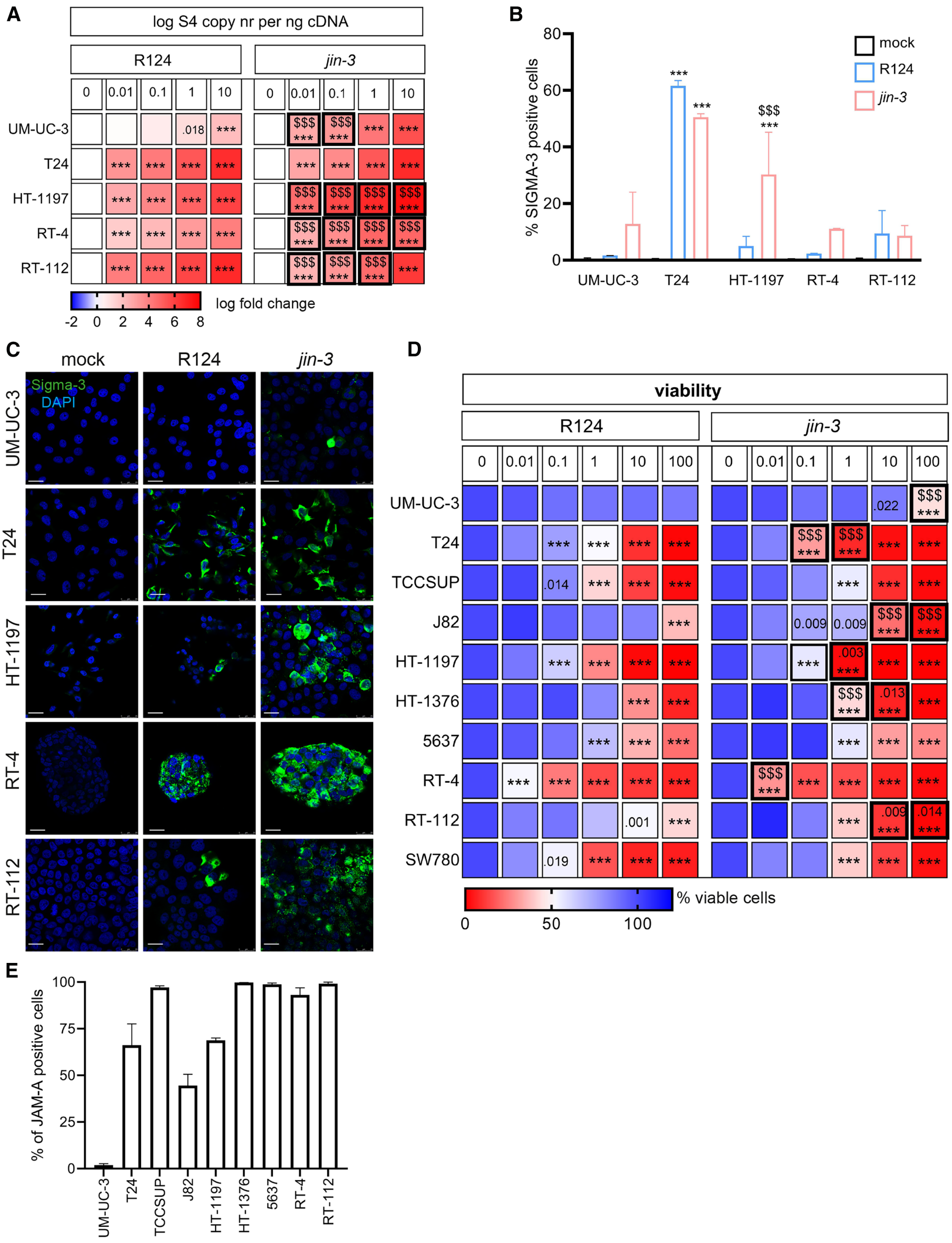

Figure 1. Comparison of the oncolytic effects of mutant *jin-3* versus R124 reovirus in bladder cancer cell lines *in vitro*

(A) Mean viral S4Q copy number (log fold change S4Q mRNA expression vs. mock treated cells) in bladder cancer cell lines exposed to a range of MOI (0.01–0.1–1–10) of either R124 or *jin-3* reoviruses after 24 h. In red, the highest infection with virus is shown. Two-way ANOVA followed by Tukey's post hoc test. $n = 3$ (2 replicates). UM-UC-3 cells R124 reovirus MOI1 vs. mock $p = 0.0184$; ***$p < .001$, \$\$\$ $p < .001$, asterisks indicate mock versus reovirus infection, and dollar signs indicate R124 versus *jin-3.* (B) Viral load (% of viable, single cells expressing Sigma-3 protein) in bladder cancer cell lines exposed to MOI10 of either R124 or *jin-3* after 72 h mean (SD) ($N = 3$). Two-way ANOVA followed by Tukey's post hoc test. ***$p < .001$, \$\$\$ $p < .001$, asterisks indicate mock versus reovirus infection, and dollar signs indicate R124 versus *jin-3.* (C) Confocal

(legend continued on next page)

Despite these encouraging results in a subgroup of UCB patients, treatment modalities that can further improve immunotherapy are urgently required for unresponsive bladder cancer patients.

Oncolytic viruses represent a possible new approach since these viruses can infect, replicate, and lyse malignant tumor cells, while minimizing harm to normal cells.[34] Furthermore, oncolytic viruses can activate the adaptive and innate immune system[35–38] via the induction of immunogenic cell death (ICD), a type of cell death that triggers immunity against tumor antigens of dying cells, resulting in the exposure of damage-associated molecular patterns (DAMPs), e.g., HMGB1 release.[39,40] DAMPs can activate antigen-presenting cells, which subsequently can stimulate the expansion and activation of effector T cells, including cytotoxic CD8+, helper CD4+ T cells, and natural killer (NK) cells.[41,42]

Reoviruses are double-stranded RNA viruses and have not been associated with severe disease in humans.[43] Mutant reovirus *jin-3* harbors a mutation in the spike protein Sigma-1 and can infect cells via the high-affinity receptor junction adhesion molecule A (JAM-A) but also independently of JAM-A on the tumor cell surface by high-affinity binding to negatively charged sialic acids. We previously demonstrated that *jin-3* reoviruses display extended tumor tropism compared to the wild-type reovirus.[44,45] Interestingly, this JAM-A protein is involved in various biological processes including tight junctions involved in epithelial barrier function.[46] Expression levels of JAM-A in various cancers have been analyzed using multiple databases, including TIMER2.0, GEPIA2, and the UALCAN portal and was shown to be upregulated in multiple cancers.[47] JAM-A was significantly upregulated in urothelial carcinoma compared to normal bladder. However, bladder cancer patients with low JAM-A expression showed decreased overall survival.[47] Potentially, this reduced overall survival could be due to the acquisition of a more mesenchymal invasive phenotype (EMT) by the cancer cells due to the shedding of epithelial tight junctions.[47] Mutational profile analysis (cBioportal based on TCGA datasets) revealed that JAM-A was altered in more than 15% of bladder cancer patients experiencing either amplification or mutation of JAM-A.[47]

In this study, the direct oncolytic and indirect immunomodulatory effects of reovirus mutant *jin-3* were determined and compared to those of the wild-type T3D reovirus R124 in state-of-the-art preclinical bladder cancer models, including monolayer cultures and three-dimensional cell (co-) cultures and *ex vivo* cultured bladder tumor tissue slices derived from patients with disease ranging from low-grade NMIBC to MIBC.

RESULTS

Direct cytolytic effects of *jin-3* and R124 reoviruses

A panel of human bladder cancer cell lines of different molecular subtypes was exposed to a dose range of either R124 or *jin-3*, and viral expression levels were detected by RT-PCR after 24 h. In the UM-UC-3, HT-1197, and RT-4 cell lines, more viral transcripts were detected in the cells exposed to *jin-3*, whereas in the T24 and RT-112 cell lines either no difference or less viral transcripts were found (Figures 1A and S1A–S1C; Table S1). In addition, viral protein levels of Sigma-3 were determined in various tumor cells by flow cytometry (Figure 1B) or immunofluorescence (Figure 1C) after 72 h of treatment. A similar Sigma-3 pattern was observed, i.e., increased level of expression in *jin-3*-treated UM-UC3, HT-1197, and RT-4 and a similar expression level of Sigma-3 in T24 and RT-112 cell lines.

Both *jin-3* and R124 effectively killed the majority of bladder cancer cells in a dose- and time-dependent manner (Figures 1D and S1D). In cell lines of different type (MIBC or NMIBC; see Table S1) and molecular cancer subtypes, *jin-3* and R124 displayed comparable anti-tumor effects in 5637 (mixed phenotype), RT-4 (luminal phenotype), and SW780 (basal phenotype). In the HT-1197, HT-1376 (both mixed phenotype), and RT-112 cells (luminal phenotype), *jin-3* was found to be more effective.[48] In the basal molecular subtype bladder cancer cell panel, T24 cells were significantly more effectively killed by *jin-3* compared with wild-type reovirus R124, while both viruses were equally effective in TCCSUP cells. Strikingly, while R124 viruses did not affect the viability of the basal type UM-UC-3 cells, *jin-3* reduced their viability. Moreover, in J82 cells, R124 viruses resulted in decreased viability only after exposure to high MOI, whereas exposure to *jin-3* resulted in reduced viability at lower MOI.

R124 reoviruses enter cancer cells via its high-affinity receptor junction adhesion molecule A (JAM-A),[44,49,50] while the spontaneous mutant *jin-3* virus has a broader tropism due to its ability to also infect cancer cells independently of JAM-A, i.e., by binding negatively charged cell surface sialic acids.[44]

As expected, the expression levels of the canonical viral entry receptor JAM-A in tested cancer cell lines were in line with the ability of the reovirus variants to infect and/or replicate in the tumor cells (Figures 1E and S1G).

Upon challenge with R124 and *jin-3,* a dose-dependent increase in viral copy number was detected in 3D cultures of RT-112 cells (Figure S1H), coinciding with reduced viability of the treated tumoroids after 3 days (Figure S1I).

In organoids cultured from the patient-derived muscle-invasive bladder cancer xenograft model PDX TM00024, R124 and *jin-3* showed dose-dependent infection and replication (Figure 2A).[51] Furthermore, a significant reduction in cancer cell viability was observed after reovirus exposure for 7 days (Figure 2B). Viral protein

images of viral protein (Sigma-3, green) and DAPI (blue)-stained bladder cancer cells treated with MOI10 of R124 or *jin-3* for 72h. Scale bars, 25 μm. (D) Mean percentage of viable cells after exposure to a range of MOI of either R124 or *jin-3* for 6 days. $n = 3$ (6 replicates). p values are depicted (vs. mock; when 2 depicted upper p value is R124 vs. *jin-3*); ***$p < .001$, \$\$\$ $p < .001$, asterisks indicate mock versus reovirus infection, and dollar signs indicate R124 versus *jin-3.* (E) Percentage of single, viable, JAM-A protein expressing cells. Mean (SD), $n = 3$. MOI = multiplicity of infection.

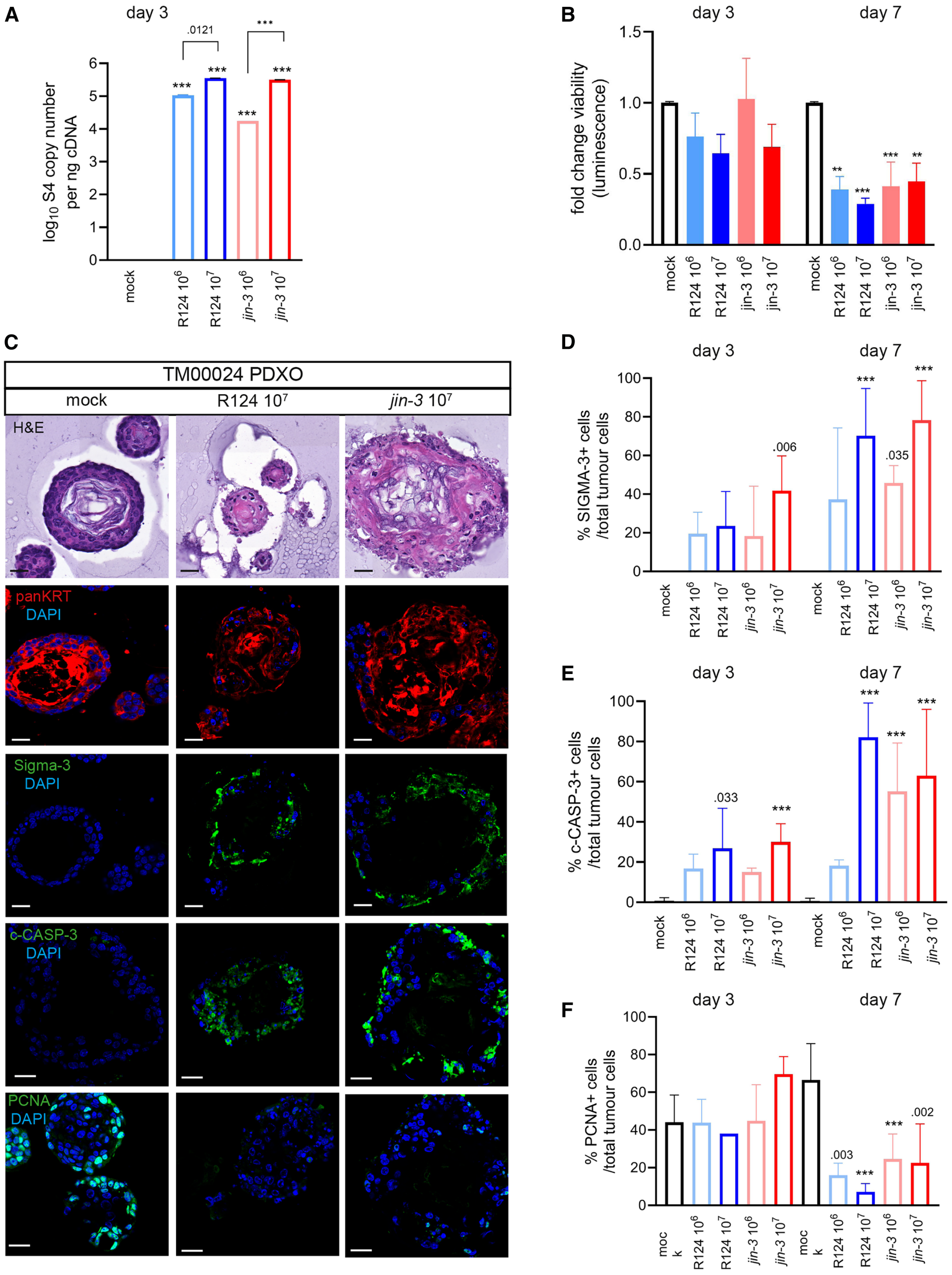

Figure 2. Comparison of the oncolytic effects of reovirus mutant *jin-3* versus wild-type reovirus R124 in 3D cultures *in vitro*

TM00024 bladder PDXOs were exposed to the indicated plaque-forming units (PFUs) of reoviruses for 3 or 7 days.

(A) Mean (SD) viral copy number of TM00024 PDXOs treated for 3 days with the indicated PFUs of the reoviruses. Two-way ANOVA followed by Tukey's post hoc comparison ($n = 3$) ***$p < .001$. (B–F) Viability (mean [SD] was measured using cell titer glo 3D after days 3 and 7 of reovirus exposure. $N = 3$ (3 replicates). Two-way ANOVA followed by

(legend continued on next page)

(Sigma-3) was detected in the outer cell layers of the PDXOs, indicating active viral infection and replication after 3 days (Figures 2D and S2A).[52,53] Moreover, an increased number of c-CASP-3-positive cells was measured (Figures 2E and S2A). Further increase of the Sigma-3- and c-CASP-3-positive cells was observed after 7 days (Figures 2C–2E and S2B for dose range), coinciding with a decrease in cell proliferation (nuclear PCNA; Figures 2C, 2F, and S2B) and loss of structural integrity of the PDXOs (Figures 2C and S2B). No significant differences were observed between the viruses.

Subsequently, viral infection and replication was determined in tumor tissue slices derived from cell-line-derived xenograft (CDX) and patient-derived xenograft (PDX) models after reovirus exposure. Upon exposure of tumor tissue slices of RT-112 bladder cancer cells[54] to R124 and *jin-3 ex vivo* for 3 days, the viruses were capable of infecting the cancer cells and replicated in viral factories in the cell (Figures 3A and 3B). Furthermore, apoptosis was stimulated upon reovirus exposure as assessed by a relative increase of c-CASP-3-positive cells coinciding with a decline in tumor cell proliferation (Figures 3C–3E and S3A for dose range). In another CDX model J82 (Figure S4), similar results were obtained. Moreover, in *ex vivo* cultured tumor slices obtained from the PDX model TM00024, which displayed cell membranous JAM-A protein expression (Figure S3C), reovirus exposure also induced increased apoptosis and impaired tumor cell proliferation (Figures 3F–3J and S3B). No significant changes were observed between the two viruses.

Consistent with earlier findings,[47] JAM-A expression was upregulated in bladder cancer compared to normal bladder (TCGA-BLCA database), although low levels of JAM-A are observed in 103/412 samples (Figures S1E and S1F). Next, tumor tissue slices were generated from freshly obtained tissues from patients diagnosed with various stages of bladder cancer ($n = 15$; Table 1). Tissue slices were exposed to mock, R124, or *jin-3* reoviruses for 3 days (Figures 4 and S5). Immunolocalization of JAM-A was determined in the patient tissues, and membranous localization was observed in all the patients, with one patient displaying lower levels of staining (pt C1-005; Figure S5B). Explanted tumor tissue was stained for c-CASP-3, PCNA (proliferation) and integrity of the cells (H&E staining and panKRT/DAPI staining). In the reovirus-treated patient-derived *ex vivo* cultured tumor explants, interpatient heterogeneity in Sigma-3 protein levels was observed, with Sigma-3 expression found in regions in multiple tissue slices per patient (Figures 4A and 4B). Exposure to reoviruses resulted in increased number of c-CASP-3-positive cells (Figures 4C and S5C), whereas the number of proliferating tumor cells was reduced (Figures 4D and S5C) as well as the integrity of the cells as indicated by the H&E staining and the increased ratio of fragmented cells (Figures 4E and S5A–S5C).

Reovirus-induced immune activation

Inflammatory cytokines, type I interferons (IFNs), and IFN-stimulated genes (ISGs) are of key significance in oncolytic virotherapy that largely relies on the stimulation of anti-tumor and anti-viral immune cell activation.[55–57] Exposure to R124 and *jin-3* reovirus resulted in a significant and dose-dependent upregulation of IFN-β gene expression (*IFNB1*) after 24 h in multiple bladder cancer cell lines (Figures 5A–5G). *jin-3* treatment resulted in a significantly stronger upregulation in *IFNB1* (6/7) compared to R124 (4/7) in all the tested cell lines (Figures 5C–5G), except for UM-UC-3 and T24 (Figures 5A and 5B). Moreover, exposure to *jin-3* led to significantly stronger induction of ISGs (i.e., *ISG15*, *IFIT1*, *IFIT2*, *RSAD2*, and *MX1*) in most of the tested cell lines compared to R124 (Figure 5). Exposure to *jin-3* led to a superior and significant expression of inflammatory cytokines *CXCL10* (in 7/7 cell lines), *TNF* (4/7), *and IL1B* (2/7) compared with R124 (Figure 5). Moreover, the cytosolic RNA sensor *RIG-I* was significantly upregulated after *jin-3* exposure compared to R124 in 6/7 cell lines (Figure 5). In the TM00024 PDXOs, exposure to *jin-3* reovirus resulted in significant upregulation of *TNF*, *CXCL10*, *IFNB1*, and *RSAD2* gene expression, whereas R124 reovirus only induced *TNF* gene expression (Figure 5H).

Cell surface expression of the DAMPs ecto-calreticulin and ecto-HSP90 was significantly increased upon exposure to reoviruses (Figures 6A and 6B). Strikingly, *jin-3* caused a more pronounced increase in HMGB1 release, a well-established marker of immunogenic cell death[42] (Figure 6C). Correlation between these markers at an MOI of 10 is depicted in Figure 6D.

Subsequently, co-culturing of 3D tumoroids of human RT-112 cells with partially HLA-matched peripheral blood mononuclear cells (PBMCs) led to increased tumoroid killing (Figure S7A). This was accompanied with significantly increased release of fragmented cytokeratin-18 (KRT18; Figure S7B), CXCL10 (Figure S7C), and IFN-γ (Figure S7D). Addition of reoviruses to the 3D cultures resulted in expression of viral transcripts in the reovirus-treated RT-112 cells, with a lower copy number when the 3D cultures were co-cultured with PBMCs (Figure S7E). Reovirus treatment in the co-cultures resulted in augmented tumoroid killing compared to the mock-treated conditions, with *jin-3* displaying significantly more pronounced effects (Figures 7A and 7B). Moreover, chemo- and cytokine production (CXCL10 and IFN-γ) and mRNA expression of type I IFN response genes (e.g., *IFNB1*, *RSAD2*, *MX1*, *IFIT2*, and *ISG15* mRNA levels) was significantly enhanced upon reovirus treatment in these cultures (Figures 7C–7E and S7F–S7P).

Tukey's post hoc comparison. ***$p < .001$. (C) Confocal images of PDXOs (day 7) stained for respectively H&E, panKRT (red); Sigma-3, c-CASP3, or PCNA (green); and DAPI (blue). Scale bars, 20 μm. Cells expressing the respective proteins Sigma-3 (D), c-CASP-3 (E), and nuclear PCNA (F) were counted with ImageJ and divided by the amount of panKRT+_DAPI+ cells. At least five fields were scored per technical replicate. Mean (SD) of $N = 3$ (3 replicates). ***$p < .001$, asterisks indicate reovirus infection versus mock same day. Two-way ANOVA followed by Tukey's post hoc comparison.

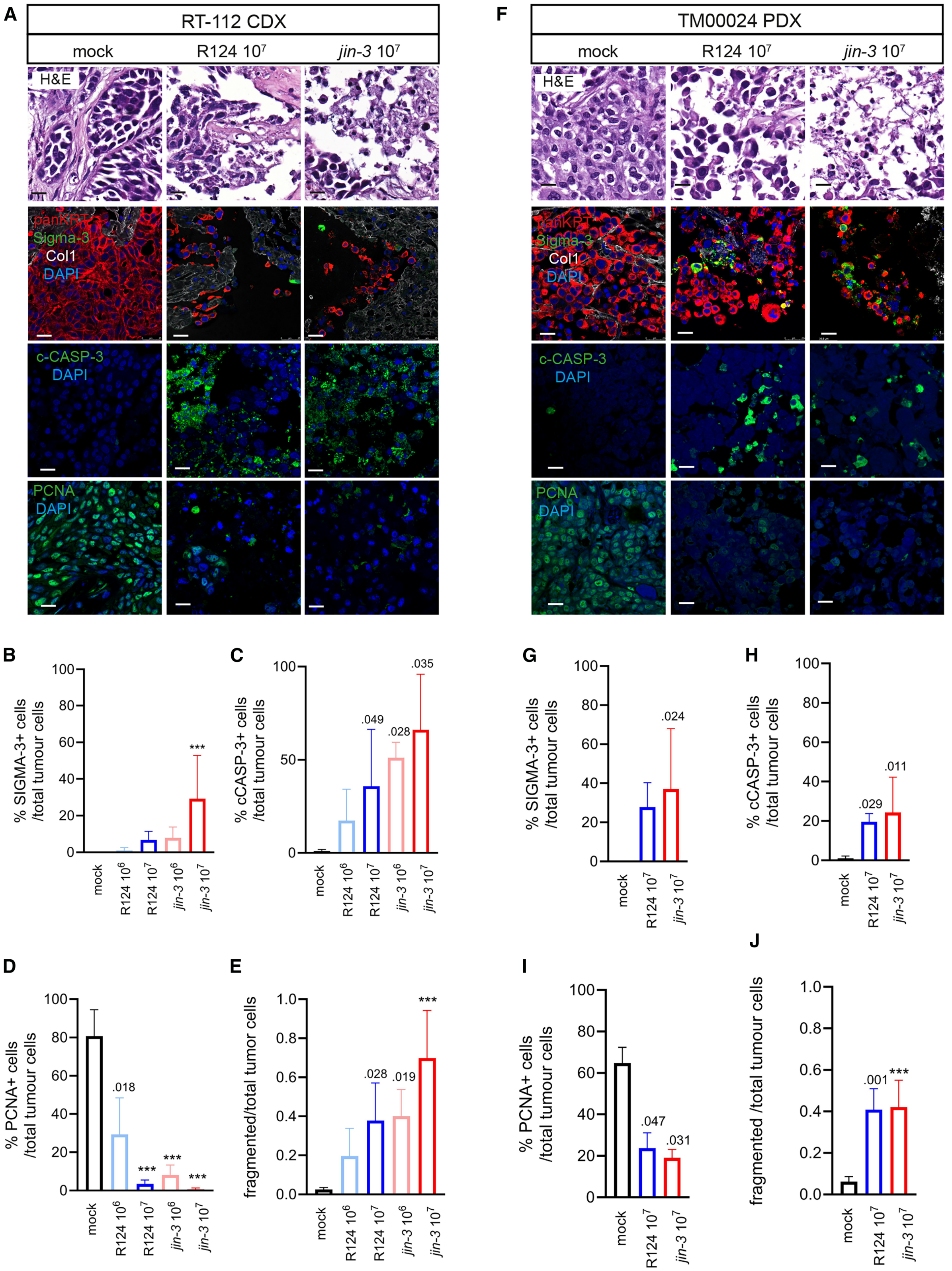

(legend on next page)

Table 1. Patient information: clinical histopathological characteristics of the patients included. Disease stage and grade at time of sampling of the tumor material

Patient identifier	Disease	Risk group	[55]Disease stage	Disease grade	Treatment history	Age	Gender	CIS
C1-005	MIBC	–	pT2	high-grade CIS	BCG bladder instillations	70	male	yes
01–032	MIBC	–	pT2	G3	unknown	69	male	no
C1-015	NMIBC	very high	pT1	focal G3	none	61	male	no
01–037	NMIBC	high	pT1	G2	none	82	female	no
C1-020	NMIBC	high	pTa/pT1	G3	none	76	male	no
C1-013	NMIBC	intermediate	pT1	low grade	none	87	male	no
C1-012	NMIBC	intermediate	pTa	G3	none	77	male	no
01–034	NMIBC	intermediate	pTa	G2	none	80	male	no
C1-008	NMIBC	intermediate	pT1	G1	none	62	male	no
C1-022	NMIBC	intermediate	pTa	G1	none	79	male	no
C1-047	NMIBC	intermediate	pTa	G1	none	64	male	no
C1-086	NMIBC	low	pTa	G1	none	67	male	no
C1-088	NMIBC	low	pTa	G1	none	73	male	no
C1-018	NMIBC	low	pTa	G1	none	67	male	no
C1-014	NMIBC	low	pTa	low grade	none	70	female	No

pTa, p(pathological) non-invasive papillary carcinoma; pT1, tumor invades subepithelial connective tissue; pT2, tumor invades muscle. Grading was scored using the WHO grading 1973 (G1: well differentiated; G2: moderately differentiated; G3: poorly differentiated) or WHO 2004 (low/high grade) MIBC, muscle-invasive bladder carcinoma; NMIBC, non-muscle invasive bladder carcinoma; CIS, carcinoma *in situ*; BCG, Bacillus Calmette-Guerin.

DISCUSSION

In this study, we evaluated the direct oncolytic and immunomodulatory effects of reoviruses R124 and *jin-3* in preclinical human bladder cancer models.

We observed that reoviruses induce potent oncolytic effects in flatbottom 2D and 3D cultures of human bladder cancer. Despite the differences in oncolytic responses, which are likely to be due to well-documented clinical inter-tumoral heterogeneity in bladder cancer,[58,59] we observed viral infection upon reovirus administration in most human bladder cancer cells. Moreover, we detected viral infection and replication in near-patient *ex vivo* cultured bladder cancer tissue slices derived from CDX and PDX models and primary bladder cancer patient material.

Besides oncolytic properties, oncolytic viruses can induce both anti-viral immunity and anti-tumor immune responses, which is required for long-lasting and systemic tumor control.[60] Effective oncolytic virotherapy relies on the production of pro-inflammatory cytokines and type I IFNs and the induction of interferon-stimulated genes (ISGs). Pro-inflammatory chemokines and cytokines are released and attract lymphocytes toward the tumor.[60–62] These anti-viral immunological events could turn immunological “cold” tumors into “hot” tumors. Our study demonstrated that *jin-3* reovirus led to elevated expression of ISG and inflammatory cytokine production by infected bladder cancer cells compared to R124 wild-type reovirus. This is further substantiated by the augmented release of immunogenic cell death markers HMGB1 and DAMPs (i.e., ectopic cell surface expression of calreticulin and HSP90 after exposure to *jin-3* reovirus). Co-culturing of bladder tumoroids with PBMCs resulted in a significant, dose-dependent increase in tumor cell death and elevated production of CXCL10 and IFN-γ after reovirus exposure. This is consistent with findings in a colorectal cancer co-culture system in which autologous PBMCs acquire tumor-targeting capabilities, which correlated with clinical responses to checkpoint inhibitors.[63]

Overall, our preclinical results support the notion that infection of the human bladder cancer cells with reoviruses, especially *jin-3*, induces oncolysis and may contribute to a switch from an immunologically “cold” toward an immunological “hot” inflammatory phenotype. Additionally, *jin-3* has an extended tropism compared to the wild-type reovirus because these viruses can infect cells independently of JAM-A expression on the tumor cell surface. These

Figure 3. Comparison of *jin-3* and R124 reovirus infection in *ex vivo* cultured tumor tissue

Explanted tissue slices from either RT-112 CDX (A–E) or TM00024 PDX (F–J) were exposed to the indicated PFU of R124 or *jin-3* reovirus for 3 days. Tissues were stained for H&E, panKRT (red); Sigma-3, c-CASP3 or PCNA (green), type I collagen (white), and DAPI (blue). Representative confocal images are shown. Scale bars, 20 μm. Cells expressing the respective proteins were counted with ImageJ and divided by the number of panKRT+_DAPI+ cells. At least four fields were scored per technical replicate. Mean (SD) of $N = 2$ (4 replicates). The ratio fragmented tumor cells was measured by the number of fragmented cells/total tumor cells (E and J). $^{***}p < .001$, reovirus infection versus mock. Mean (SD), $n = 2$ (4 replicates). Two-way ANOVA followed by Tukey’s post hoc comparison.

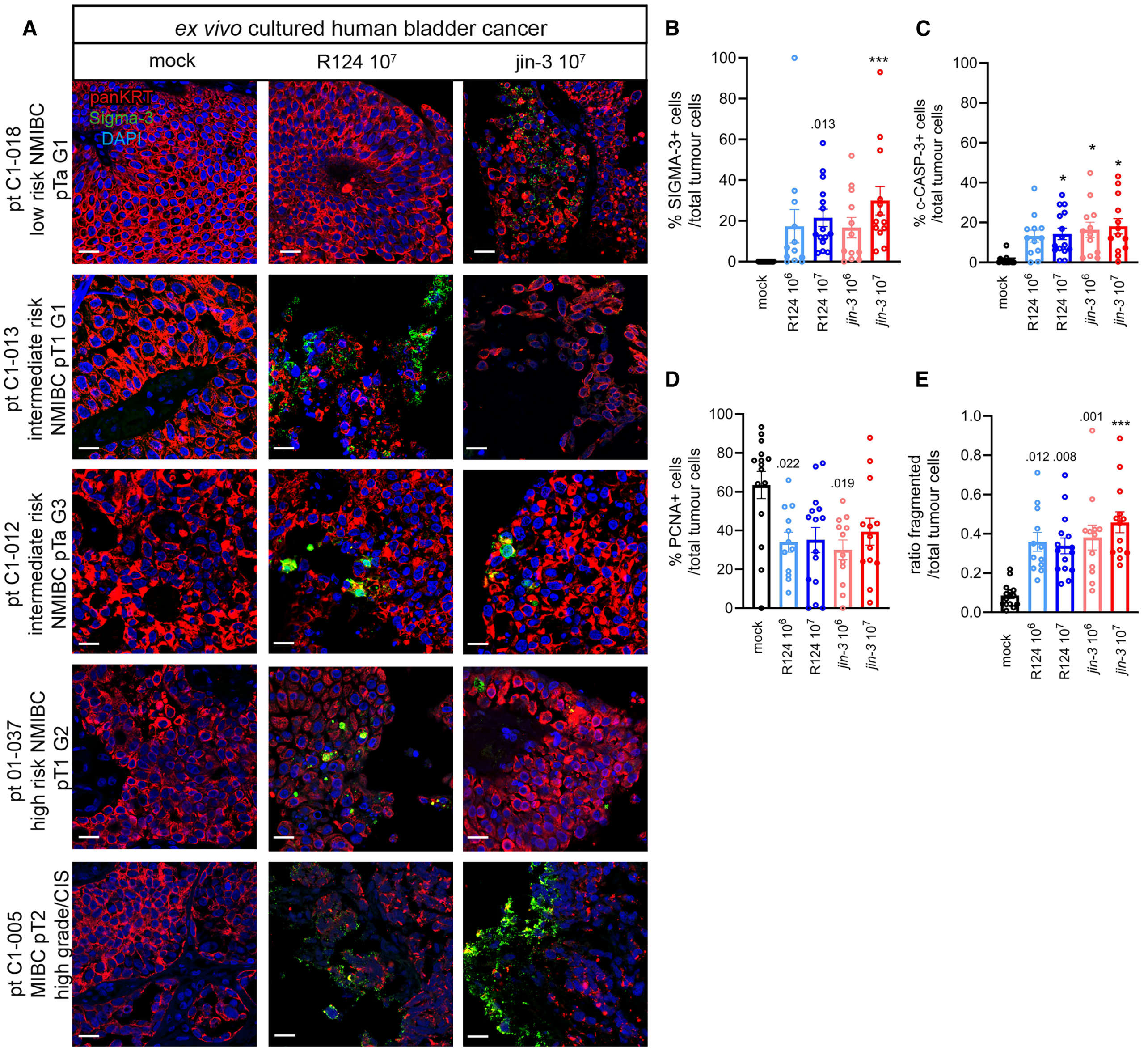

Figure 4. Comparison of *jin-3* and R124 reovirus infection in *ex vivo* cultured tumor tissue slices from human bladder cancer patients

Explanted tissue slices from patients (n = 15) diagnosed with bladder cancer were exposed to either mock, R124, or *jin-3* reovirus for 3 days and stained for viral protein, c-CASP-3, PCNA (proliferation), and integrity of the cell (nuclear DAPI and panKRT tumor marker). (A–E) Representative confocal images of the mock and 10^7 PFU condition of both viruses from five patients ranging from low-risk NMIBC to MIBC (viral protein [green, Sigma-3], tumor markers [red, panKRT], and nuclei [DAPI, blue]. Scale bars, 20 μm). Cells expressing the respective proteins [(B) Sigma-3, (C) c-CASP3, or (D) nuclear PCNA] were counted with ImageJ and divided by the number of panKRT+_DAPI+ cells. At least four fields were scored per technical replicate. Mean (SD) (3–4 replicates) with each dot representing one bladder cancer patient. The ratio of fragmented tumor cells was measured by the number of fragmented cells/total tumor cells (E). ***p < 0.001, reovirus infection versus mock, one-way ANOVA followed by Tukey' post hoc comparison.

results are in line with previous reports in prostate cancer cells showing that intra-tumoral injection of wild-type reovirus induced pro-inflammatory cytokines production.[45,64]

Intravesical administration of reovirus in an orthotopic model of superficial bladder cancer revealed minor side effects of therapy with significantly higher tumor-free survival compared to BCG treatment.[65] Moreover, in a syngeneic mouse model, intravesical therapy of reovirus in combination with an anti-programmed cell death protein 1 (PD-1) antibody improved survival and induced a more immune-active (hot) tumor microenvironment.[66]

Previous clinical studies have also demonstrated the potential of oncolytic viruses in cancer therapy, highlighting their ability to target

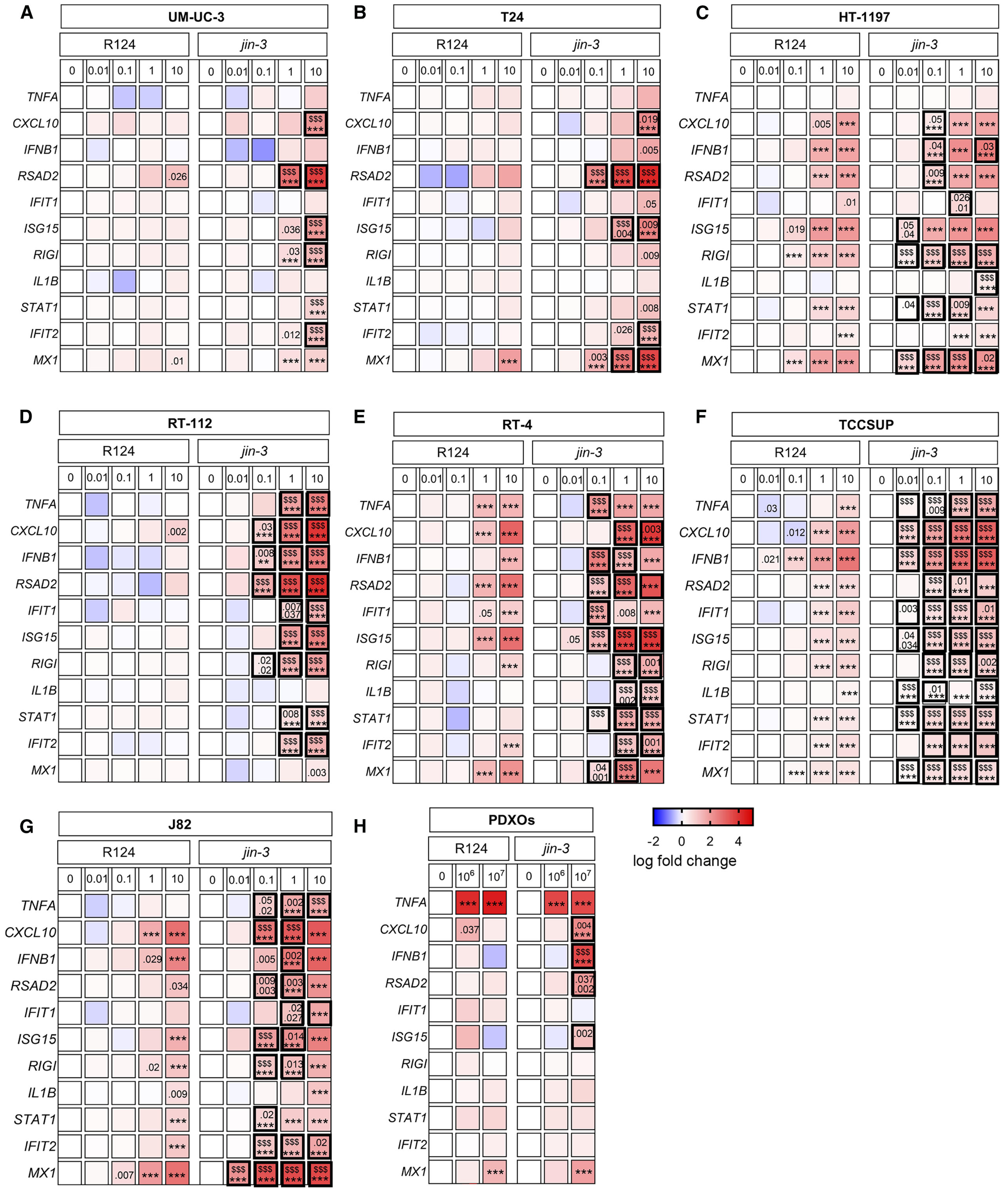

(legend on next page)

and destroy cancer cells selectively. For example, T-VEC is a Food and Drug Administration (FDA)-approved herpes-simplex-virus-based therapy for advanced melanoma.[67] Other oncolytic viruses including vesicular stomatitis virus, adenovirus, and vaccinia virus have been studied in multiple cancer types, including bladder cancer.[68–76]

Some oncolytic viruses, for example, ICAM-1 targeted Coxsackievirus A21 (CVA21) and vaccinia virus, have shown acceptable safety profiles and proof of viral targeting and replication in the bladder tumor cells in clinical trials.[68,71,77] In addition, in early stage bladder cancer, oncolytic adenoviruses have been efficacious in BCG-refractory patients.[78–85]

Importantly, clinical trials have revealed that pelareorep, also known as Reolysin, an isolate of wild-type T3 Dearing reovirus, can be safely administered to cancer patients, including patients with advanced cancer such as multiple melanoma, prostate, and esophagus cancer, with relatively few adverse effects.[43,49,86–89] However, the clinical benefit of wild-type reovirus administration is limited, and phase II studies have revealed no survival benefit.[90] No clinical trials with reovirus have been reported for bladder cancer yet. Clinical benefit may be enhanced by using our reovirus mutant *jin-3* with an expanded tropism, beyond the canonical reovirus entry receptor JAM-A.[44]

Due to the significant variability between and within tumors—including genetic mutations and differences in the tumor microenvironment—it is essential to develop diverse and improved oncolytic virotherapy strategies using multiple types of oncolytic viruses.

Matching the most effective oncolytic virus(es) to an individual patient is key for virotherapy to become more effective. Stratification of patients who are more likely to respond to a specific oncolytic virus is required but—to date—not yet accomplished. If proven, this personalized approach will enhance treatment outcomes by matching the best candidate virus to an individual tumor based on its unique biological characteristics.

Accumulating evidence shows that oncolytic virotherapy, when used as a monotherapy, is unlikely to fully overcome the immunosuppressive tumor microenvironment in a clinical setting. Several clinical studies are with oncolytic reovirus in combination with immune checkpoint inhibition in various cancers including pancreatic adenocarcinoma, metastatic non-small lung cancer, multiple myeloma, and metastatic colorectal cancer.[91–97] For example, in the GOBLET multi-cohort phase 1/2 study in pancreatic and anal cancer, intravenous pelareorep, in combination with the PD-L1 inhibitor atezolizumab, displayed promising efficacy (respectively 62% and 37.5% objective response rate for metastatic pancreatic and anal cancer).[98] Furthermore, combination of pelareorep with the PD-1 inhibitor pembrolizumab modestly promoted anti-tumor activity in advanced pancreatic cancer.[99]

To better understand the interactions between oncolytic viruses and the immune system, tumor-immune cell co-culture systems—as the ones described here—can be exploited. By incorporating both tumor cells and relevant immune cell populations (e.g., T cell populations and dendritic cells), these systems can be used to assess how different OVs modulate immune cell activation, suppression, or recruitment, to monitor cytokine and chemokine production in response to viral infection and to evaluate the potential for synergy or antagonism when combining oncolytic viruses with current immunotherapy (immune checkpoint inhibition or cell-based immunotherapy). Correct timing, optimal oncolytic virus (hence the repertoire of OVs), optimal dosing, and treatment combinations are key.

Collectively, our findings underscore the oncolytic potential of reoviruses in diverse preclinical models of human bladder cancer. Notably, the mutant reovirus *jin-3* exhibits a more robust immunomodulatory response in preclinical bladder cancer models compared to the wild-type reovirus R124. This promising result positions *jin-3* as a compelling candidate for future clinical translation.

MATERIAL AND METHODS

Reovirus production

Wild-type T3D reovirus strain R124 was plaque-purified from the wild-type reovirus T3D (ATCC) on HER911 cells.[44,100] Reovirus mutant *jin-3* was isolated from JAM-A-deficient U118MG cells after passaging of the wild-type T3D strain R124.[44] Both R124 and *jin-3* reoviruses were propagated, purified, and titrated on human HER911 cells as previously described.[44]

Preclinical models and culture conditions

Cell lines as monolayers: a panel of human bladder cancer cell lines was cultured in monolayers with the respective culture medium (Table S1).

Three-dimensional bladder cancer tumoroids or patient-derived xenograft (PDX) organoids (PDXOs) were cultured from cells isolated from subcutaneously growing PDX TM00024 tumors[51] (Jackson Laboratory; ethical permit AVD16605) or from the RT-112 and J82 cell lines as described previously.[52]

Three-dimensional tumor-immune cell co-cultures: 10,000 RT-112 cells were seeded in 40 μL domes in 60% Matrigel. After 3 days, partially

Figure 5. Comparison of immune modulation induced by *jin-3* and R124 reovirus treatment of bladder cancer cell lines

Heat maps of various inflammatory cytokines and interferon-stimulated genes (log fold change mRNA expression [$2^{-\Delta\Delta Ct}$] vs. mock treated cells) in bladder cancer cell lines UM-UC-3 (A), T24 (B), HT-1197 (C), RT-112 (D), RT-4 (E), TCCSUP (F), J82 (G), and PDXOs (H) exposed to a range of either R124 or *jin-3* reoviruses after respectively 24 h (cell lines) and 3 days (PDXOs). *p* values are depicted (vs. mock; when 2 depicted upper *p* value is R124 vs. *jin-3*) ****p* < .001, $$$ *p* < .001, asterisks indicate mock versus reovirus infection, and dollar signs indicate R124 versus *jin-3*.

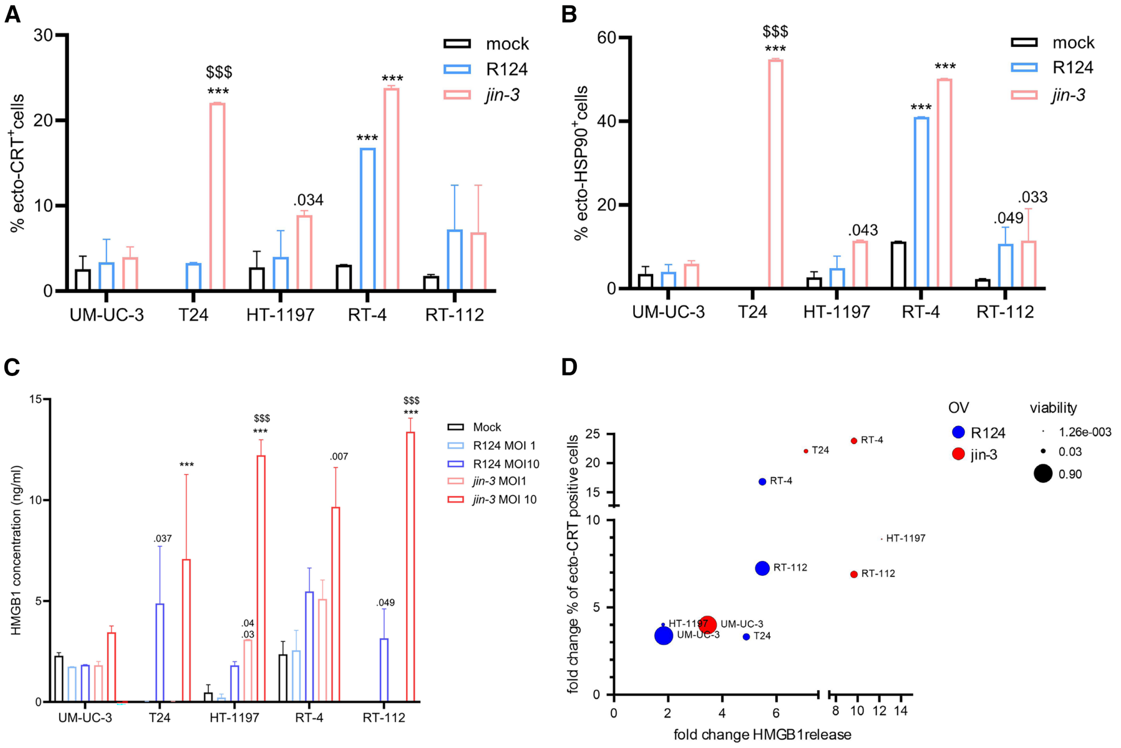

Figure 6. Comparison of immunogenic cell death induced by *jin-3* and R124 reovirus treatment of bladder cancer cell lines

(A–C) Cell lines were treated with oncolytic viruses for 48 h at an MOI of 10, and the DAMPs ecto-calreticulin (A) and ecto-HSP90 (B) were measured using flow cytometry. Secreted HMGB1 protein was measured with an ELISA after 48 h of treatment with a dose range of oncolytic viruses (C). ***$p < 0.001$, $$$ $p < 0.001$, asterisks indicate reovirus infection versus mock same day, and dollar signs indicate R124 vs. *jin-3.* Mean (SD), $N = 3$ (2 replicates). Two-way ANOVA followed by Tukey's posthoc comparison. (D) Correlation graph of the HMGB1 release (*x* axis), percentage of viable cells (size of the dots), and fold change of ecto-CRT (*y* axis)-positive cells at MOI 10 of the indicated virus.

HLA-matched PBMCs (Sanquin NVT0631.01) were added at a 20:1 effector target ratio to the 3D cultures. After 3 days, conditioned medium was collected, and RNA was isolated as described below.

Ex vivo cultured tissue explants were sliced and cultured as previously described.[54] Bladder cancer tissue was derived from either CDX, PDX, or directly from patient tumor material (Biobank Urology protocol B20.060, animal ethical protocol AVD16605; Table 1). Multiple tissue slices were used per condition and cultured for 4 days.

Viability assays

Monolayer experiments: 1,500 cells per well were seeded in a 96-well plates. After 24 h, these cells were exposed to a range of either R124 or *jin-3* (MOI 0–100 plaque-forming units [PFUs]/cell). Cells were incubated for 72 or 144 h, after which a CellTiter 96 AQueous One Solution Cell Proliferation Assay (Promega) was performed according to manufacturer's protocol. After 2 h of incubation time, optical density was measured at 490 nm using SpectraMax iD3 plate reader to assess cell viability.[45]

The 3D tumoroids or PDXOs were exposed to a range of R124 and *jin-3* reoviruses. After 3–7 days, viability was determined using the Cell Titer Glo assay, according to the manufacturer's protocol (Promega).[45]

Flow cytometry

Four hundred thousand cells/well were seeded in a 6-well plate. Each cell line was exposed to either R124 or *jin-3* (mock solution only, MOI 10) and incubated for 48 h. Subsequently, levels of viral protein expression (Sigma-3), apoptosis (cleaved caspase-3 [c-CASP-3]), and protein expression of several DAMPs (cell surface expression of calreticulin and HSP90) for each cell line/virus combination were acquired using LSR Fortessa X-20 Cell Analyzer and analyzed using FlowJo software (Table S1).

Histology and immunofluorescence

H&E and immunofluorescent stainings were executed as previously described.[54] H&E and immunofluorescent stainings were visualized using the Pannoramic MIDI slide scanner (3DHISTECH). All fluorescent stainings were visualized by confocal microscopy (63× magnification, resolution 1,024 × 1,024) (Leica SP8). At least four fields of the fluorescent staining were scored per TS by two independent reviewers. The average of these 4 fields was shown for each of the technical replicates. Cells expressing the respective proteins

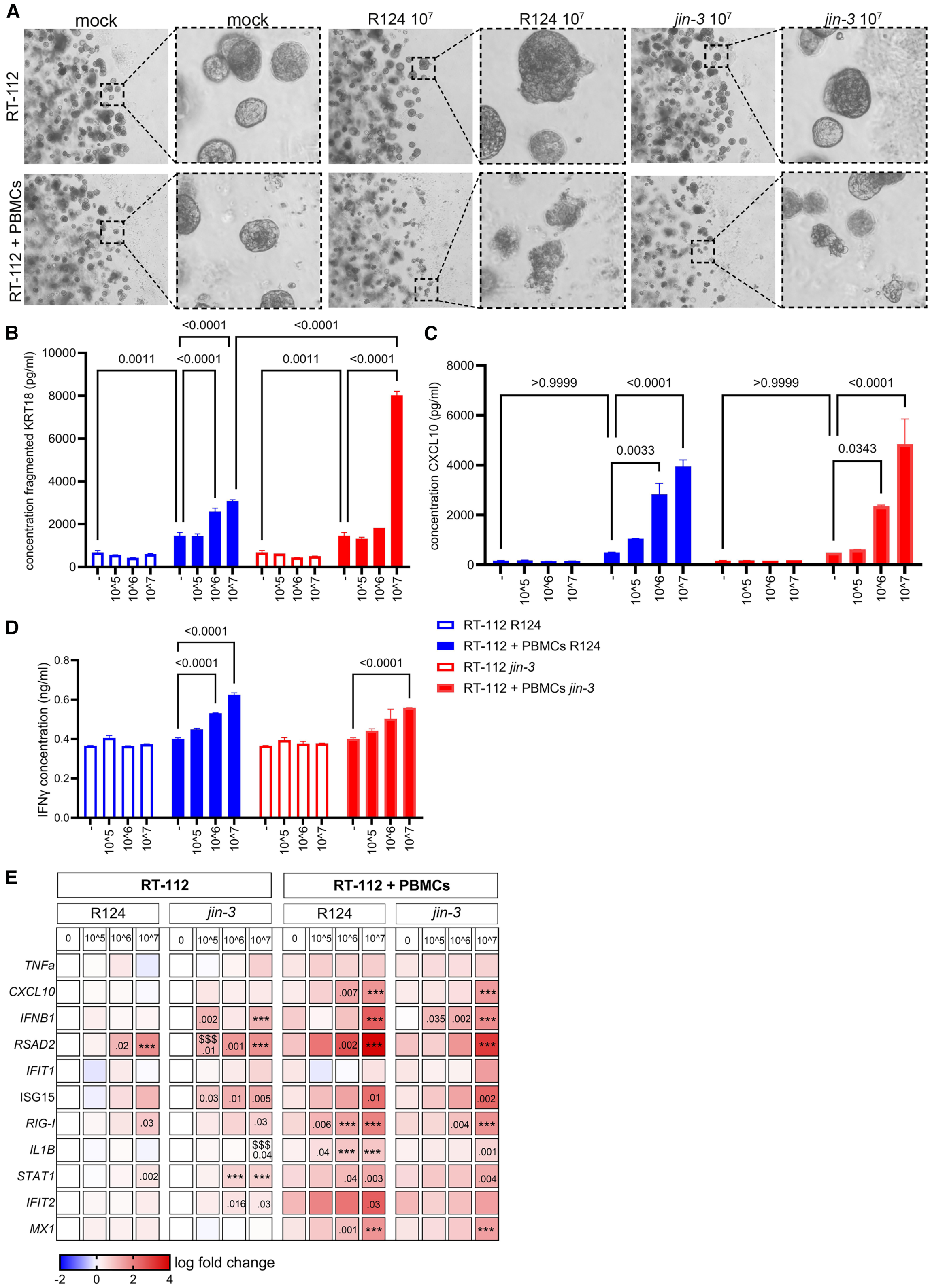

(legend on next page)

were counted with ImageJ and divided by the number of pancytokeratin-positive and DAPI-positive (panKRT+_DAPI+) cells (i.e., intact tumor cells with intact nuclei). The ratio of fragmented tumor cells was measured by the number of fragmented cells divided by the number of total tumor cells.

Real-time quantitative polymerase chain reaction

Cells were infected with the viruses and after the indicated time RNA was isolated according to the manufacturer's protocol (Nucleospin RNA kit Macherey-Nagel). cDNA was generated by using random primers (Promega), and real-time qPCR was performed with GoTaq Mastermix (Promega) according to the manufacturer's protocol in technical duplicates and biological triplicates (Promega). Gene expression was normalized to GAPDH. Primer sequences can be found in Table S1.

ELISAs

Cells were exposed to R124 or *jin-3* with an MOI of 10. At the indicated time post exposure, HMGB1 (high mobility group box 1) release was measured by performing the Lumit HMGB1 Human/Mouse Immunoassay (Promega).

Three-dimensional (co-) cultures were exposed to R124 or *jin-3*. At the indicated time points post exposure, conditioned medium was collected, and either fragmented KRT18 (Abcam), IFN-γ (Abcam), or CXCL10 (Invitrogen) levels were measured as indicated by the manufacturers protocol.

Statistical analysis

Statistical analyses were performed by using GraphPad Prism 8.0. Two-way ANOVA was performed for viability and ELISA experiments followed by Tukey post hoc test for multiple comparison. For *ex vivo* cultures, sample size was calculated using proportional odds models as described.[101] Multiple slices were scored per condition per patient (*$p < 0.05$; **$p < 0.01$; ***$p < 0.001$).

DATA AND CODE AVAILABILITY

Data supporting the findings of this study are available within the paper and its supplemental information.

ACKNOWLEDGMENTS

This study was supported by the OAK Foundation (www.supportcasper.nl;STRATIVIR and SOAK18.01), the KWF Dutch Cancer Society (KWF15932), and a kind gift from the Franje Foundation.

AUTHOR CONTRIBUTIONS

Conceptualization, R.H., G.P., and G.H.; data curation, A.F., M.M., and G.H.; formal analysis, A.F., M.M., L.H., D.W., and G.H.; funding acquisition, R.H., G.P., and G.H.; investigation, A.F., M.M., L.H., D.W., and G.H.; methodology, A.F., M.M., D.W., and G.H.; project administration, G.P. and G.H.; resources, R.H., D.W., R.F., and W.Z.; supervision, R.H., G.P., and G.H.; validation, A.F., M.M., G.P., and G.H.; visualization, A.F., M.M., and G.H.; writing—original draft, A.F. and G.H.; writing—review and editing, A.F., R.H., M.K., G.P., and G.H.

DECLARATION OF INTERESTS

The authors declare no competing interests.

SUPPLEMENTAL INFORMATION

Supplemental information can be found online at https://doi.org/10.1016/j.omton.2026.201128.

REFERENCES

1. Wéber, A., Vignat, J., Shah, R., Morgan, E., Laversanne, M., Nagy, P., Kenessey, I., and Znaor, A. (2024). Global burden of bladder cancer mortality in 2020 and 2040 according to GLOBOCAN estimates. World J. Urol. *42*, 237.
2. Babjuk, M., Burger, M., Compérat, E.M., Gontero, P., Mostafid, A.H., Palou, J., van Rhijn, B.W.G., Rouprêt, M., Shariat, S.F., Sylvester, R., et al. (2019). European Association of Urology Guidelines on Non-muscle-invasive Bladder Cancer (TaT1 and Carcinoma In Situ) - 2019 Update. Eur. Urol. *76*, 639–657.
3. Zhang, Y., Zhang, X., Huang, X., Tang, X., Zhang, M., Li, Z., Hu, X., Zhang, M., Wang, X., and Yan, Y. (2023). Tumor stemness score to estimate epithelial-to-mesenchymal transition (EMT) and cancer stem cells (CSCs) characterization and to predict the prognosis and immunotherapy response in bladder urothelial carcinoma. Stem Cell Res. Ther. *14*, 15.
4. Schmidt, S., Kunath, F., Coles, B., Draeger, D.L., Krabbe, L.M., Dersch, R., Kilian, S., Jensen, K., Dahm, P., and Meerpohl, J.J. (2020). Intravesical Bacillus Calmette-Guérin versus mitomycin C for Ta and T1 bladder cancer: Abridged summary of the Cochrane Review. Investig. Clin. Urol. *61*, 349–354.
5. Chan, K.S., Volkmer, J.P., and Weissman, I. (2010). Cancer stem cells in bladder cancer: a revisited and evolving concept. Curr. Opin. Urol. *20*, 393–397.
6. Chang, J.T., and Mani, S.A. (2013). Sheep, wolf, or werewolf: cancer stem cells and the epithelial-to-mesenchymal transition. Cancer Lett. *341*, 16–23.
7. Ferlay, J., Steliarova-Foucher, E., Lortet-Tieulent, J., Rosso, S., Coebergh, J.W.W., Comber, H., Forman, D., and Bray, F. (2013). Cancer incidence and mortality patterns in Europe: estimates for 40 countries in 2012. Eur. J. Cancer *49*, 1374–1403.
8. Dyba, T., Randi, G., Bray, F., Martos, C., Giusti, F., Nicholson, N., Gavin, A., Flego, M., Neamtiu, L., Dimitrova, N., et al. (2021). The European cancer burden in 2020: Incidence and mortality estimates for 40 countries and 25 major cancers. Eur. J. Cancer *157*, 308–347.
9. Chalmers, Z.R., Connelly, C.F., Fabrizio, D., Gay, L., Ali, S.M., Ennis, R., Schrock, A., Campbell, B., Shlien, A., Chmielecki, J., et al. (2017). Analysis of 100,000 human cancer genomes reveals the landscape of tumor mutational burden. Genome Med. *9*, 34.
10. Yarchoan, M., Hopkins, A., and Jaffee, E.M. (2017). Tumor Mutational Burden and Response Rate to PD-1 Inhibition. N. Engl. J. Med. *377*, 2500–2501.
11. Hussain, S.A., Birtle, A., Crabb, S., Huddart, R., Small, D., Summerhayes, M., Jones, R., and Protheroe, A. (2018). From Clinical Trials to Real-life Clinical Practice: The

Figure 7. Activation of PBMCs in RT-112 co-cultures

(A) Brightfield images of RT-112 bladder cancer cells that were cultured in 60% Matrigel and allowed to form 3D structures for approximately 3 days. Subsequently, PBMCs were added at an effector: target ratio of 20:1 in absence or presence of either R124 or *jin-3* reovirus at the indicated PFU for an additional 3 days. (B) Fragmented KRT18 was determined as an outcome measure for tumor cell killing 3 days after OV exposure. (C–E) CXCL10 levels and (D) IFN-γ levels were measured 3 days after OV exposure. ($n = 3$, 2 replicates). Mean (SD). Two-way ANOVA with Tukey's post hoc comparison. (E) Heatmap of various inflammatory cytokines and interferon-stimulated genes (log fold change mRNA expression [$2^{-\Delta\Delta Ct}$] vs. mock treated cells) in 3D cultured RT-112 cells or RT-112 cells co-cultured with PBMCs exposed to either R124 or *jin-3* reoviruses. Mean (SD). *p* values are depicted (vs. mock; when two depicted upper *p* value is R124 vs. *jin-3*) ***$p < .001$, $$$ $p < .001$, asterisks indicate mock versus reovirus infection and dollar signs R124 versus *jin-3.*, $n = 2$ (2 replicates). Two-way ANOVA followed by Tukey's post hoc comparison.

Role of Immunotherapy with PD-1/PD-L1 Inhibitors in Advanced Urothelial Carcinoma. Eur. Urol. Oncol. *1*, 486–500.

12. Hsu, M.M., and Balar, A.V. (2019). PD-1/PD-L1 Combinations in Advanced Urothelial Cancer: Rationale and Current Clinical Trials. Clin. Genitourin. Cancer *17*, e618–e626.
13. Gupta, M., Kates, M., and Bivalacqua, T.J. (2019). Immunotherapy in nonmuscle invasive bladder cancer: current and emerging treatments. Curr. Opin. Oncol. *31*, 183–187.
14. van Dijk, N., Funt, S.A., Blank, C.U., Powles, T., Rosenberg, J.E., and van der Heijden, M.S. (2019). The Cancer Immunogram as a Framework for Personalized Immunotherapy in Urothelial Cancer. Eur. Urol. *75*, 435–444.
15. Hurwitz, M.E., Sokhn, J., and Petrylak, D.P. (2016). Cancer immunotherapy: new applications in urologic oncology. Curr. Opin. Urol. *26*, 535–542.
16. Yang, R., and Yu, Y. (2023). Patient-derived organoids in translational oncology and drug screening. Cancer Lett. *562*, 216180.
17. Balar, A.V. (2017). Immune Checkpoint Blockade in Metastatic Urothelial Cancer. J. Clin. Oncol. *35*, 2109–2112.
18. Balar, A.V., Castellano, D., O'Donnell, P.H., Grivas, P., Vuky, J., Powles, T., Plimack, E.R., Hahn, N.M., de Wit, R., Pang, L., et al. (2017). First-line pembrolizumab in cisplatin-ineligible patients with locally advanced and unresectable or metastatic urothelial cancer (KEYNOTE-052): a multicentre, single-arm, phase 2 study. Lancet Oncol. *18*, 1483–1492.
19. Balar, A.V., Galsky, M.D., Rosenberg, J.E., Powles, T., Petrylak, D.P., Bellmunt, J., Loriot, Y., Necchi, A., Hoffman-Censits, J., Perez-Gracia, J.L., et al. (2017). Atezolizumab as first-line treatment in cisplatin-ineligible patients with locally advanced and metastatic urothelial carcinoma: a single-arm, multicentre, phase 2 trial. Lancet *389*, 67–76.
20. Balar, A.V., and Weber, J.S. (2017). PD-1 and PD-L1 antibodies in cancer: current status and future directions. Cancer Immunol. Immunother. *66*, 551–564.
21. Bellmunt, J., and Bajorin, D.F. (2017). Pembrolizumab for Advanced Urothelial Carcinoma. N. Engl. J. Med. *376*, 2304.
22. Bellmunt, J., de Wit, R., Vaughn, D.J., Fradet, Y., Lee, J.L., Fong, L., Vogelzang, N.J., Climent, M.A., Petrylak, D.P., Choueiri, T.K., et al. (2017). Pembrolizumab as Second-Line Therapy for Advanced Urothelial Carcinoma. N. Engl. J. Med. *376*, 1015–1026.
23. Bellmunt, J., Kerst, J.M., Vazquez, F., Morales-Barrera, R., Grande, E., Medina, A., Gonzalez Graguera, M.B., Rubio, G., Anido, U., Fernandez Calvo, O., et al. (2017). A randomized phase II/III study of cabazitaxel versus vinflunine in metastatic or locally advanced transitional cell carcinoma of the urothelium (SECAVIN). Ann. Oncol. *28*, 1517–1522.
24. Bellmunt, J., and Nadal, R. (2018). Urothelial cancer in 2017: Changes in expectations for metastatic urothelial carcinoma. Nat. Rev. Clin. Oncol. *15*, 73–74.
25. Bellmunt, J., Powles, T., and Vogelzang, N.J. (2017). A review on the evolution of PD-1/PD-L1 immunotherapy for bladder cancer: The future is now. Cancer Treat Rev. *54*, 58–67.
26. Massard, C., Gordon, M.S., Sharma, S., Rafii, S., Wainberg, Z.A., Luke, J., Curiel, T.J., Colon-Otero, G., Hamid, O., Sanborn, R.E., et al. (2016). Safety and Efficacy of Durvalumab (MEDI4736), an Anti-Programmed Cell Death Ligand-1 Immune Checkpoint Inhibitor, in Patients With Advanced Urothelial Bladder. J. Clin. Oncol. *34*, 3119–3125.
27. Sharma, P., Callahan, M.K., Bono, P., Kim, J., Spiliopoulou, P., Calvo, E., Pillai, R.N., Ott, P.A., de Braud, F., Morse, M., et al. (2016). Nivolumab monotherapy in recurrent metastatic urothelial carcinoma (CheckMate 032): a multicentre, open-label, two-stage, multi-arm, phase 1/2 trial. Lancet Oncol. *17*, 1590–1598.
28. Sari Motlagh, R., Pradere, B., Mori, K., Miura, N., Abufaraj, M., and Shariat, S.F. (2020). Bladder-preserving strategies for Bacillus Calmette-Guerin unresponsive non-muscle invasive bladder cancer; where are we and what will be expected? Curr. Opin. Urol. *30*, 584–593.
29. Balar, A.V., Kamat, A.M., Kulkarni, G.S., Uchio, E.M., Boormans, J.L., Roumiguié, M., Krieger, L.E.M., Singer, E.A., Bajorin, D.F., Grivas, P., et al. (2021). Pembrolizumab monotherapy for the treatment of high-risk non-muscle-invasive bladder cancer unresponsive to BCG (KEYNOTE-057): an open-label, single-arm, multicentre, phase 2 study. Lancet Oncol. *22*, 919–930.
30. Lopez-Beltran, A., Cimadamore, A., Blanca, A., Massari, F., Vau, N., Scarpelli, M., Cheng, L., and Montironi, R. (2021). Immune Checkpoint Inhibitors for the Treatment of Bladder Cancer. Cancers (Basel) *13*, 131.
31. Roumiguié, M., Compérat, E., Chaltiel, L., Nouhaud, F.X., Verhoest, G., Masson-Lecomte, A., Colin, P., Audenet, F., Houédé, N., Larré, S., et al. (2021). PD-L1 expression and pattern of immune cells in pre-treatment specimens are associated with disease-free survival for HR-NMIBC undergoing BCG treatment. World J. Urol. *39*, 4055–4065.
32. Ghate, K., Amir, E., Kuksis, M., Hernandez-Barajas, D., Rodriguez-Romo, L., Booth, C.M., and Vera-Badillo, F.E. (2019). PD-L1 expression and clinical outcomes in patients with advanced urothelial carcinoma treated with checkpoint inhibitors: A meta-analysis. Cancer Treat Rev. *76*, 51–56.
33. Maiorano, B.A., Di Maio, M., Cerbone, L., Maiello, E., Procopio, G., and Roviello, G.; MeetURO Group (2024). Significance of PD-L1 in Metastatic Urothelial Carcinoma Treated With Immune Checkpoint Inhibitors: A Systematic Review and Meta-Analysis. JAMA Netw. Open *7*, e241215.
34. Davola, M.E., and Mossman, K.L. (2019). Oncolytic viruses: how "lytic" must they be for therapeutic efficacy? OncoImmunology *8*, e1581528.
35. Errington, F., Steele, L., Prestwich, R., Harrington, K.J., Pandha, H.S., Vidal, L., de Bono, J., Selby, P., Coffey, M., Vile, R., et al. (2008). Reovirus activates human dendritic cells to promote innate antitumor immunity. J. Immunol. *180*, 6018–6026.
36. Lee, P., and Gujar, S. (2018). Potentiating prostate cancer immunotherapy with oncolytic viruses. Nat. Rev. Urol. *15*, 235–250.
37. Prestwich, R.J., Errington, F., Harrington, K.J., Pandha, H.S., Selby, P., and Melcher, A. (2008). Oncolytic viruses: do they have a role in anti-cancer therapy? Clin. Med. Oncol. *2*, 83–96.
38. Prestwich, R.J., Ilett, E.J., Errington, F., Diaz, R.M., Steele, L.P., Kottke, T., Thompson, J., Galivo, F., Harrington, K.J., Pandha, H.S., et al. (2009). Immune-mediated antitumor activity of reovirus is required for therapy and is independent of direct viral oncolysis and replication. Clin. Cancer Res. *15*, 4374–4381.
39. Tang, D., Kang, R., Berghe, T.V., Vandenabeele, P., and Kroemer, G. (2019). The molecular machinery of regulated cell death. Cell Res. *29*, 347–364.
40. Galluzzi, L., Vitale, I., Warren, S., Adjemian, S., Agostinis, P., Martinez, A.B., Chan, T.A., Coukos, G., Demaria, S., Deutsch, E., et al. (2020). Consensus guidelines for the definition, detection and interpretation of immunogenic cell death. J. Immunother. Cancer *8*, e000337.
41. Tesniere, A., Panaretakis, T., Kepp, O., Apetoh, L., Ghiringhelli, F., Zitvogel, L., and Kroemer, G. (2008). Molecular characteristics of immunogenic cancer cell death. Cell Death Differ. *15*, 3–12.
42. Kepp, O., Senovilla, L., Vitale, I., Vacchelli, E., Adjemian, S., Agostinis, P., Apetoh, L., Aranda, F., Barnaba, V., Bloy, N., et al. (2014). Consensus guidelines for the detection of immunogenic cell death. OncoImmunology *3*, e955691.
43. Vidal, L., Pandha, H.S., Yap, T.A., White, C.L., Twigger, K., Vile, R.G., Melcher, A., Coffey, M., Harrington, K.J., and DeBono, J.S. (2008). A phase I study of intravenous oncolytic reovirus type 3 Dearing in patients with advanced cancer. Clin. Cancer Res. *14*, 7127–7137.
44. van den Wollenberg, D.J.M., Dautzenberg, I.J.C., van den Hengel, S.K., Cramer, S.J., de Groot, R.J., and Hoeben, R.C. (2012). Isolation of reovirus T3D mutants capable of infecting human tumor cells independent of junction adhesion molecule-A. PLoS One *7*, e48064.
45. van de Merbel, A.F., van der Horst, G., van der Mark, M.H., Bots, S.T.F., van den Wollenberg, D.J.M., de Ridder, C.M.A., Stuurman, D., Aalders, T., Erkens-Schulz, S., van Montfoort, N., et al. (2021). Reovirus mutant jin-3 exhibits lytic and immune-stimulatory effects in preclinical human prostate cancer models. Cancer Gene Ther. *29*, 1–10.
46. Mitchell, L.A., Ward, C., Kwon, M., Mitchell, P.O., Quintero, D.A., Nusrat, A., Parkos, C.A., and Koval, M. (2015). Junctional adhesion molecule A promotes epithelial tight junction assembly to augment lung barrier function. Am. J. Pathol. *185*, 372–386.

47. Ren, T., Zheng, Y., Liu, F., Liu, C., Zhang, B., Ren, H., Gao, X., Wei, Y., Sun, Q., and Huang, H. (2024). Identification and Validation of JAM-A as a Novel Prognostic and Immune Factor in Human Tumors. Biomedicines *12*, 1423.
48. Zuiverloon, T.C.M., de Jong, F.C., Costello, J.C., and Theodorescu, D. (2018). Systematic Review: Characteristics and Preclinical Uses of Bladder Cancer Cell Lines. Bladder Cancer *4*, 169–183.
49. Thirukkumaran, C.M., Nodwell, M.J., Hirasawa, K., Shi, Z.Q., Diaz, R., Luider, J., Johnston, R.N., Forsyth, P.A., Magliocco, A.M., Lee, P., et al. (2010). Oncolytic viral therapy for prostate cancer: efficacy of reovirus as a biological therapeutic. Cancer Res. *70*, 2435–2444.
50. Barton, E.S., Forrest, J.C., Connolly, J.L., Chappell, J.D., Liu, Y., Schnell, F.J., Nusrat, A., Parkos, C.A., and Dermody, T.S. (2001). Junction adhesion molecule is a receptor for reovirus. Cell *104*, 441–451.
51. Pan, C.X., Zhang, H., Tepper, C.G., Lin, T.Y., Davis, R.R., Keck, J., Ghosh, P.M., Gill, P., Airhart, S., Bult, C., et al. (2015). Development and Characterization of Bladder Cancer Patient-Derived Xenografts for Molecularly Guided Targeted Therapy. PLoS One *10*, e0134346.
52. Mullenders, J., de Jongh, E., Brousali, A., Roosen, M., Blom, J.P.A., Begthel, H., Korving, J., Jonges, T., Kranenburg, O., Meijer, R., et al. (2019). Mouse and human urothelial cancer organoids: A tool for bladder cancer research. Proc. Natl. Acad. Sci. USA *116*, 4567–4574.
53. Lee, S.H., Hu, W., Matulay, J.T., Silva, M.V., Owczarek, T.B., Kim, K., Chua, C.W., Barlow, L.J., Kandoth, C., Williams, A.B., et al. (2018). Tumor Evolution and Drug Response in Patient-Derived Organoid Models of Bladder Cancer. Cell *173*, 515–528.e17.
54. van de Merbel, A.F., van der Horst, G., van der Mark, M.H., van Uhm, J.I.M., van Gennep, E.J., Kloen, P., Beimers, L., Pelger, R.C.M., and van der Pluijm, G. (2018). An ex vivo Tissue Culture Model for the Assessment of Individualized Drug Responses in Prostate and Bladder Cancer. Front. Oncol. *8*, 400.
55. Vitiello, G.A.F., Ferreira, W.A.S., Cordeiro de Lima, V.C., and Medina, T.D.S. (2021). Antiviral Responses in Cancer: Boosting Antitumor Immunity Through Activation of Interferon Pathway in the Tumor Microenvironment. Front. Immunol. *12*, 782852.
56. Matveeva, O.V., and Chumakov, P.M. (2018). Defects in interferon pathways as potential biomarkers of sensitivity to oncolytic viruses. Rev. Med. Virol. *28*, e2008.
57. Kurokawa, C., Iankov, I.D., Anderson, S.K., Aderca, I., Leontovich, A.A., Maurer, M.J., Oberg, A.L., Schroeder, M.A., Giannini, C., Greiner, S.M., et al. (2018). Constitutive Interferon Pathway Activation in Tumors as an Efficacy Determinant Following Oncolytic Virotherapy. J. Natl. Cancer Inst. *110*, 1123–1132.
58. Knowles, M.A., and Hurst, C.D. (2015). Molecular biology of bladder cancer: new insights into pathogenesis and clinical diversity. Nat. Rev. Cancer *15*, 25–41.
59. Lawson, A.R.J., Abascal, F., Coorens, T.H.H., Hooks, Y., O'Neill, L., Latimer, C., Raine, K., Sanders, M.A., Warren, A.Y., Mahbubani, K.T.A., et al. (2020). Extensive heterogeneity in somatic mutation and selection in the human bladder. Science *370*, 75–82.
60. Lemos de Matos, A., Franco, L.S., and McFadden, G. (2020). Oncolytic Viruses and the Immune System: The Dynamic Duo. Mol. Ther. Methods Clin. Dev. *17*, 349–358.
61. Li, R., Zhang, J., Gilbert, S.M., Conejo-Garcia, J., and Mulé, J.J. (2021). Using oncolytic viruses to ignite the tumour immune microenvironment in bladder cancer. Nat. Rev. Urol. *18*, 543–555.
62. Kaufman, H.L., Kohlhapp, F.J., and Zloza, A. (2015). Oncolytic viruses: a new class of immunotherapy drugs. Nat. Rev. Drug Discov. *14*, 642–662.
63. Jeong, S.R., and Kang, M. (2023). Exploring Tumor-Immune Interactions in Co-Culture Models of T Cells and Tumor Organoids Derived from Patients. Int. J. Mol. Sci. *24*, 14609.
64. Gujar, S.A., Pan, D.A., Marcato, P., Garant, K.A., and Lee, P.W.K. (2011). Oncolytic virus-initiated protective immunity against prostate cancer. Mol. Ther. *19*, 797–804.
65. Hanel, E.G., Xiao, Z., Wong, K.K., Lee, P.W.K., Britten, R.A., and Moore, R.B. (2004). A novel intravesical therapy for superficial bladder cancer in an orthotopic model: oncolytic reovirus therapy. J. Urol. *172*, 2018–2022.
66. Smelser, W.W., Wang, J., Ogden, K.M., Chang, S.S., and Kirschner, A.N. (2023). Intravesical oncolytic virotherapy and immunotherapy for non-muscle-invasive bladder cancer mouse model. BJU Int. *132*, 298–306.
67. Zhang, T., Jou, T.H.T., Hsin, J., Wang, Z., Huang, K., Ye, J., Yin, H., and Xing, Y. (2023). Talimogene Laherparepvec (T-VEC): A Review of the Recent Advances in Cancer Therapy. J. Clin. Med. *12*, 1098.
68. Taguchi, S., Fukuhara, H., Homma, Y., and Todo, T. (2017). Current status of clinical trials assessing oncolytic virus therapy for urological cancers. Int. J. Urol. *24*, 342–351.
69. Cozzi, P.J., Malhotra, S., McAuliffe, P., Kooby, D.A., Federoff, H.J., Huryk, B., Johnson, P., Scardino, P.T., Heston, W.D., and Fong, Y. (2001). Intravesical oncolytic viral therapy using attenuated, replication-competent herpes simplex viruses G207 and Nv1020 is effective in the treatment of bladder cancer in an orthotopic syngeneic model. FASEB J. *15*, 1306–1308.
70. Hadaschik, B.A., Zhang, K., So, A.I., Fazli, L., Jia, W., Bell, J.C., Gleave, M.E., and Rennie, P.S. (2008). Oncolytic vesicular stomatitis viruses are potent agents for intravesical treatment of high-risk bladder cancer. Cancer Res. *68*, 4506–4510.
71. Annels, N.E., Mansfield, D., Arif, M., Ballesteros-Merino, C., Simpson, G.R., Denyer, M., Sandhu, S.S., Melcher, A.A., Harrington, K.J., Davies, B., et al. (2019). Phase I Trial of an ICAM-1-Targeted Immunotherapeutic-Coxsackievirus A21 (CVA21) as an Oncolytic Agent Against Non Muscle-Invasive Bladder Cancer. Clin. Cancer Res. *25*, 5818–5831.
72. Shalhout, S.Z., Miller, D.M., Emerick, K.S., and Kaufman, H.L. (2023). Therapy with oncolytic viruses: progress and challenges. Nat. Rev. Clin. Oncol. *20*, 160–177.
73. Engeland, C.E., and Bell, J.C. (2020). Introduction to Oncolytic Virotherapy. Methods Mol. Biol. *2058*, 1–6.
74. Li, M., Zhang, M., Ye, Q., Liu, Y., and Qian, W. (2023). Preclinical and clinical trials of oncolytic vaccinia virus in cancer immunotherapy: a comprehensive review. Cancer Biol. Med. *20*, 646–661.
75. Muhlebach, M.D. (2020). Measles virus in cancer therapy. Curr. Opin. Virol. *41*, 85–97.
76. Javanbakht, M., Tahmasebzadeh, S., Cegolon, L., Gholami, N., Kashaki, M., Nikoueinejad, H., Mozafari, M., Mozaffari, M., Zhao, S., Khafaei, M., et al. (2023). Oncolytic viruses: A novel treatment strategy for breast cancer. Genes Dis. *10*, 430–446.
77. Annels, N.E., Arif, M., Simpson, G.R., Denyer, M., Moller-Levet, C., Mansfield, D., Butler, R., Shafren, D., Au, G., Knowles, M., et al. (2018). Oncolytic Immunotherapy for Bladder Cancer Using Coxsackie A21 Virus. Mol. Ther. Oncolytics *9*, 1–12.
78. Burke, J. (2010). Virus therapy for bladder cancer. Cytokine Growth Factor Rev. *21*, 99–102.
79. Fodor, I., Timiryasova, T., Denes, B., Yoshida, J., Ruckle, H., and Lilly, M. (2005). Vaccinia virus mediated p53 gene therapy for bladder cancer in an orthotopic murine model. J. Urol. *173*, 604–609.
80. Kuball, J., Wen, S.F., Leissner, J., Atkins, D., Meinhardt, P., Quijano, E., Engler, H., Hutchins, B., Maneval, D.C., Grace, M.J., et al. (2002). Successful adenovirus-mediated wild-type p53 gene transfer in patients with bladder cancer by intravesical vector instillation. J. Clin. Oncol. *20*, 957–965.
81. Freund, C.T., Tong, X.W., Block, A., Contant, C.F., Kieback, D.G., Rowley, D.R., and Lerner, S.P. (2000). Adenovirus-mediated suicide gene therapy for bladder cancer: comparison of the cytomegalovirus- and Rous sarcoma virus-promoter. Anticancer Res. *20*, 2811–2816.
82. Gomella, L.G., Mastrangelo, M.J., McCue, P.A., Maguire HC, J.R., Mulholland, S.G., and Lattime, E.C. (2001). Phase i study of intravesical vaccinia virus as a vector for gene therapy of bladder cancer. J. Urol. *166*, 1291–1295.
83. Hu, C., Liu, Y., Lin, Y., Liang, J.K., Zhong, W.W., Li, K., Huang, W.T., Wang, D.J., Yan, G.M., Zhu, W.B., et al. (2018). Intravenous injections of the oncolytic virus M1 as a novel therapy for muscle-invasive bladder cancer. Cell Death Dis. *9*, 274.
84. Kohno, S.i., Luo, C., Goshima, F., Nishiyama, Y., Sata, T., and Ono, Y. (2005). Herpes simplex virus type 1 mutant HF10 oncolytic viral therapy for bladder cancer. Urology *66*, 1116–1121.

85. Shiau, A.L., Lin, Y.P., Shieh, G.S., Su, C.H., Wu, W.L., Tsai, Y.S., Cheng, C.W., Lai, M.D., and Wu, C.L. (2007). Development of a conditionally replicating pseudorabies virus for HER-2/neu-overexpressing bladder cancer therapy. Mol. Ther. *15*, 131–138.

86. Kemp, V., Lamfers, M.L.M., van der Pluijm, G., van den Hoogen, B.G., and Hoeben, R.C. (2020). Developing oncolytic viruses for clinical use: A consortium approach. Cytokine Growth Factor Rev. *56*, 133–140.

87. Gujar, S., Bell, J., and Diallo, J.S. (2019). SnapShot: Cancer Immunotherapy with Oncolytic Viruses. Cell *176*, 1240–1240.e1.

88. Comins, C., Spicer, J., Protheroe, A., Roulstone, V., Twigger, K., White, C.M., Vile, R., Melcher, A., Coffey, M.C., Mettinger, K.L., et al. (2010). REO-10: a phase I study of intravenous reovirus and docetaxel in patients with advanced cancer. Clin. Cancer Res. *16*, 5564–5572.

89. Kicielinski, K.P., Chiocca, E.A., Yu, J.S., Gill, G.M., Coffey, M., and Markert, J.M. (2014). Phase 1 clinical trial of intratumoral reovirus infusion for the treatment of recurrent malignant gliomas in adults. Mol. Ther. *22*, 1056–1062.

90. Eigl, B.J., Chi, K., Tu, D., Hotte, S.J., Winquist, E., Booth, C.M., Canil, C., Potvin, K., Gregg, R., North, S., et al. (2018). A randomized phase II study of pelareorep and docetaxel or docetaxel alone in men with metastatic castration resistant prostate cancer: CCTG study IND 209. Oncotarget *9*, 8155–8164.

91. Bradbury, P.A., Morris, D.G., Nicholas, G., Tu, D., Tehfe, M., Goffin, J.R., Shepherd, F.A., Gregg, R.W., Rothenstein, J., Lee, C., et al. (2018). Canadian Cancer Trials Group (CCTG) IND211: A randomized trial of pelareorep (Reolysin) in patients with previously treated advanced or metastatic non-small cell lung cancer receiving standard salvage therapy. Lung Cancer *120*, 142–148.

92. Clark, A.S., Zhao, F., Klein, P., Montero, A.J., Falkson, C., Krill-Jackson, E., Rowland, K., Sardesai, S., Incorvati, J., Dillon, P., et al. (2025). A Phase II Randomized Study of Paclitaxel Alone or Combined with Pelareorep with or without Avelumab in Metastatic Hormone Receptor-Positive Breast Cancer: The BRACELET-01/PrE0113 Study. Clin. Cancer Res. *31*, 2655–2662.

93. Goel, S., Ocean, A.J., Parakrama, R.Y., Ghalib, M.H., Chaudhary, I., Shah, U., Viswanathan, S., Kharkwal, H., Coffey, M., and Maitra, R. (2020). Elucidation of Pelareorep Pharmacodynamics in A Phase I Trial in Patients with KRAS-Mutated Colorectal Cancer. Mol. Cancer Ther. *19*, 1148–1156.

94. Mahalingam, D., Fountzilas, C., Moseley, J., Noronha, N., Tran, H., Chakrabarty, R., Selvaggi, G., Coffey, M., Thompson, B., and Sarantopoulos, J. (2017). A phase II study of REOLYSIN(®) (pelareorep) in combination with carboplatin and paclitaxel for patients with advanced malignant melanoma. Cancer Chemother. Pharmacol. *79*, 697–703.

95. Mahalingam, D., Wilkinson, G.A., Eng, K.H., Fields, P., Raber, P., Moseley, J.L., Cheetham, K., Coffey, M., Nuovo, G., Kalinski, P., et al. (2020). Pembrolizumab in Combination with the Oncolytic Virus Pelareorep and Chemotherapy in Patients with Advanced Pancreatic Adenocarcinoma: A Phase Ib Study. Clin. Cancer Res. *26*, 71–81.

96. Nawrocki, S.T., Olea, J., Villa Celi, C., Dadrastoussi, H., Wu, K., Tsao-Wei, D., Colombo, A., Coffey, M., Fernandez Hernandez, E., Chen, X., et al. (2023). Comprehensive Single-Cell Immune Profiling Defines the Patient Multiple Myeloma Microenvironment Following Oncolytic Virus Therapy in a Phase Ib Trial. Clin. Cancer Res. *29*, 5087–5103.

97. Parakrama, R., Fogel, E., Chandy, C., Augustine, T., Coffey, M., Tesfa, L., Goel, S., and Maitra, R. (2020). Immune characterization of metastatic colorectal cancer patients post reovirus administration. BMC Cancer *20*, 569.

98. Collienne, M., Loghmani, H., Heineman, T.C., and Arnold, D. (2022). GOBLET: a phase I/II study of pelareorep and atezolizumab +/- chemo in advanced or metastatic gastrointestinal cancers. Future Oncol. *18*, 2871–2878.

99. Mahalingam, D., Chen, S., Xie, P., Loghmani, H., Heineman, T., Kalyan, A., Kircher, S., Helenowski, I.B., Mi, X., Maurer, V., et al. (2023). Combination of pembrolizumab and pelareorep promotes anti-tumour immunity in advanced pancreatic adenocarcinoma (PDAC). Br. J. Cancer *129*, 782–790.

100. Dautzenberg, I.J.C., van den Wollenberg, D.J.M., van den Hengel, S.K., Limpens, R.W.A., Bárcena, M., Koster, A.J., and Hoeben, R.C. (2014). Mammalian orthoreovirus T3D infects U-118 MG cell spheroids independent of junction adhesion molecule-A. Gene Ther. *21*, 609–617.

101. Walters, S.J. (2004). Sample size and power estimation for studies with health related quality of life outcomes: a comparison of four methods using the SF-36. Health Qual. Life Outcomes *2*, 26.

OMTON, Volume *34*

Supplemental information

Improved oncolytic and immunostimulatory activity of the spontaneous *jin-3* reovirus mutant in preclinical bladder cancer models

Arjanneke F. van de Merbel, Maaike H. van der Mark, Lobke C.M. Hensen, Diana J.M. van den Wollenberg, Rob C. Hoeben, Willemijn C.G. Zonneveld, Rob C.M. Pelger, Maxime T. M. Kummeling, Geertje van der Horst, and Gabri van der Pluijm

Figure S1

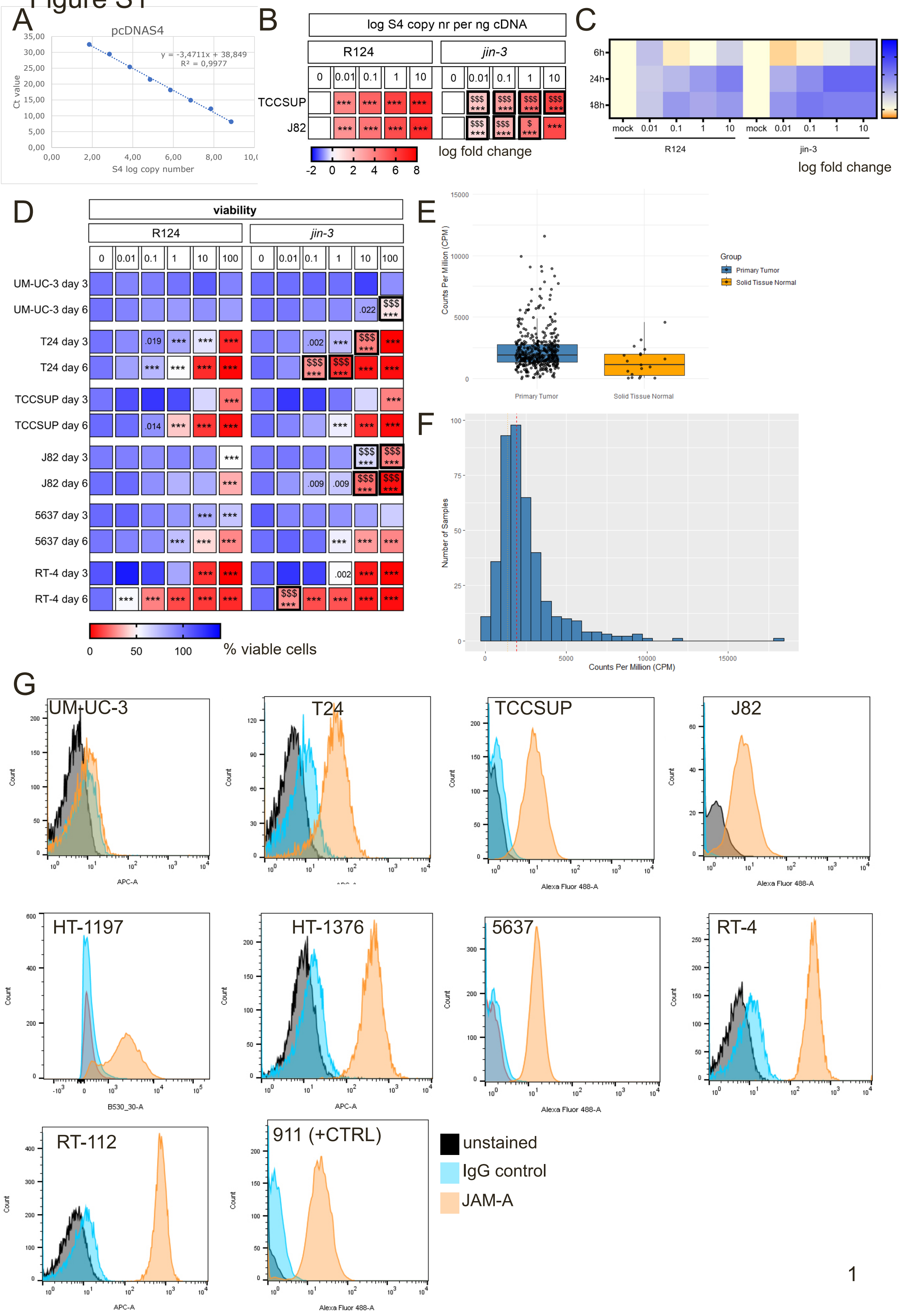
A
pcDNAS4
y = -3,4711x + 38,849
R² = 0,9977
Ct value
S4 log copy number
B
log S4 copy nr per ng cDNA
R124
jin-3
TCCSUP
J82
log fold change
C
R124
jin-3
log fold change
D
viability
R124
jin-3
UM-UC-3 day 3
UM-UC-3 day 6
T24 day 3
T24 day 6
TCCSUP day 3
TCCSUP day 6
J82 day 3
J82 day 6
5637 day 3
5637 day 6
RT-4 day 3
RT-4 day 6
% viable cells
E
Counts Per Million (CPM)
Group
Primary Tumor
Solid Tissue Normal
F
Number of Samples
Counts Per Million (CPM)
G
UM-UC-3
T24
TCCSUP
J82
HT-1197
HT-1376
5637
RT-4
RT-112
911 (+CTRL)
unstained
IgG control
JAM-A

Figure S1

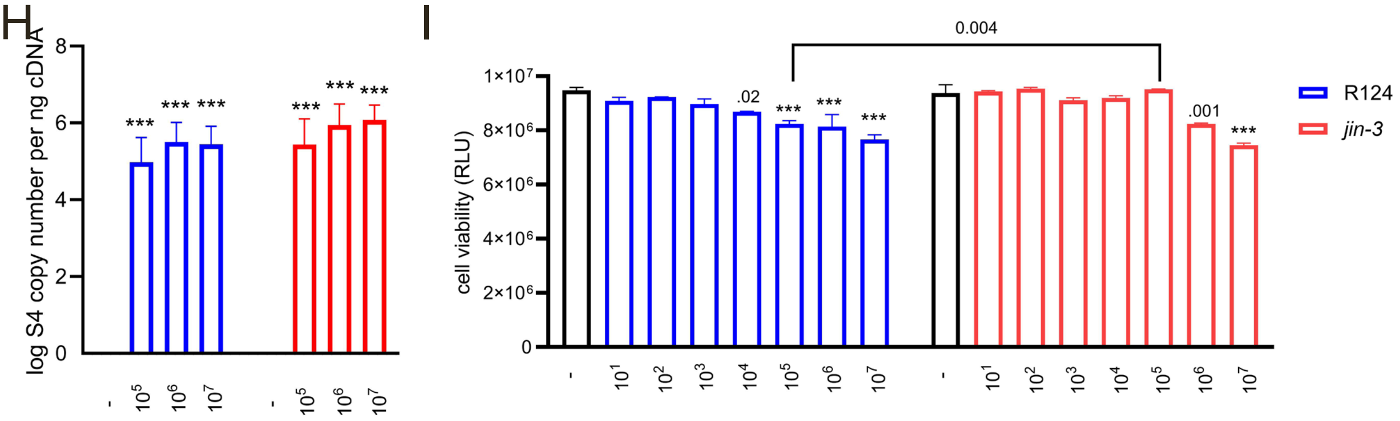

Figure S1: Comparison of the oncolytic effects of reovirus mutant *jin-3* versus wildtype reovirus R124 in bladder cancer cell lines *in vitro* 2D: viral copy numbers.

(A) Standard curve of pcDNA S4 for determination of viral copy number. (B) Heatmap of the viral load (log fold change S4Q mRNA expression vs mock treated cells) in bladder cancer cell lines TCCSUP and J82 exposed to a range of MOI (0.01-0.1-1-10) of either R124 or *jin-3* reoviruses after 24h. (C) Heatmap of the viral load (log fold change S4Q mRNA expression vs mock treated cells) in bladder cancer cell line RT-4 exposed to a range of MOI (0.01-0.1-1-10) of either R124 or *jin-3* reoviruses after respectively 6h, 24h or 48h. Mean (SD) and P values are depicted (vs mock; when 2 depicted the upper P value is comparison R124 vs *jin-3)* *** p<.001, $$$ p<.001, asterisks indicate mock versus reovirus infection, dollar signs R124 versus *jin-3*., N=2 (2 replicates). Two-way ANOVA followed by Tukey's posthoc comparison. (D) Heatmap of mean percentage of viable cells after exposure to a range of MOI of either R124 or *jin-3* for 6 days. N=3 (6 replicates). P values are depicted (vs mock; when 2 depicted upper P value is R124 vs *jin-3*) *** p<.001, $$$ p<.001, asterisks indicate mock versus reovirus infection, dollar signs R124 versus *jin-3*. MOI = multiplicity of infection. (E) Expression analysis of JAM-A/F11R in normal bladder vs primary bladder cancer (counts per million in TCGA-BLCA). (F) Distribution of the counts per million (CPM) of F11R (JAM-A) in TCGA-BLCA primary bladder tumors. (G) fluorescence intensity histograms of the indicated established cell lines stained for JAM-A. (H) viral load (log fold change S4Q mRNA expression vs mock treated cells) in 3D tumoroids of the bladder cancer cell line RT-112 exposed to a range of either R124 or *jin-3* reoviruses (10^5-10^7 pfu) after 3 days of exposure. N=2 (2 replicates). Two-way ANOVA followed by Tukey's posthoc comparison. (I) cell viability was measured using cell titer glo 3D for RT-112 bladder tumoroids after 3 days of treatment with a dose range of viruses.*** p<.001, asterisks indicate mock versus reovirus infection.

Figure S2

A

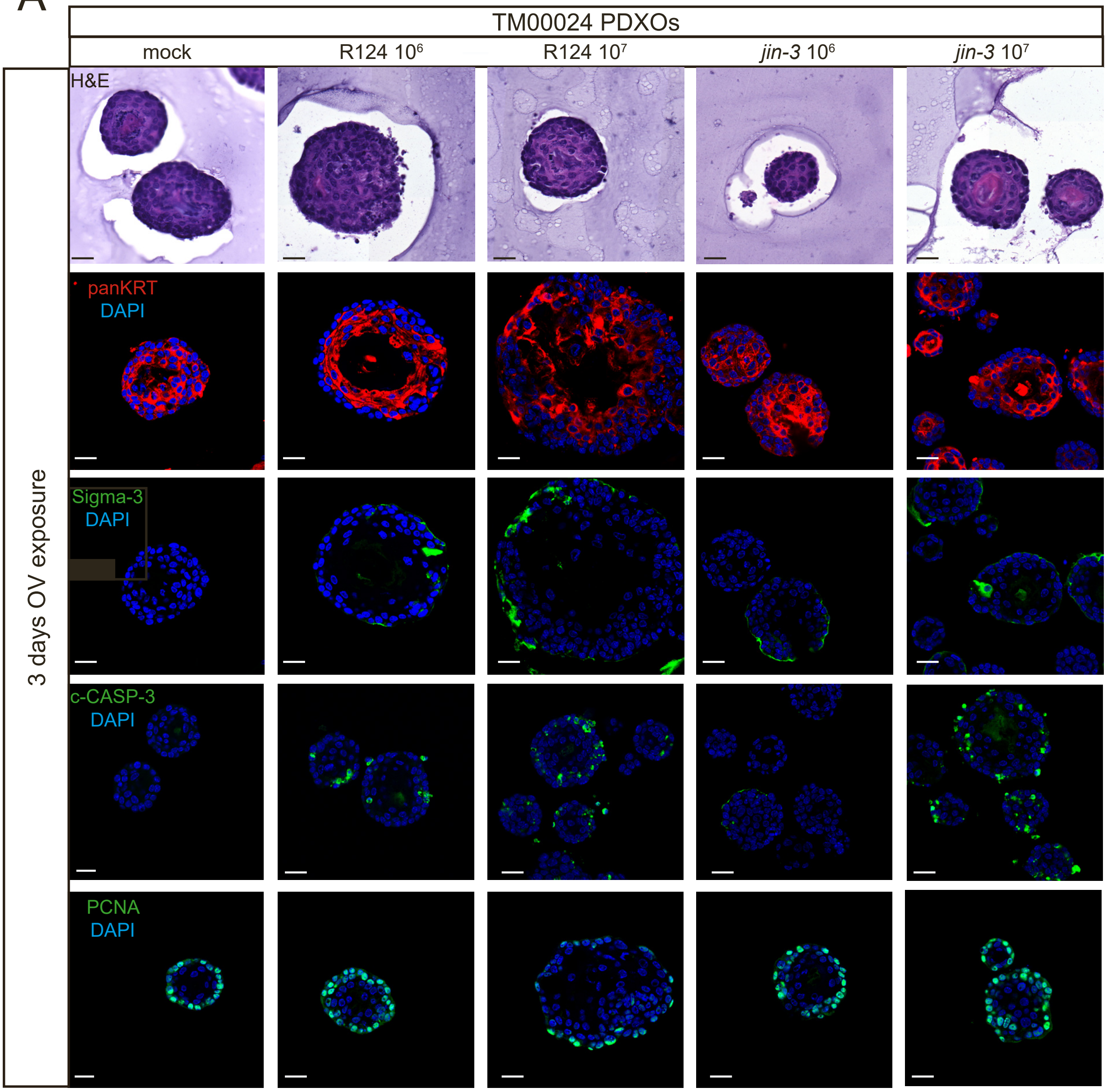
TM00024 PDXOs
mock
R124 10⁶
R124 10⁷
jin-3 10⁶
jin-3 10⁷
3 days OV exposure
H&E
panKRT
DAPI
Sigma-3
DAPI
c-CASP-3
DAPI
PCNA
DAPI

Figure S2

B

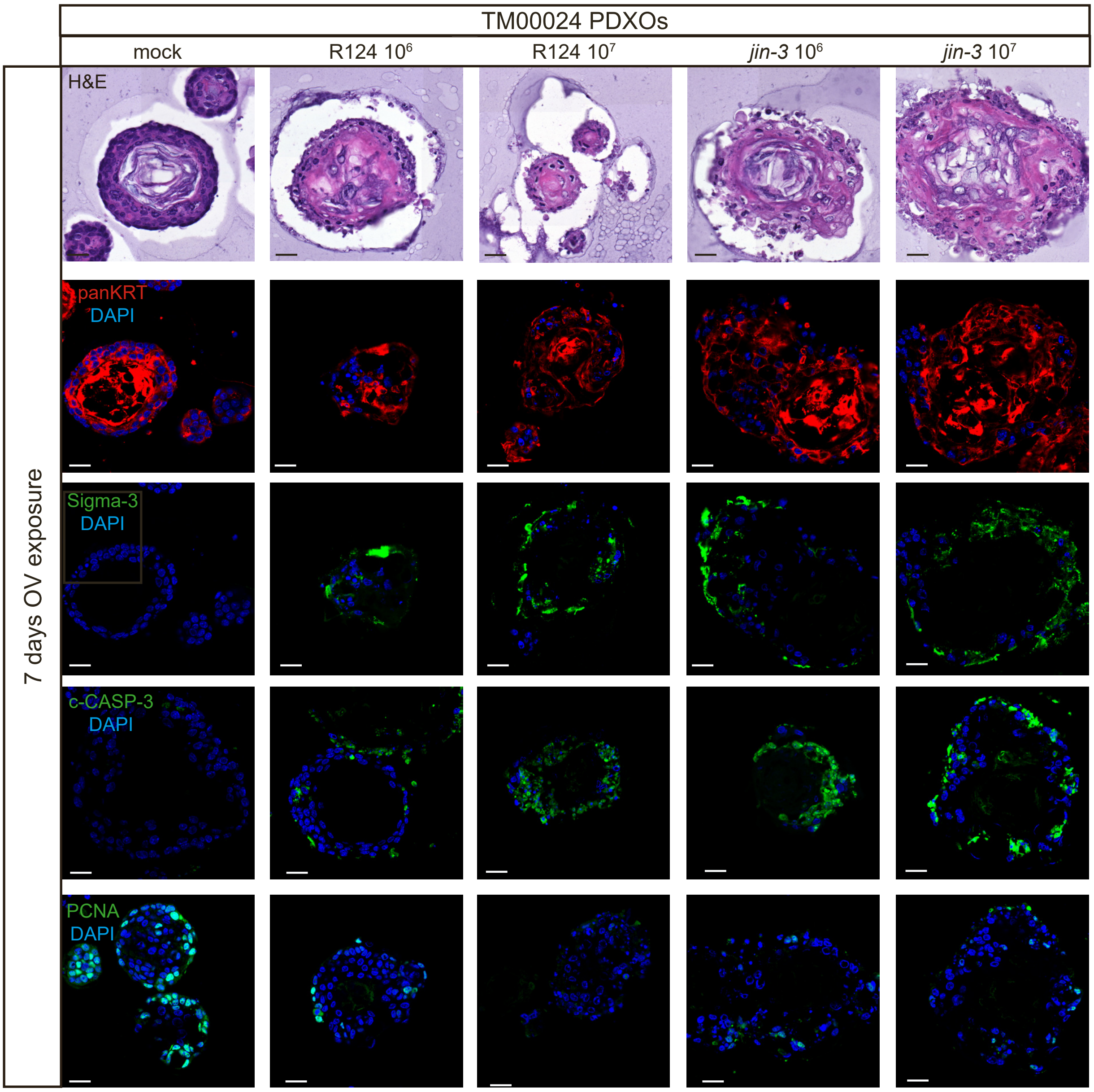

C

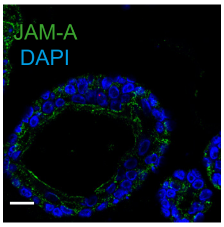

Figure S2 Comparison of the oncolytic effects of reovirus mutant *jin-3* versus wildtype reovirus R124 in PDXOs *in vitro*
TM00024 bladder PDXOs were treated with the indicated pfu of reoviruses for either 3 (A) or 7 days (B). Confocal images of PDXOs are shown stained for respectively H&E, panKRT (red); Sigma-3, c-CASP3 or PCNA (green) and DAPI (blue). Images of day 7 PDXOs treated with either mock,or 10^7 pfu concentrations of both viruses have been reused from figure 2C, that depicted only the highest dosage of virus. Scale bar 20 µm. (C) Confocal images of JAM-A expression (JAM-A in green, and DAPI in blue) in TM00024 PDXOs.

Figure S3

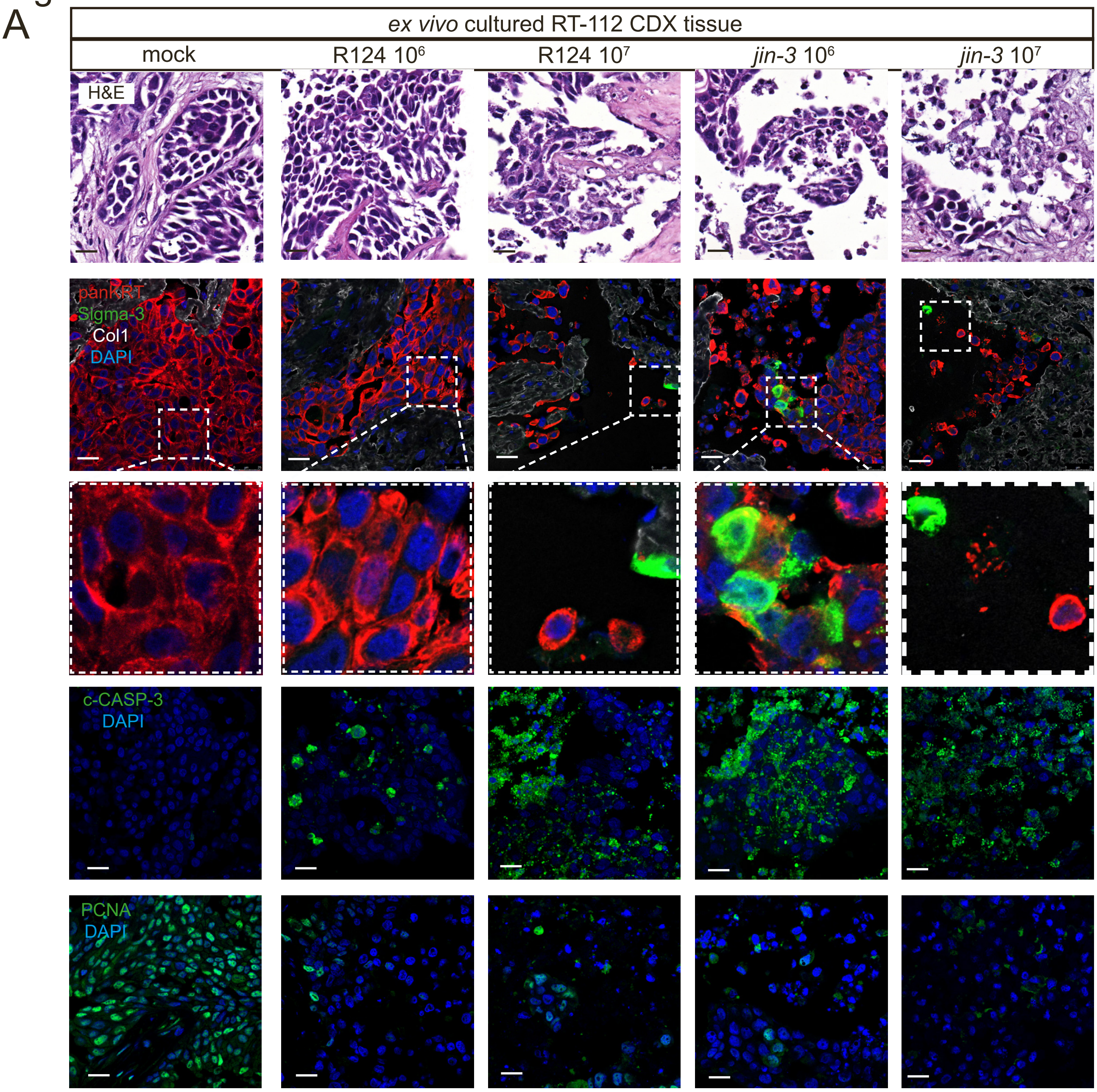

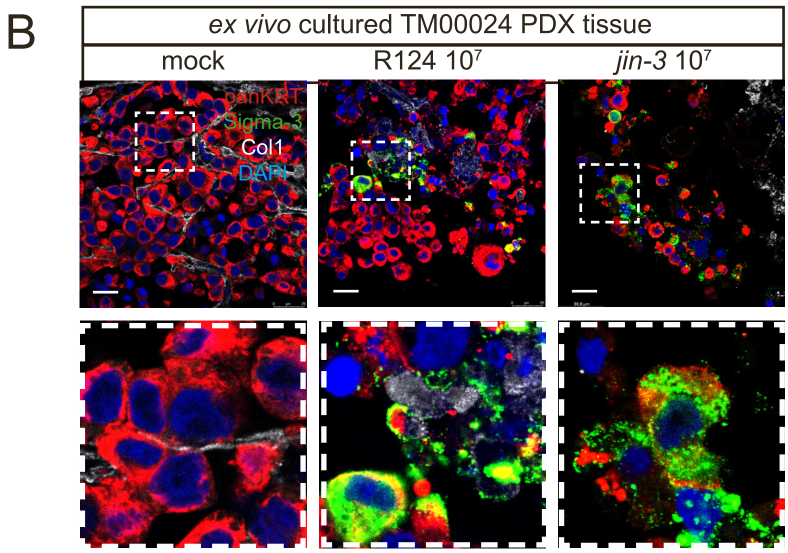

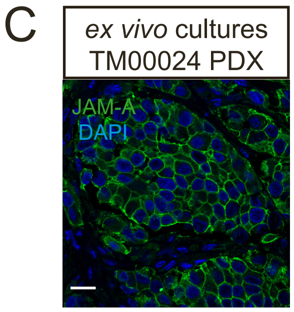

Figure S3 Comparison of the oncolytic effects of reovirus mutant *jin-3* versus wildtype reovirus R124 in *ex vivo* cultured bladder cancer tissue.
Explanted tissue slices from either (A) RT-112 CDX or (B) TM00024 PDX were exposed to the indicated pfu of R124 or *jin-3* reovirus for 3 days. Tissues were stained for HE, panKRT (red); Sigma-3, c-CASP3 or PCNA (green), collagen type I (white) and DAPI (blue) and representative confocal images are shown. Images of *ex vivo* cultured RT-112 CDX treated with either mock,or10^7 pfu concentrations of both viruses have been reused from figure 3A, that depicted only the highest dosage of virus. Images of *ex vivo* cultured TM00024 PDX have been reused from figure 3F. Scale bar 25 μm. (C) Confocal images of JAM-A expression (JAM-A in green, and DAPI in blue) in *ex vivo* cultured TM0024 tissue.

Figure S4

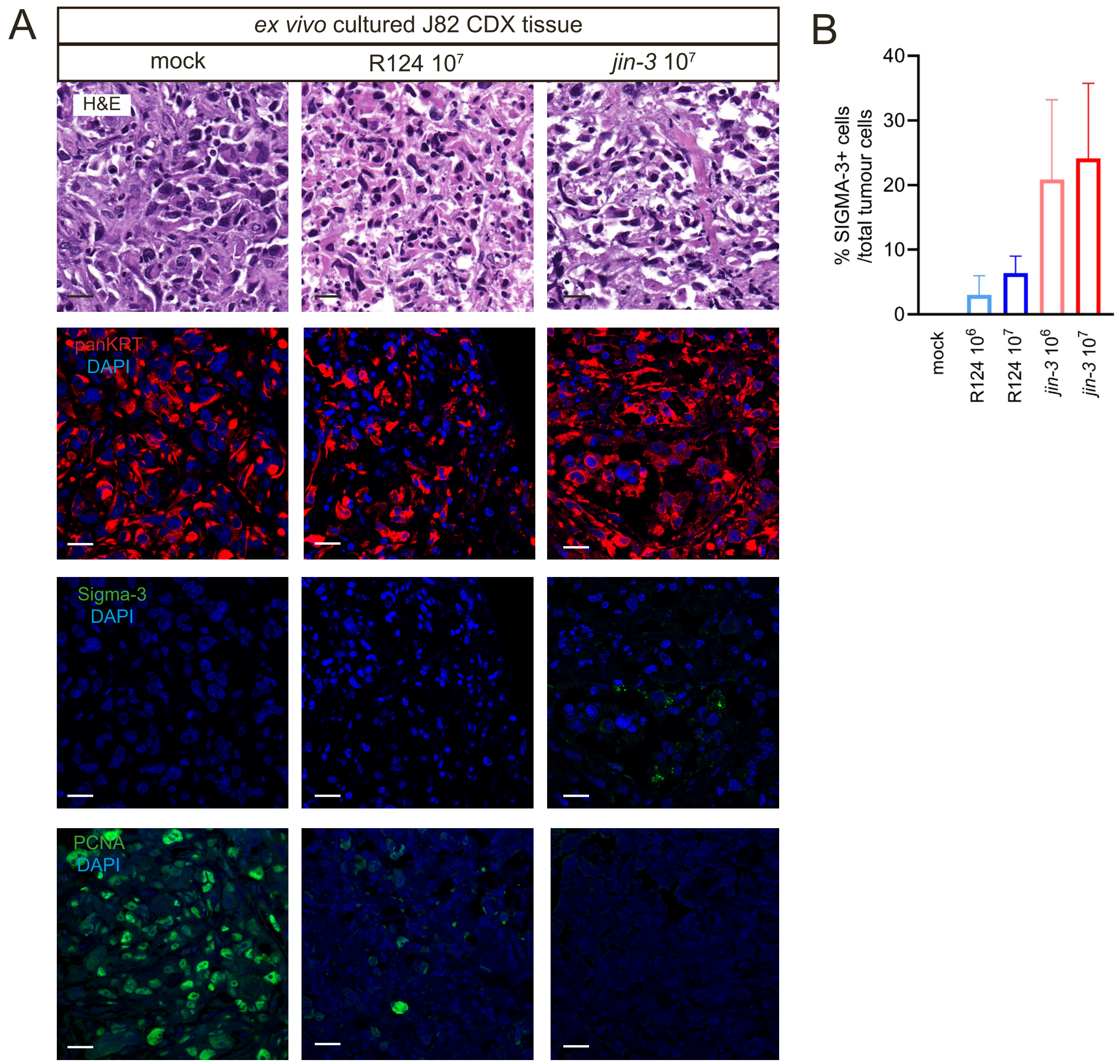

Figure S4 Comparison of *jin-3* and R124 reovirus infection in *ex vivo* cultured tumour tissue slices from cell-line derived xenograft J82
Explanted tissue slices from J82 CDX model were exposed to 10^6 or 10^7 pfu R124 or *jin-3* reovirus for 3 days. For each condition, multiple tissue slices were stained for H&E, panKRT (red), PCNA and Sigma-3 (green) and representative confocal images of the mock and the 10^7 pfu conditions are shown in (A). Scale bar = 20μm. Number of cells with Sigma-3 expression were counted with ImageJ and divided by the number of panKRT+_DAPI+ cells (B). At least 4 fields were scored per technical replicate. Mean (SD) of N=2 (4 replicates).

Figure S5

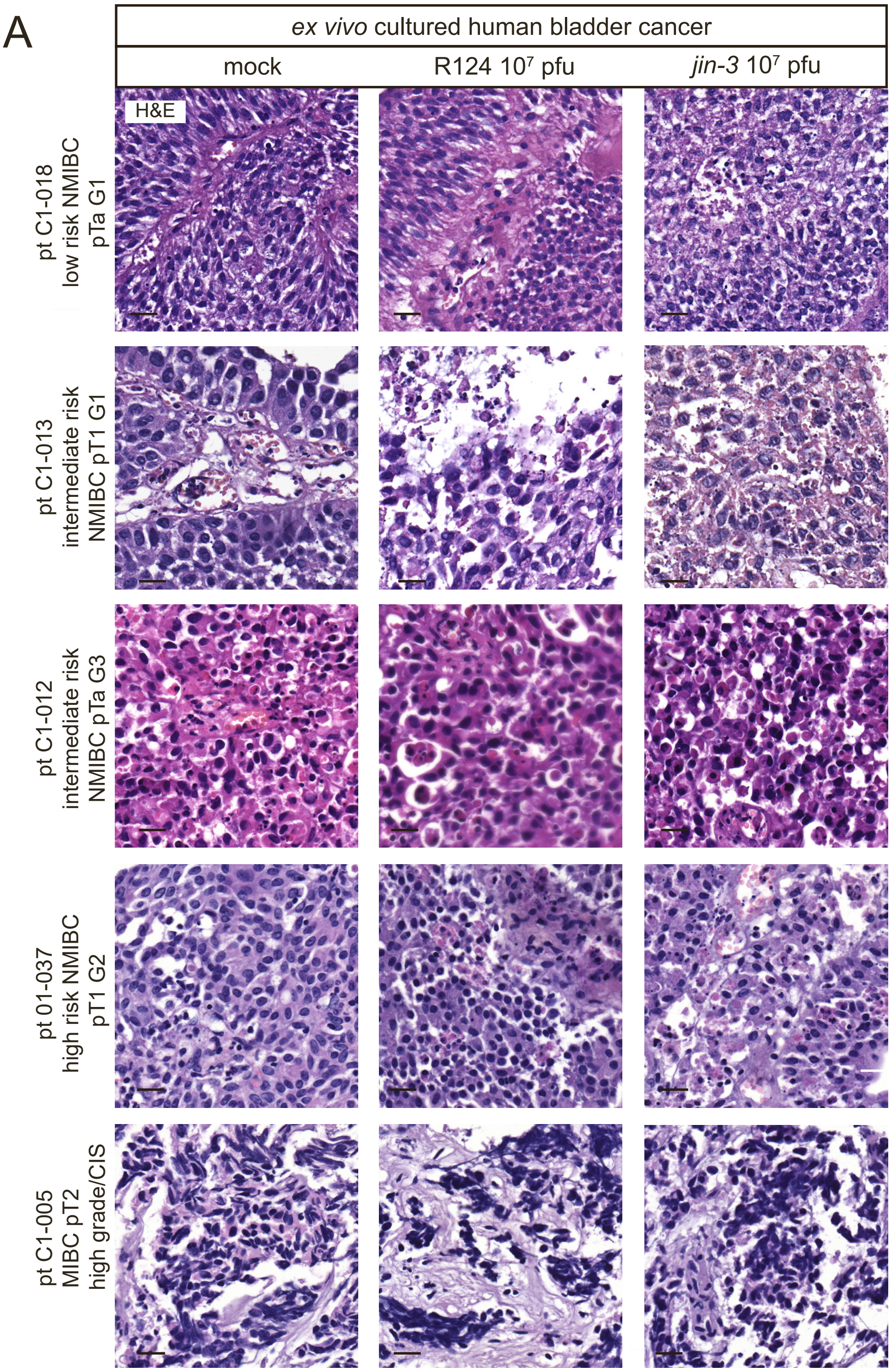
A
ex vivo cultured human bladder cancer
mock
R124 10⁷ pfu
jin-3 10⁷ pfu
H&E
pt C1-018 low risk NMIBC pTa G1
pt C1-013 intermediate risk NMIBC pT1 G1
pt C1-012 intermediate risk NMIBC pTa G3
pt 01-037 high risk NMIBC pT1 G2
pt C1-005 MIBC pT2 high grade/CIS

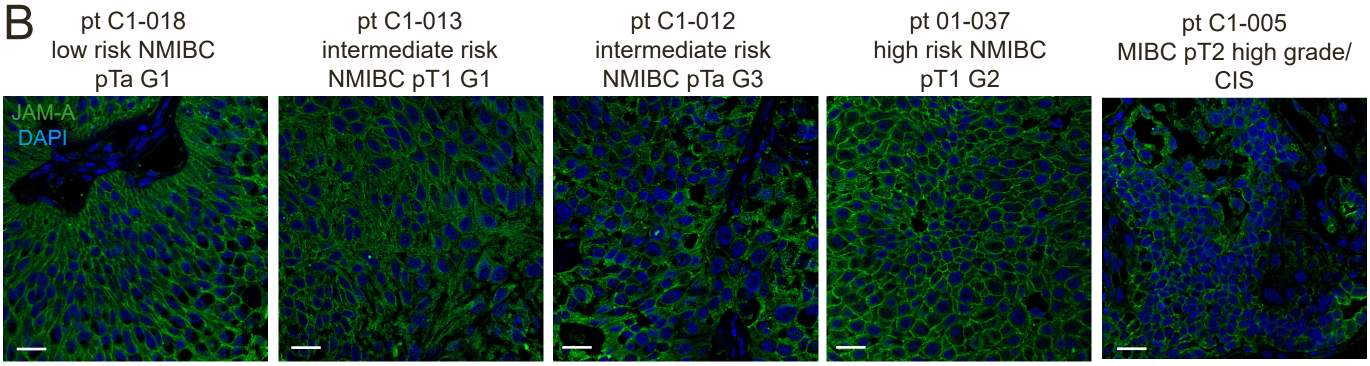
B
pt C1-018 low risk NMIBC pTa G1
pt C1-013 intermediate risk NMIBC pT1 G1
pt C1-012 intermediate risk NMIBC pTa G3
pt 01-037 high risk NMIBC pT1 G2
pt C1-005 MIBC pT2 high grade/ CIS
JAM-A
DAPI

Figure S5

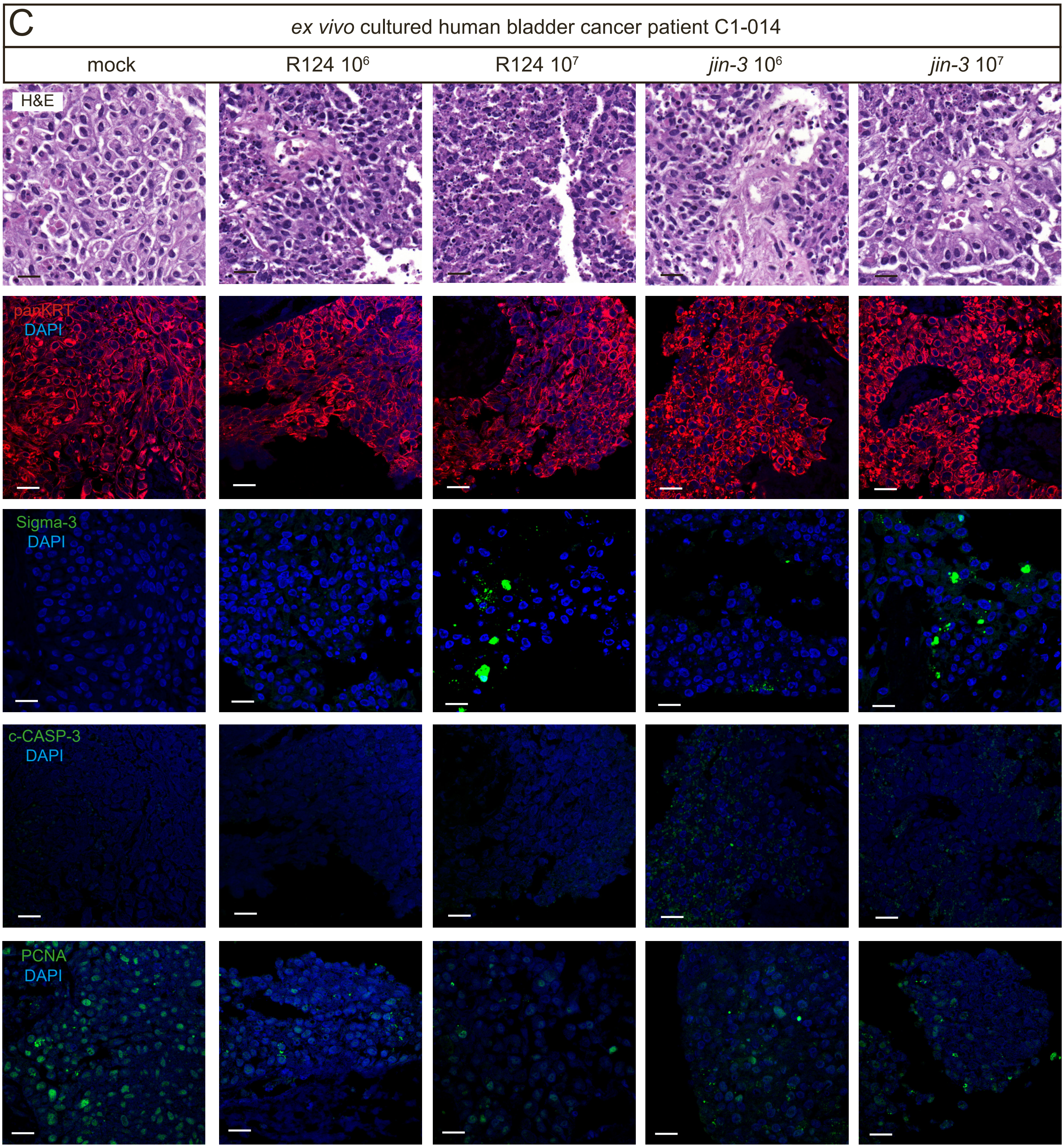

Figure S5: Comparison of *jin-3* and R124 reovirus infection in *ex vivo* cultured tumour tissue slices from human bladder cancer patients
Explanted tissue slices from patients (n=15) diagnosed with bladder cancer were exposed to either mock or reovirus R124 or *jin-3* for 3 days (dependent on the amount of tissue, either a complete dose range (mock, 10^6 and 10^7 pfu) or only the mock and 10^7 dosage). Tissues were stained for H&E. Scale bar = 25 μm. Multiple tissue slices were cultured per condition, representative images from 5 patients are shown. (B) Confocal images of JAM-A expression (JAM-A in green, and DAPI in blue) in mock treated *ex vivo* cultured patient tumour slices (n=5 are shown). (C) Representative confocal images from 1 patient are shown (pt C1-014; low risk NMIBC pTa low grade). Viral infection and replication in *ex vivo* cultured tissue slices with either R124 or *jin-3* reovirus. Green = either Sigma-3 (viral protein), c-CASP-3 (apoptotic cells) or PCNA (proliferating cells), Red = pan-cytokeratin (tumour cells), blue = DAPI (nuclei). Scale bar = 25 μm.

Figure S6

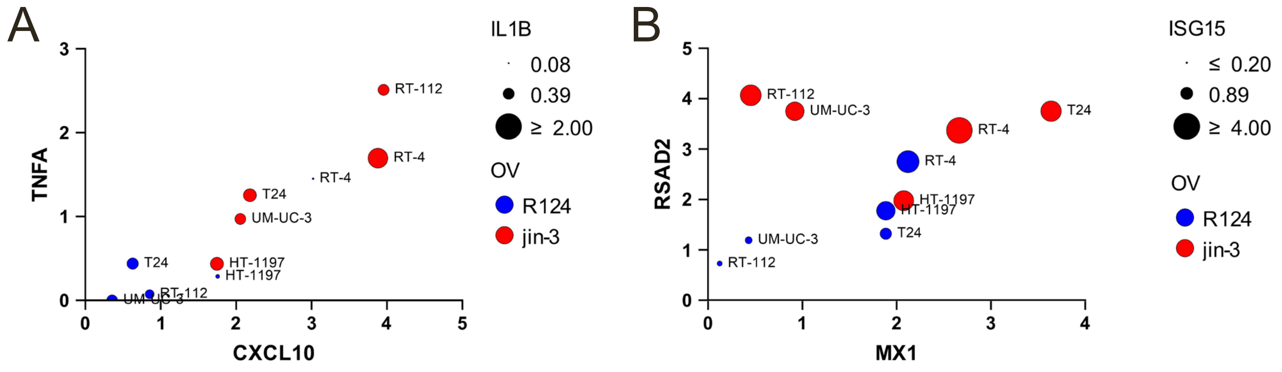

Figure S6 Comparison of immune modulation induced by *jin-3* and R124 reovirus treatment of bladder cancer cell lines
(A) correlation file of mRNA expression of inflammatory cytokines *CXCL10* (x-axis), *TNFA* (y-axis) and *IL1B* (size of the dots) at MOI 10 of the indicated virus. (B) correlation file of mRNA expression of interferon stimulated genes *MX1* (x-axis), *RSAD2* (y-axis) and *ISG15* (size of the dots) at MOI 10 of the indicated virus.

Figure S7

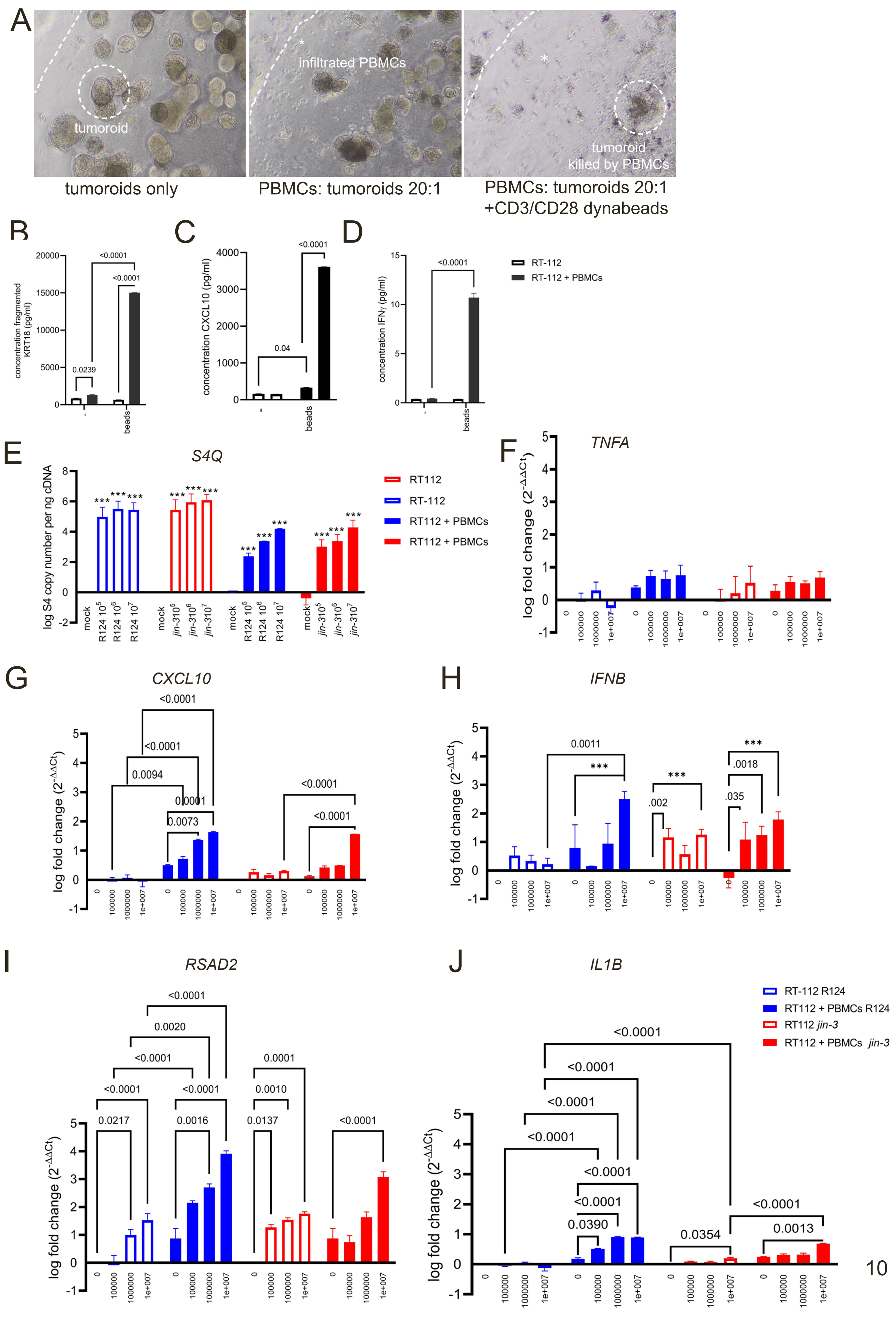
A
tumoroid
infiltrated PBMCs
tumoroid killed by PBMCs
tumoroids only
PBMCs: tumoroids 20:1
PBMCs: tumoroids 20:1 +CD3/CD28 dynabeads
B
concentration fragmented KRT18 (pg/ml)
beads
C
concentration CXCL10 (pg/ml)
D
concentration IFNγ (pg/ml)
RT-112
RT-112 + PBMCs
E
S4Q
log S4 copy number per ng cDNA
RT112
RT-112
RT112 + PBMCs
RT112 + PBMCs
F
TNFA
log fold change (2-ΔΔCt)
G
CXCL10
H
IFNB
I
RSAD2
J
IL1B
RT-112 R124
RT112 + PBMCs R124
RT112 jin-3
RT112 + PBMCs jin-3

Figure S7

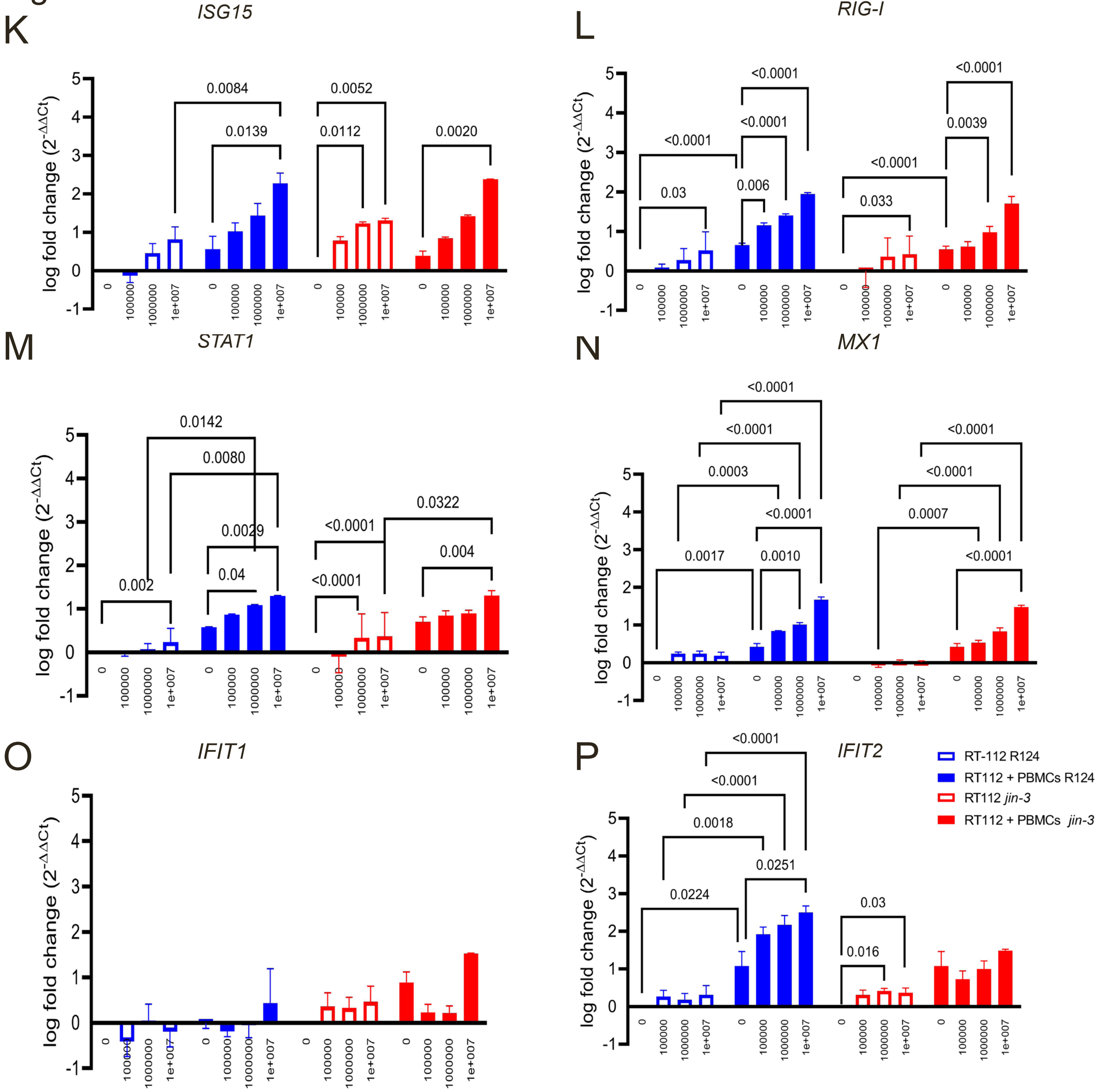

Figure S7 Viability and Immune modulation in RT-112 3D - PBMC co-culture models
(A) RT-112 bladder cancer cells were cultured in 60% matrigel and allowed to form 3D structures for approximately 3 days. Subsequently, PBMCs were added at an effector: target ratio of 20:1 in absence or presence of CD3/CD28 beads to activate the PBMCs. (B) fragmented epithelial marker KRT18 was determined as an outcome measure for tumour cell killing after 3 days of OV exposure. (C) CXCL10 levels and (D) IFNγ levels were measured after 3 days of OV exposure. (N=3, 2 replicates) Mean (SD). Two way ANOVA with Tukey's posthoc comparison (E) viral load (log fold change *S4Q* mRNA expression vs mock treated cells) in 3D bladder cancer cell line RT-112 exposed to a range of either R124 or *jin-3* reoviruses (10^5-10^7 pfu) in presence or absence of PBMCs. Expression levels of various inflammatory cytokines and interferon stimulated genes (log fold change mRNA expression ($2^{-\Delta\Delta Ct}$) vs mock treated cells) in 3D cultured RT-112 cells or RT-112 cells co-cultured with PBMCs exposed to either R124 or *jin-3* reoviruses. (F) *TNFA*, (G) *CXCL10*, (H) *IFNB*, (I) *RSAD2*, (J) *IL1B*, (K) *ISG15*, (L) *RIG-I*, (M) *STAT1*, (N) *MX1*, (O) *IFIT1*, (P) *IFIT2*. mean (SD) N=2. Two-way ANOVA followed by posthoc Tukey's.

Table S1 key resources (provided Excel file)
Key reagents and resources used in the manuscript with their respective suppliers, Research Resource Identifiers (RRID) and additional information. Cells were routinely cultured in a humidified incubator at 37°C and 5% CO2 and were regularly (once every 2 months) tested for mycoplasm by RT-PCR. Supplements were from Life Technologies, Gibco. For used antibodies, respective dilutions were added and the indicated time in the pressure cooker (PC) when antigen retrieval was performed by cooking the slides in unmasking solution (Vector Labs, H-3300); N.A. not applicable.

Supplemental Material and Methods

Virus production

Wildtype T3D reovirus strain R124 was plaque-purified from the wildtype reovirus T3D (ATCC) on HER911 cells.[1, 2] Reovirus mutant *jin-3* was isolated from JAM-A deficient U118MG cells after passaging of the wildtype T3D strain R124.[1] Both R124 and *jin-3* reoviruses were propagated, purified and titrated on human HER911 cells as previously described.[1]

Cell lines and culture conditions

<u>Monolayer culture conditions</u>
All human bladder cancer cell lines were cultured in monolayers with the respective culture medium and supplements (Supplemental table S1). Cells were incubated at 37°C in a humidified incubator with 5% CO2 and passaged every 3-4 days with fresh medium. Cells were regularly (once every 2 months) tested for mycoplasm by qRT-PCR.

<u>3D tumoroid mono-culture conditions</u>
Three-dimensional bladder cancer models were cultured from cells derived from tumours grown in PDX TM00024 model (PDXOs) [3] (Jackson Laboratory) or from RT-112 cells (tumoroids). Cells were seeded in domes at a density of 10,000 per 40 µL in 100% growth factor reduced, phenol red free, matrigel (Corning, 356231). 3D-cultures were incubated in 500 µl medium (respectively organoid culture medium [4] for the TM00024 3D cultures and RPMI supplemented with 10% FCS and P/S and Glutamax for the RT-112 3D cultures) at 37 °C in a humidified incubator with 5% CO2 and medium was refreshed every 3 to 4 days.

<u>3D tumoroid-PBMC co-culture conditions</u>
RT-112 cells were seeded in domes with a cell density of 10,000 cells per 40 µL matrigel (60% growth factor reduced, phenol red free, matrigel (Corning, 356231) with serum free DMEM (GibcoBRL 500ML 10567-014)). 3D-cultures were incubated in 500 µl medium consisting of 1:1 RPMI medium supplemented with 10% fetal bovine serum + P/S and RPMI supplemented with 20% human AB serum + P/S with 25 ng/ml IL-2 (Peprotech). After formation of 3D tumoroids, partially matched PBMCs were added at a 20:1 effector: target ratio. Reoviruses were added at the indicated dosages and the 3D co-cultures were cultured for an additional 3 days.

<u>*Ex vivo* cultured tissue explants</u>
Bladder cancer tissue derived from either CDX, PDX or directly from patient tumour material was sliced and cultured as previously described.[5] Slices were exposed to R124 or *jin-3* reovirus at the indicated dosages. Three days post exposure, the tissues were fixed with 4% PFA and processed for histology.
Tumour tissues from patients diagnosed with various stages of bladder cancer were obtained during transurethral resection of the bladder upon informed consent (table1, supplemental table S1; Biobank urogenital tumours protocol B20-010 evaluated by the METCC Leiden-The Hague-Delft). Criteria for the inclusion of patient derived tissue were 1) diagnosis of UCB in tissue slices (TS) is confirmed by the pathology report; 2) majority of directly fixed and/or mock-treated TS contain tumour cells; 3) size of obtained material is sufficient for at least 3 replicates per

condition.

Cell viability assay

Monolayer experiments viability

Using the CellTiter 96® AQueous One Solution Cell Proliferation Assay (Promega RRID:SCR_006724), viability was measured for all cell line/virus combinations over a range of multiplicity of infection (MOI). 1500 cells per well were seeded in a 96 wells plate. After 24 hours, these cells were exposed to either R124 or *jin-3* (control (mock) solution, (MOI) 0.01, 0.1, 1, 10 and or 100 plaque forming units (PFU)/cell). Cells were incubated for 72 or 144h, after which an MTS assay was performed according to manufacturer's directions. After 2h of incubation time, optical density was measured at 490nm using SpectraMax iD3 (Molecular Devices, San Jose, California USA) plate reader to assess cell viability.[6]

3D culture viability

Using the 3D CellTiter 96® Glo assay (Promega, G9681), viability of 3D-cultures was measured after R124 or *jin-3* infection. After 3 days of OV exposure, 250 µl of both culture medium and CellTiter glo were added to the wells. After 30 minutes incubation at room temperature on a plate shaker (300-500 rpm), luminescence was measured using the SpectraMax iD3 (Molecular Devices, San Jose, California USA) plate reader (integration time 1000ms/well, shake prior to measurement).

Flow cytometry

To assess levels of viral protein expression (Sigma-3), apoptosis (c-CASP-3), JAM-A and/or protein expression of several DAMPs (cell surface expression of calreticulin and HSP90) for each cell line/virus combination, 400,000 cells/well were seeded in a 6-wells plate. Each cell line was exposed to either R124 or *jin-3* (vehicle solution only or MOI 10 plaque forming units (PFU)/cell) and incubated for 48 hours. Subsequently, a single cell suspension of 100,000 cells/well was inserted in 96-wells V-bottom plates.

For the intracellular staining, cells were fixated in 4% paraformaldehyde (PFA) fixation buffer for 20 minutes at room temperature and washed using 1x ISP-buffer. Subsequently, cells were stained with Rabbit-anti-c-CASP-3 and mouse-anti-Sigma-3, with the unstained control cells receiving ISP-buffer only. Cells were incubated in the dark for 30 minutes on ice and afterwards washed twice using 1x ISP buffer. Cells were incubated with the secondary antibodies goat-anti-rabbit-APC and Donkey anti-Mouse IgG2a-Alexa Fluor 488 (with exception of the unstained conditions that only received ISP buffer) in the dark for 30 minutes on ice after which cells were washed and collected in ISP-buffer for flow cytometry.

For the cell surface staining, all cell line/virus combinations were stained for the following: control/unstained, IgG control, Calreticulin, HSP90 or JAM-A. All conditions except the unstained control were stained using live/dead aqua staining (LIVE/DEAD™ Fixable Aqua ARC Dead Cell Stain). After washing the single cell suspension with PBS, live/dead aqua staining was added and cells were incubated at room temperature for 20 minutes. The cells were then washed using FACS buffer (PBS, 1% FCS, 0.1% NaN3, 2 mM EDTA) and Fc receptor blocking was added, after which the plate was incubated for 10 minutes at 4°C. After washing with FACS buffer, IgG, Calreticulin and HSP90 primary antibodies were added to the appropriate wells with only FACS buffer for the control condition. This was incubated in the dark for 30 minutes on ice. Cells were then washed using FACS buffer and secondarily stained using Goat-anti-Rabbit APC. After 30 minutes incubation in the dark on ice, cells were washed with FACS buffer and fixed using PFA fixation buffer over 20 minutes at room temperature. Cells were then washed and collected in FACS buffer for flow cytometry.

Flow cytometry was performed using the LSR Fortessa™ X-20 Cell Analyzer (BD Biosciences Franklin Lakes, NJ United States) and data was analysed using FlowJoTM v10.8.1 (BD Biosciences Franklin Lakes, NJ, United States). For details about antibodies and dilutions used, see table S1

Histology and Immunofluorescent scoring

H&E and immunofluorescent stainings were executed as previously described (Supplemental table S1).5 H&E and Sigma-3 immunofluorescent stainings were scored using the Pannoramic MIDI slidescanner (3DHISTECH). All fluorescent stainings were visualized by confocal microscopy (63x magnification, resolution 1024x1024) (Leica SP8). Immunofluorescent stainings were scored by two independent reviewers. Multiple tissue slices were cultured per condition. At least 4 fields were scored per tissue slice. The average of these 4 fields was shown for each of the technical replicates. Cells expressing the respective proteins were counted with ImageJ and divided by the amount of panKRT+_DAPI+ cells (i.e. intact tumour cells with intact nuclei). The ratio fragmented tumour cells was measured by the number of fragmented cells divided by the number of total tumour cells.

Immunocytochemistry

Cells were seeded in 8 Chamber Slides at a density of 2000 cells/chamber. Cells were fixated for 10 minutes after 24, 48 and 72h of virus exposure at MOI 0.01, 0.1, 1, 10 and 100 in 4% PFA and stored at 4 degrees. Thereafter, cells were stained with a mouse anti-reoviral protein Sigma-3 primary antibody, followed by secondary staining with donkey-anti Mouse Alexa Fluor 488 (Thermo Fisher Scientific; for details see supplemental table S1). IgG isotype was used as a control staining. Subsequently, all wells were stained with DAPI. Immunofluorescence was visualized with confocal microscopy (SP8, Leica).

Real-time quantitative polymerase chain reaction (RT-qPCR)

Cells were seeded in 6 well plates and exposed to R124 or *jin-3* for 24 and 48 hours. Total RNA was isolated according to the manufacturer's protocol (Nucleospin RNA kit Macherey-Nagel). cDNA was generated by using random primers (Promega) and RT-qPCR was performed with GoTaq Mastermix (Promega) according to the manufacturer's protocol in technical duplicates and biological triplicates (Promega). Gene expression was normalized to GAPDH (∆CT). Fold change was measured using 2-∆∆Ct compared to mock treated sample. Primer sequences can be found in supplemental table S1. Viral S4Q copy number was calculated using the pcDNA S4 vector (supplemental figure S1B). 7

ELISAs

High Mobility Group Box 1 (HMGB1)-release

Cells were exposed to R124 or *jin-3* with a MOI of 10. HMBG1 release was measured by performing an Lumit™ HMGB1 Human/Mouse Immunoassay according to the manufacturer's protocol. In this assay, a pair of anti-human HMGB1 monoclonal antibodies were covalently labelled with complementary Small BiT (SmBiT; 11 amino acids) or Large BiT (LgBiT; 17.6 kDa) subunits (NanoBiTs) derived from NanoLuc® luciferase. These antibodies were incubated with sample. When the labelled antibodies recognized and bound to human HMGB1, the complementary NanoBiTs were brought into proximity, thereby reconstituting NanoBiT® luciferase and generating luminescence in the presence of Lumit™ Substrate. Light generated is directly proportional to the amount of analyte present in the sample. Luminescence was measured using the SpectraMax iD3 (Molecular Devices, San Jose, California USA) plate reader (37°C, integration time 1000ms/well, shake prior to measurement).

Fragmented KRT18 ELISA

3D cultures were exposed to R124 or *jin-3* at the indicated dosages. At the indicated time post-exposure, conditioned medium was collected and fragmented KRT18 levels were measured using the human cytokeratin 18 Fragment SimpleStep ELISA kit (CK18-30, Abcam).

CXCL10 ELISA
3D cultures were exposed to R124 or *jin-3* at the indicated dosages. At the indicated time post-exposure, conditioned medium was collected and CXCL10 levels were measured with Human IP-10 ELISA (Hu IP-10, Invitrogen).

IFN gamma ELISA
3D cultures were exposed to R124 or *jin-3* at the indicated dosages. At the indicated time post-exposure, conditioned medium was collected and IFNgamma levels were measured with the human IFN-gamma SimpleStep ELISA kit (ab174443, Abcam).

Statistical analyses
Statistical analyses were performed by using GraphPad Prism 8.0. Two-way ANOVA was performed with Tukey posthoc testing for multiple comparison. For *ex vivo* cultures, sample size was calculated using proportional odds models as described 8. Multiple slices were scored per condition per patient with multiple fields per slice.

REFERENCES
1 van den Wollenberg, D.J., Dautzenberg, I.J., van den Hengel, S.K., Cramer, S.J., de Groot, R.J., Hoeben, R.C., (2012). Isolation of reovirus T3D mutants capable of infecting human tumor cells independent of junction adhesion molecule-A. PloS one 7, e48064.
2 Dautzenberg, I.J., van den Wollenberg, D.J., van den Hengel, S.K., Limpens, R.W., Barcena, M., Koster, A.J., Hoeben, R.C., (2014). Mammalian orthoreovirus T3D infects U-118 MG cell spheroids independent of junction adhesion molecule-A. Gene Ther 21, 609-617.
3 Pan, C.X., Zhang, H., Tepper, C.G., Lin, T.Y., Davis, R.R., Keck, J., Ghosh, P.M., Gill, P., Airhart, S., Bult, C., et al., (2015). Development and Characterization of Bladder Cancer Patient-Derived Xenografts for Molecularly Guided Targeted Therapy. PloS one 10, e0134346.
4 Mullenders, J., de Jongh, E., Brousali, A., Roosen, M., Blom, J.P.A., Begthel, H., Korving, J., Jonges, T., Kranenburg, O., Meijer, R., et al., (2019). Mouse and human urothelial cancer organoids: A tool for bladder cancer research. Proc Natl Acad Sci U S A 116, 4567-4574.
5 van de Merbel, A.F., van der Horst, G., van der Mark, M.H., van Uhm, J.I.M., van Gennep, E.J., Kloen, P., Beimers, L., Pelger, R.C.M., van der Pluijm, G., (2018). An ex vivo Tissue Culture Model for the Assessment of Individualized Drug Responses in Prostate and Bladder Cancer. Front Oncol 8, 400.
6 van de Merbel, A.F., van der Horst, G., van der Mark, M.H., Bots, S.T.F., van den Wollenberg, D.J.M., de Ridder, C.M.A., Stuurman, D., Aalders, T., Erkens-Schulz, S., van Montfoort, N., et al., (2021). Reovirus mutant jin-3 exhibits lytic and immune-stimulatory effects in preclinical human prostate cancer models. Cancer Gene Ther.
7 Mijatovic-Rustempasic, S., Tam, K.I., Kerin, T.K., Lewis, J.M., Gautam, R., Quaye, O., Gentsch, J.R., Bowen, M.D., (2013). Sensitive and specific quantitative detection of rotavirus A by one-step real-time reverse transcription-PCR assay without antecedent double-stranded-RNA denaturation. J Clin Microbiol 51, 3047-3054.
8 Walters, S.J., (2004). Sample size and power estimation for studies with health related quality of life outcomes: a comparison of four methods using the SF-36. Health Qual Life Outcomes 2, 26.
9 Zuiverloon, T.C.M., de Jong, F.C., Costello, J.C., Theodorescu, D., (2018). Systematic Review: Characteristics and Preclinical Uses of Bladder Cancer Cell Lines. Bladder Cancer 4, 169-183.
